# Ruthenium-Catalyzed Oxidative Synthesis of N-(2-triazine)indoles by C-H Activation

**DOI:** 10.3390/molecules28093676

**Published:** 2023-04-24

**Authors:** Ming Zeng, Jiaqi Chen, Fengye Li, Haojie Li, Lan Zhao, Dengzhao Jiang, Jun Dai, Wenbo Liu

**Affiliations:** School of Pharmacy and Life Science, Jiujiang University, Jiujiang 332005, China18870403447@sina.cn (H.L.);

**Keywords:** ruthenium, C-H activation, triazines, indoles

## Abstract

1,3,5 triazines, especially indole functionalized triazine derivatives, exhibit excellent activities, such as anti-tumor, antibacterial, and anti-inflammatory activities. Traditional methods for the synthesis of N-(2-triazine) indoles suffer from unstable materials and tedious operations. Transition-metal-catalyzed C-C/C-N coupling provides a powerful protocol for the synthesis of indoles by the C-H activation strategy. Here, we report the efficient ruthenium-catalyzed oxidative synthesis of N-(2-triazine) indoles by C-H activation from alkynes and various substituted triazine derivatives in a moderate to good yield, and all of the N-(2-triazine) indoles were characterized by ^1^H NMR, ^13^C NMR, and HRMS. This protocol can apply to the gram-scale synthesis of the N-(2-triazine) indole in a moderate yield. Moreover, the reaction is proposed to be performed via a six-membered ruthenacycle (II) intermediate, which suggests that the triazine ring could offer chelation assistance for the formation of N-(2-triazine) indoles.

## 1. Introduction

Indoles are one of the most important nitrogen heterocycles in medicinal chemistry, natural products, and organic synthesis [1,2,3,4,5,6,7]. Additionally, indoles are termed privileged motifs because of their excellent biological activities and applications in drug discovery. For these reasons, the construction of indoles has attracted considerable attention over the past few decades [8,9,10,11]. Among these creative approaches, the transition-metal-catalyzed C-H activation strategy of indoles has taken a prominent role. Generally, the activation of the C–H bond of alkylenes requires the participation of transition-metal catalysts [12,13,14,15,16,17] and the assistance of directing groups (amide [18,19,20,21], pyridyl [22,23], pyrimidyl [24], imidazole [25], carboxylic ester [26], and methoxy [27]). In particular, Stuart reported the first example of the synthesis of indoles from N-phenyl-2-aminopyridine and alkyne or alkene via intramolecular oxidative C-N coupling and C-H activation by [Cp*RhCl_2_]_2_ [28]. This powerful protocol provided a new insight for chemists to construct indoles by C-H activation from various substrates or with cheaper catalysts. However, in contrast to rhodium, inexpensive ruthenium or nickel complexes are competent choices for these transformations. Recently, many efficient methods for indole derivatives by the Ru-catalyzed oxidative annulation of 6-anilinopurine or 2-aminopyridine and alkynes have been developed. Nevertheless, innovative approaches to diverse indole derivatives still require further exploration.

On the other hand, triazine is a privileged pharmacophore against various targets, especially indole-substituted triazines, and has been reported to be a potential antibacterial [29], anti-inflammation [30,31] agent and an acetylcholinesterase [32] and cyclic GMP-AMP synthase [33] inhibitor. Normally, unstable cyanuric chloride is used as raw material for the preparation of these compounds through a nucleophilic displacement reaction. Hence, it is significant to develop practical protocols that can meet the demand for sustainable development. More recently, Cui and their group reported the efficient copper-catalyzed sulfonamidation of arenes via C-H activation, with triazine as the directing group [34]. Inspired by our previous work [35,36,37,38,39], we reasoned that N-aryl-triazines are suitable substrates for the synthesis of indole-substituted triazines through C-H activation. We herein describe the ruthenium-catalyzed synthesis of N-triazine-substituted indoles from N-aryl-triazines and alkynes.

## 2. Results and Discussion

We commenced our studies by identifying the reaction of N^2^,N^2^-dimethyl-N^4^-phenyl-1,3,5-triazine-2,4-diamine (**1a**) with 1,2-diphenylethyne (**2a**) in the presence of a ruthenium catalyst. We focused our optimization studies on a variety of additives (Cu(OAc)_2_, Cu (NO_3_)_2_, Cu (NO_3_)_2_, CuBr_2_, Cu(CF_3_SO_3_)_2_, NaOAc, CsOAc); the initial screening indicated that the use of Cu(OAc)_2_ proved to be optimal and gave a higher yield ( Table 1, entries 1–7). Notably, additional KBF_6_ to the reaction mixture did not improve the production of the desired product **3a** (Table 1, entry 8). However, this reaction could not proceed without (Cu(OAc)_2_, suggesting that Cu(OAc)_2_ was vital for this transformation (Table 1, entry 9). Decreasing the loading of Cu(OAc)_2_ to 5% resulted in a 19% yield, while a slightly lower yield was observed when 20% of Cu(OAc)_2_ was added (Table 1, entries 10–11). The yield of **3a** improved to 63% when the reaction was conducted at 100 ^o^C (Table 1, entries 12–14). To our delight, a comparable yield (71%) was obtained by increasing the number of equivalents of **2a** (Table 1, entries 15–17). Moreover, the reaction was conducted for 6 h so that it could lead to a lower yield, with many of the starting materials remaining unreacted (Table 1, entry 18). It is worth noting that the unidentified reaction occurred during the operation at 24 h (Table 1, entry 19). We also investigated a set of representative solvents, and DMF was proven to be the best choice for this synthetic methodology (Table 1, entries 20–23). Furthermore, Pd-catalysts were explored as a catalyst system, but the desired product was hardly observed (Table 1, entries 24–25). However, increasing or decreasing the amount of the ruthenium catalyst (10% or 2%) showed less efficiency for the formation of **3a** (Table 1, entries 26–27).

**Scheme 1:**
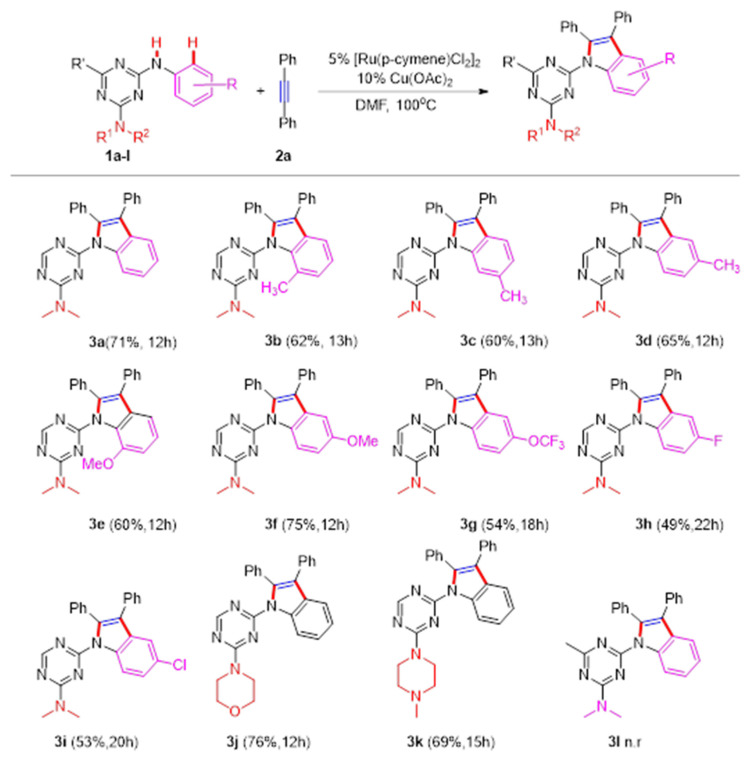
The synthesis of **3** from N-aryl triazines. Reaction conditions: **1a** (1 mmol), **2a** (1.5 mmol), [Ru(p-cymene)CL_2_]_2_ (5 mol%), Cu (OAc)_2_ (10 mol%), and solvent (3 mL), 12h, isolated yield.

With the optimized reaction conditions in hand, a range of N-aryl triazines **1** was subjected to probe the reaction scope and generality (Scheme 1). The substrates with methyl or methoxy groups at different positions of the benzene ring exhibited good reactivity with a yield of up to 75% (**3b**-**3f**). What surprised us was that the corresponding product **3c** obtained a 60% yield to the single isomer when **1c** was applied. This observation indicates that the triazine ring could be an excellent directing group for improving the regioselectivity of this coupling reaction. N-aryl triazines with halogen groups (F, Cl) also underwent a reaction to give the desired products (**3h**, **3i**). Additionally, triazines bearing morpholino or N-methyl piperazine could readily couple with **2a** in 76% and 69% yields, respectively (**3j**, **3k**). However, this reaction did not occur when the hydrogen atom at the C6 position of the triazine ring was replaced by methyl or other groups.

**Scheme 2:**
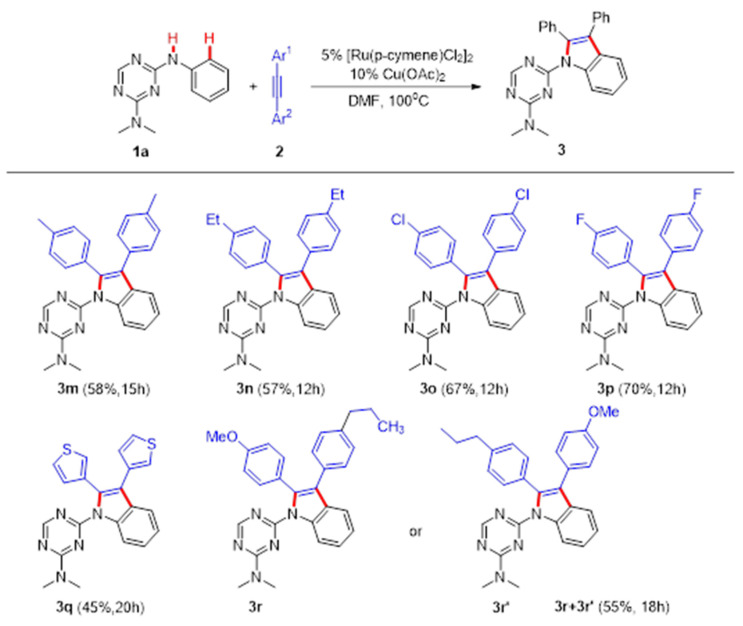
The synthesis of **3** from various alkynes. Reaction conditions: ^a^ **1a** (1 mmol), **2a** (1.5 mmol), [Ru(p-cymene)CL_2_]_2_ (5 mol%), Cu (OAc)_2_ (10 mol%), and solvent (3 mL), isolated yield.

**Scheme 3:**
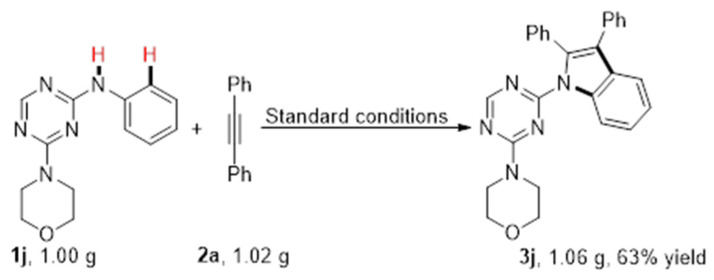
Gram-scale synthesis of N-(2-triazine) indole **3j**.

Then, various alkynes were tested to further investigate the scope of the ruthenium-catalyzed oxidative annulation strategy. Alkynes with electron-donating or withdrawing substituents reacted smoothly with **1a** to generate the corresponding products (Scheme 2, **3m**-**3p**). Furthermore, 1,2-di(thiophen-3-yl)ethyne **1q** readily transformed into the desired product **3q** at a 45% yield (Scheme 2, **3q**). In order to explore the regioselectivity of the C−H annulation process, unsymmetrically substituted alkyne **1r** was performed under optimized conditions. The results showed that **3r** and **3r’** could be obtained with moderate regioselectivity and a total yield of 55% (Scheme 2, **3r** and **3r’**). The reaction of **1j** (1.00 g) with **2a** (1.02 g) under the optimal reaction conditions gave the corresponding product **3j** at a 63% yield (Scheme 3).

**Scheme 4:**
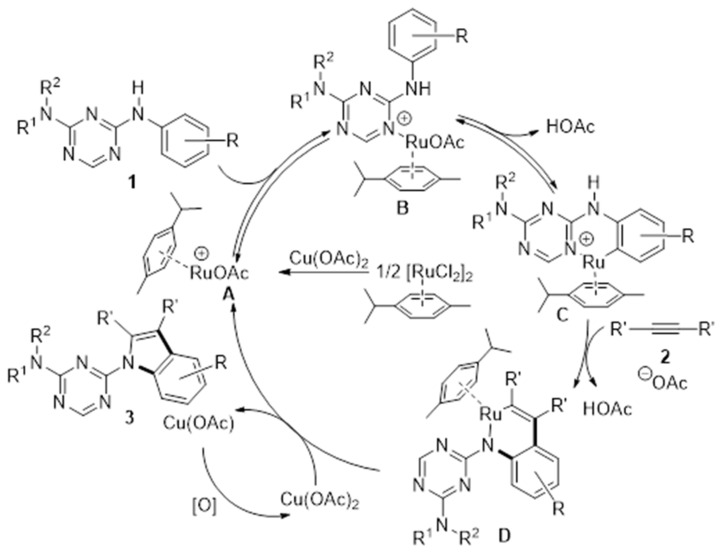
Proposed reaction mechanism.

Based on the previous work [40,41,42,43], we inferred the possible mechanism for ruthenium oxidative annulation approaches. Firstly, cationic ruthenium (II) complex **A,** which was generated in the presence of Cu(OAc)_2,_ reacted with **1** to produce **B**, and six-membered ruthenacycle (II) **C** was observed through the elimination of AcOH from **B** [41]. Subsequently, the formation of **D** was proposed to undergo coordination and migratory insertion with **2** [41,44]. Finally, **D** underwent reductive elimination to furnish the desired product **3** (Scheme 4). However, replacing the hydrogen atom at C6 of the triazine ring with other groups prevented the formation of vital intermediate **B** and **C**. It was clear that no expected corresponding product was observed when there were other groups at C6 of the triazine ring.

## 3. Materials and Methods

### 3.1. General Information

Unless otherwise noted, materials were obtained from commercial suppliers and used without further purification. All reactions were performed in a heating mantle in a sealed tube unless otherwise noted. Thin layer chromatography (TLC) was performed using silica gel 60 F254 and was visualized using UV light. Column chromatography was performed with silica gel (mesh 300– 400). ^1^H NMR and ^13^C NMR spectra were recorded on a Bruker Avance 400 MHz spectrometer in CDCl_3_ with Me_4_Si as an internal standard. Data were reported as follows: a chemical shift in ppm (*δ*), multiplicity (s = singlet, d = doublet, t = triplet, q = quartet, br = broad, and m = multiplet), coupling constant in Hertz (Hz) and integration. The HRMS and mass data were recorded by ESI on a TOF mass spectrometer.

### 3.2. General Procedure for the Synthesis of **1**

The initial compound **1** was prepared as referred to in our previous work [38,39,45]. Sodium (2.5 eq) was added to anhydrous MeOH at 0 °C until the sodium was completely consumed. Then, biguanides hydrochloride (1.0 eq) was added to the reaction mixture, and 3–4 h later, methyl formate (ethyl acetate for 1l) was added. The above reaction mixture was stirred for another 24 h at 40 °C. When the reaction was complete, the mixture was concentrated under reduced pressure, and water was added, filtered, washed with water, and dried to produce a crude product. The crude product was purified by recrystallization in MeOH to obtain **1a**-**1l**.

*N^2^,N^2^-dimethyl-N^4^-phenyl-1,3,5-triazine-2,4-diamine* **(1a)** White solid, yield 2.65 g (74%), m.p: 186.8–187.0 °C; ^1^H NMR (400 MHz, CDCl_3_) δ 8.30 (s, 1H, CH), 7.85 (br, 1H, NH), 7.63 (d, *J* = 7.8 Hz, 2H, Ar-H), 7.36 (t, *J* = 7.8 Hz, 2H, Ar-H), 7.09 (t, *J* = 7.8 Hz, 1H, Ar-H), 3.22 (s, 3H, N-CH_3_), 3.2 (s, 3H, N-CH_3_); ^13^C NMR (100 MHz, CDCl_3_) *δ* 165.4, 164.5, 163.1, 138.7, 128.8, 123.1, 120.2, 36.4.

*4-morpholino-N-phenyl-1,3,5-triazin-2-amine* **(1j)** White solid, yield 1. 79 g (95%), m.p: 171.6–172.9 °C; ^1^H NMR (400 MHz, CDCl_3_) δ 8.35-8.30 (m, 2H, CH, NH), 7.57 (d, *J* = 7.7 Hz, 2H, Ar-H), 7.36 (t, *J* = 7.7 Hz, 2H, Ar-H), 7.11 (t, *J* = 7.7 Hz, 1H, Ar-H), 3.93-3.83 (m, 4H, O-CH_2_-), 3.82-3.73 (m, 4H, N-CH_2_-); ^13^C NMR (100 MHz, CDCl_3_) δ 165.7, 164.0, 163.5, 138.5, 128.8, 123.4, 120.5, 66.6, 43.7.

*4-(4-methylpiperazin-1-yl)-N-phenyl-1,3,5-triazin-2-amine* **(1k)** White solid, yield 1.73 g (90%), m.p: 187.2–188.3 °C; ^1^H NMR (400 MHz, CDCl_3_) δ 8.29 (s, 1H, CH), 7.84 (br, 1H, NH), 7.57 (d, *J* = 7.7 Hz, 2H, Ar-H), 7.35 (t, *J* = 7.7 Hz, 2H, Ar-H), 7.09 (t, *J* = 7.7 Hz, 1H, Ar-H), 3.97-3.81 (m, 4H, N-CH_2_-), 2.57-2.42 (m, 4H, N-CH_2_-), 2.35 (s, 3H, N-CH_3_); ^13^C NMR (100 MHz, CDCl_3_) δ 165.8, 163.8, 163.5, 138.6, 128.8, 123.3, 120.4, 54.7, 46.1, 43.1.

*N^2^,N^2^,6-trimethyl-N^4^-phenyl-1,3,5-triazine-2,4-diamine* **(1l)** [44] White solid, yield 1.65 g (86%), m.p: 149.2–152.1 °C; ^1^H NMR (400 MHz, CDCl_3_) δ 7.63 (dd, *J* = 8.6, 0.9 Hz, 2H, Ar-H), 7.58 (br, 1H, N-H), 7.35-7.29 (m, 2H, Ar-H), 7.05 (t, *J* = 7.4 Hz, 1H, Ar-H), 3.21 (s, 3H, N-CH_3_), 3.20 (s, 3H, N-CH_3_), 2.35 (s, 3H, -CH_3_); ^13^C NMR (100 MHz, CDCl_3_) δ 175.1, 165.3, 163.7, 139.1, 128.7, 122.7, 119.9, 119.8, 36.3, 25.6.

### 3.3. General Procedure for the Synthesis of **3**

Cu(OAc)_2_ (10 mol%). was added to a mixture of N^4^-phenyl-1,3,5-triazine-2,4-diamine derivatives (0.5 mmol), [Ru(p-cymene) Cl_2_]_2_ (5 mol%) in DMF). The resultant mixture was then sealed and stirred for 12–20 h at 100 °C. After the completion of the reaction, the reaction mixture was cooled to room temperature and extracted with ethyl acetate. The organic phase was dried over anhydrous Na_2_SO_4_. The crude residue was obtained after the evaporation of the solvent in a vacuum, and the residue was purified by flash chromatography with petroleum ether and ethyl acetate (*v*/*v* 10/1) as the eluent to provide a pure product.

*4-(2,3-diphenyl-1H-indol-1-yl)-N,N-dimethyl-1,3,5-triazin-2-amine* **(3a)** White solid, yield 139.6 mg (71%), m.p: 175.8–176.5 °C; ^1^H NMR (400 MHz, CDCl_3_) δ 8.54 (s, 1H, CH), 8.53-8.51 (m, 1H, Ar-H), 7.35 (t, *J* = 7.6 Hz, 1H, Ar-H), 7.29-7.22 (m, 7H, Ar-H), 7.21-7.16 (m, 4H, Ar-H), 3.07 (s, 3H, N-CH_3_), 2.41 (s, 3H, N-CH_3_); ^13^C NMR (150 MHz, CDCl_3_) δ 166.3, 164.2, 162.9, 136.9, 135.9, 134.1, 133.9, 130.4, 130.2, 129.8, 128.2, 127.8, 126.8, 126.6, 124.3, 122.67, 122.1, 119.6, 115.0, 36.4, 35.6; HRMS (ESI) [M + H]^+^, calcd for C_25_H_22_N_5_: 392.1875, found: 392.1888.

*4--4-(7-methyl-2,3-diphenyl-1H-indol-1-yl)-N,N-dimethyl-1,3,5-triazin-2-amine* **(3b)** White solid, yield 125.8 mg (62%), m.p: 151.7–152.4 °C; ^1^H NMR (400 MHz, CDCl_3_) *δ* 8.53 (s, 1H, CH), 7.59 (d, *J* = 7.6 Hz, 1H, Ar-H), 7.36-7.29 (m, 4H, Ar-H), 7.26-7.19 (m, 6H, Ar-H), 7.18 (d, *J* = 7.6 Hz, 1H, Ar-H), 7.12 (d, *J* = 7.1 Hz, 1H, Ar-H), 3.20 (s, 3H, N-CH_3_), 2.88 (s, 3H, N-CH_3_), 2.33 (s, 3H, Ar-CH_3_); ^13^C NMR (100 MHz, CDCl_3_) *δ* 165.9, 164.5, 164.4, 137.2, 135.6, 134.3, 132.4, 131.1, 130.3, 129.4, 128.1, 127.7, 127.5, 126.5, 126.2, 122.7, 122.0, 119.4, 117.8, 36.7, 36.2, 20.6; HRMS (ESI) [M + H]^+^, calcd for C_26_H_24_N_5_: 406.2032, found: 406.2041.

*N,N-dimethyl-4-(6-methyl-2,3-diphenyl-1H-indol-1-yl)-1,3,5-triazin-2-amine* **(3c)** White solid, yield 121.1 mg (60%), m.p: 184.3–185.0 °C; ^1^H NMR (400 MHz, CDCl_3_) δ 8.57 (s, 1H, CH), 8.37 (s, 1H, Ar-H), 7.49 (d, *J* = 8.0 Hz, 1H, Ar-H), 7.31-7.27 (m, 1H, Ar-H), 7.26-7.19 (m, 4H, Ar-H), 7.20-7.16 (m, 5H, Ar-H), 7.08 (d, *J* = 8.0 Hz, 1H, Ar-H), 3.08 (s, 3H, N-CH_3_), 2.53 (s, 3H, N-CH_3_), 2.40 (s, 3H, Ar-CH_3_); ^13^C NMR (150 MHz, CDCl_3_) δ 166.2, 164.1, 162.9, 137.3, 135.3, 134.3, 134.2, 134.0, 130.3, 130.1, 128.1, 127.7, 127.6, 126.6, 126.4, 124.1, 122.0, 119.3, 115.1, 36.3, 35.6, 22.1; HRMS (ESI) [M + H]^+^, calcd for C_26_H_24_N_5_: 406.2032, found: 406.2047.

*N,N-dimethyl-4-(5-methyl-2,3-diphenyl-1H-indol-1-yl)-1,3,5-triazin-2-amine* **(3d)** White solid, yield 131.1 mg (65%), m.p: 213.4–214.7 °C; ^1^H NMR (400 MHz, CDCl_3_) *δ* 8.57 (s, 1H, CH), 8.47 (d, *J* = 8.5 Hz, 1H, Ar-H), 7.40 (s, 1H, Ar-H), 7.37-7.30 (m, 2H, Ar-H), 7.29-7.27 (m, 2H, Ar-H), 7.27-7.24 (m, 2H, Ar-H), 7.24-7.17 (m, 5H, Ar-H), 3.12 (s, 3H, N-CH_3_), 2.46 (s, 3H, N-CH_3_), 2.43 (s, 3H, Ar-CH_3_); ^13^C NMR (100 MHz, CDCl_3_) *δ* 165.8, 163.6, 162.8, 135.9, 135.2, 134.2, 133.9, 132.3, 130.4, 130.1, 130.0, 128.1, 127.8, 126.7, 126.5, 125.7, 122.3, 119.4, 114.9, 36.5, 35.6, 21.4; HRMS (ESI) [M + H]^+^, calcd for C_26_H_24_N_5_: 406.2032, found: 406.2042.

*4-(7-methoxy-2,3-diphenyl-1H-indol-1-yl)-N,N-dimethyl-1,3,5-triazin-2-amine* **(3e)** White solid, yield 126.8 mg (62%), m.p: 183.7–184.5 °C; ^1^H NMR (400 MHz, CDCl_3_) *δ* 8.53 (s, 1H, CH), 7.36 (d, *J* = 7.7 Hz, 2H, Ar-H), 7.33 (d, *J* = 6.9 Hz, 2H, Ar-H), 7.30-7.27 (m, 3H, Ar-H), 7.25-7.15 (m, 5H, Ar-H), 6.82 (d, *J* = 7.8 Hz, 1H, Ar-H), 3.84 (s, 3H, Ar-OCH_3_), 3.18 (s, 3H, N-CH_3_), 2.88 (s, 3H, N-CH_3_); ^13^C NMR (100 MHz, CDCl_3_) δ 165.4, 164.6, 164.1, 147.3, 137.2, 134.3, 132.1, 131.2, 130.7, 130.2, 128.1, 127.7, 127.5, 126.3, 126.1, 122.5, 119.3, 112.6, 105.8, 55.9, 36.6, 36.2. HRMS (ESI) [M + H]^+^, calcd for C_26_H_24_N_5_O: 422.1981, found: 422.1989.

*4-(5-methoxy-2,3-diphenyl-1H-indol-1-yl)-N,N-dimethyl-1,3,5-triazin-2-amine* **(3f)** White solid, yield 158.3 mg (75%), m.p: 178.6–180.2 °C; ^1^H NMR (600 MHz, CDCl_3_) *δ* 8.53 (s, 1H, CH), 8.49 (d, *J* = 9.1 Hz, 1H, Ar-H), 7.34-7.28 (m, 2H, Ar-H), 7.25-7.23 (m, 3H, Ar-H), 7.23-7.16 (m, 5H, Ar-H), 7.06 (d, *J* = 2.6 Hz, 1H, Ar-H), 6.98 (dd, *J* = 9.1, 2.6 Hz, 1H), 3.83 (s, 3H, Ar-OCH_3_), 3.08 (s, 3H, N-CH_3_), 2.40 (s, 3H, N-CH_3_); ^13^C NMR (150 MHz, CDCl_3_) *δ* 166.2, 164.1, 162.8, 156.1, 136.6, 134.2, 133.9, 131.7, 130.5, 130.3, 130.2, 128.2, 127.7, 126.7, 126.5, 122.1, 116.2, 113.2, 101.9, 55.8, 36.3, 35.6. HRMS (ESI) [M + H]^+^, calcd forC_26_H_24_N_5_O: 422.1981, found: 422.1977.

*4-(2,3-diphenyl-5-(trifluoromethoxy)-1H-indol-1-yl)-N,N-dimethyl-1,3,5-triazin-2-amine* **(3g)** White solid, yield 128.9 mg (54%), m.p: 175.8–176.5 °C; ^1^H NMR (400 MHz, CDCl_3_) *δ* 8.60 (d, *J* = 9.2 Hz, 1H, Ar-H), 8.58 (s, 1H, CH), 7.49 (s, 1H), 7.39-7.27 (m, 7H, Ar-H), 7.26-7.22 (m, 3H, Ar-H), 3.12 (s, 3H, N-CH), 2.44 (s, 3H, N-CH); ^13^C NMR (100 MHz, CDCl_3_) *δ* 166.3, 164.1, 162.8, 145.1, 137.7, 135.1, 133.6, 133.1, 130.3, 130.2, 130.1, 128.4, 127.9, 127.1, 126.9, 121.7, 120.74 (q, *J* = 256.0 Hz, CF_3_).117.6, 116.2, 112.0, 36.4, 35.6. HRMS (ESI) [M + H]^+^, calcd for C26H21N5OF3: 476.1698, found: 476.1716.

*4-(5-fluoro-2,3-diphenyl-1H-indol-1-yl)-N,N-dimethyl-1,3,5-triazin-2-amine* **(3h)** White solid, yield 100.9 mg (49%), m.p: 232.9–234.3 °C; ^1^H NMR (400 MHz, CDCl_3_) δ 8.57 (s, 1H, CH), 8.55 (dd, *J* = 9.1, 4.7 Hz, 1H, Ar-H), 7.37-7.32 (m, 1H, Ar-H), 7.32-7.27 (m, 3H, Ar-H), 7.26-7.24 (m, 3H, Ar-H), 7.22-7.19 (m, 3H, Ar-H), 7.10 (td, *J* = 9.1, 2.6 Hz, 1H, Ar-H), 3.11 (s, 3H, N-CH_3_), 2.42 (s, 3H, N-CH_3_); ^13^C NMR (100 MHz, CDCl_3_) δ 166.2, 164.0, 162.8, 159.5 (d, *J* = 238.4 Hz, C-F), 137.8, 133.9, 133.4, 133.2, 130.63 (d, *J* = 9.6 Hz, C-F), 130.2, 130.1, 128.3, 127.9, 127.0, 126.7, 121.9 (d, *J* = 4.0 Hz, C-F), 116.2 (d, *J* = 8.9 Hz, C-F), 111.9 (d, *J* = 24.9 Hz, C-F), 104.92 (d, *J* = 24.2 Hz, C-F), 36.4, 35.6. HRMS (ESI) [M + H]^+^, calcd for C_25_H_21_N_5_F: 410.1781, found: 410.1791.

*4-(5-chloro-2,3-diphenyl-1H-indol-1-yl)-N,N-dimethyl-1,3,5-triazin-2-amine* **(3i)** White solid, yield 112.5 mg (53%), m.p: 225.9–226.6 °C; ^1^H NMR (400 MHz, CDCl_3_) δ 8.58 (s, 1H, CH), 8.52 (dd, *J* = 8.9, 1.0 Hz, 1H, Ar-H), 7.60 (t, *J* = 1.0 Hz, 1H, Ar-H), 7.38-7.28 (m, 4H, Ar-H), 7.27-7.18 (m, 7H, Ar-H), 3.12 (s, 3H, N-CH_3_), 2.42 (s, 3H, N-CH_3_); ^13^C NMR (100 MHz, CDCl_3_) δ 166.2, 163.9, 162.7, 137.2, 135.3, 133.7, 133.1, 130.9, 130.2, 130.1, 128.3, 127.9, 127.1, 126.8, 124.3, 121.5, 119.1, 116.3, 36.4, 35.6; HRMS (ESI) [M + H]^+^, calcd for C_25_H_21_N_5_Cl: 426.1485, found: 426.1496.

*4-(4-(2,3-diphenyl-1H-indol-1-yl)-1,3,5-triazin-2-yl)morpholine* **(3j)** White solid, yield 164.9 mg (76%), m.p: 181.5–182.2 °C; ^1^H NMR (400 MHz, CDCl_3_) δ 8.64 (d, *J* = 8.4 Hz, 1H, Ar-H), 8.63 (s, 1H, CH), 7.65 (d, *J* = 7.8 Hz, 1H, Ar-H), 7.40 (d, *J* = 7.8 Hz, 1H, Ar-H), 7.35-7.15 (m, 11H, Ar-H), 3.77 (t, *J* = 4.1 Hz, 2H, O-CH_2_-), 3.65 (t, *J* = 4.1 Hz, 2H, O-CH_2_-), 3.31 (t, *J* = 4.1 Hz, 2H, N-CH_2_-), 2.85 (t, *J* = 4.1 Hz, 2H, N-CH_2_-); ^13^C NMR (100 MHz, CDCl_3_) *δ* 166.4, 163.1, 162.9, 136.9, 135.7, 134.4, 133.6, 130.3, 130.2, 129.9, 128.2, 127.8, 126.7, 126.7, 124.5, 122.9, 122.8, 119.7, 115.4, 66.6, 66.4, 43.7, 42.9; HRMS (ESI) [M + H]^+^, calcd for C_27_H_24_N_5_O: 434.1981, found: 434.1997.

*1-(4-(4-methylpiperazin-1-yl)-1,3,5-triazin-2-yl)-2,3-diphenyl-1H-indole* **(3k)** White solid, yield 154.1 mg (69%), m.p: 168.9–169.6 °C; ^1^H NMR (400 MHz, CDCl_3_) *δ* 8.62 (d, *J* = 8.4 Hz, 1H, Ar-H), 8.60 (s, 1H, CH), 7.64 (d, *J* = 7.8 Hz, 1H, Ar-H), 7.39 (t, *J* = 7.8 Hz, 1H, Ar-H), 7.34-7.30 (m, 2H, Ar-H), 7.30-7.20 (m, 9H, Ar-H), 3.80-3.75 (m, 2H, N-CH_2_-), 2.96-2.82 (m, 2H, N-CH_2_-), 2.38 (s, 3H, N-CH_3_), 2.140-2.35(m, 2H, N-CH_2_-), 2.10-2.01(m, 2H, N-CH_2_-); ^13^C NMR (100 MHz, CDCl_3_) *δ* 166.6, 163.2, 163.1, 136.9, 135.8, 134.3, 133.7, 130.3, 130.1, 129.8, 128.2, 127.8, 126.8, 126.6, 124.4, 122.8, 122.4, 119.6, 115.3, 54.6, 54.5, 46.0, 43.0, 42.3. HRMS (ESI) [M + H]^+^, calcd for C_28_H_27_N_6_: 447.2297, found: 447.2309.

*4-(2,3-di-p-tolyl-1H-indol-1-yl)-N,N-dimethyl-1,3,5-triazin-2-amine* **(3m)** White solid, yield 121.5 mg (58%), m.p: 137.3–138.1 °C; ^1^H NMR (400 MHz, CDCl_3_) *δ* 8.57 (s, 1H, CH), 8.54 (d, *J* = 8.3 Hz, 1H, Ar-H), 7.63 (d, *J* = 7.5 Hz, 1H, Ar-H), 7.40-7.34 (m, 1H, Ar-H), 7.26 (t, *J* = 7.5 Hz, 1H, Ar-H), 7.19 (d, *J* = 8.1 Hz, 2H, Ar-H), 7.14 (d, *J* = 8.1 Hz, 2H, Ar-H), 7.11 (d, *J* = 8.0 Hz, 2H, Ar-H), 7.05 (d, *J* = 8.0 Hz, 2H, Ar-H), 3.14 (s, 3H, N-CH_3_), 2.51 (s, 2H, N-CH_3_), 2.38 (s, 2H, Ar-CH_3_), 2.34 (s, 3H Ar-CH_3_); ^13^C NMR (100 MHz, CDCl_3_) δ 165.9, 163.8, 162.9, 136.8, 136.4, 136.0, 135.8, 131.1, 130.9, 130.2, 130.0, 129.9, 128.9, 128.5, 124.1, 122.6, 121.8, 119.6, 114.9, 36.5, 35.6, 21.2. HRMS (ESI) [M + H]^+^, calcd for C_27_H_26_N_5_: 420.2188, found: 420.2199.

*4-(2,3-bis(4-ethylphenyl)-1H-indol-1-yl)-N,N-dimethyl-1,3,5-triazin-2-amine* **(3n)** White solid, yield 127.5 mg (57%), m.p: 192.4–193.1 °C; ^1^H NMR (400 MHz, CDCl_3_) δ 8.59 (s, 1H, CH), 8.56 (d, *J* = 8.3 Hz, 1H, Ar-H), 7.65 (d, *J* = 7.5 Hz, 1H, Ar-H), 7.36 (td, *J* = 7.5, 1.12 Hz, 1H, Ar-H), 7.28-7.24(m, 1H, Ar-H), 7.22 (d, *J* = 8.1 Hz, 2H, Ar-H), 7.16 (d, *J* = 7.6 Hz, 2H, Ar-H), 7.14 (d, *J* = 7.6 Hz, 2H, Ar-H), 7.08 (d, *J* = 8.1 Hz, 2H, Ar-H), 3.13 (s, 3H, N-CH_3_), 2.73-2.60 (m, 4H, Ar-CH_2_-), 2.45 (s, 3H, N-CH_3_), 1.28 (t, *J* = 7.6 Hz, 6H, -CH_3_),1.24 (t, *J* = 7.6 Hz, 6H, -CH_3_); ^13^C NMR (100 MHz, CDCl_3_) *δ* 165.9, 163.8, 162.9, 142.8, 142.3, 136.9, 135.9, 131.4, 131.1, 130.2, 130.1, 130.0, 127.6, 127.3, 124.1, 122.6, 121. 8, 119.7, 114.9, 36.4, 35.6, 28.7, 28.5, 15.8, 15.3; HRMS (ESI) [M + H]^+^, calcd for C_29_H_30_N_5_: 448.2501, found: 448.2507.

*4-(2,3-bis(4-chlorophenyl)-1H-indol-1-yl)-N,N-dimethyl-1,3,5-triazin-2-amine* **(3o)** White solid, yield 154.2 mg (67%), m.p: 173.9–174.5 °C; ^1^H NMR (400 MHz, CDCl_3_) δ 8.58 (s, 1H, CH), 8.57 (d, *J* = 7.8 Hz, 1H, Ar-H), 7.59 (dd, *J* = 7.8, 0.7 Hz, 1H, Ar-H), 7.40 (t, *J* = 7.8 Hz, 1H, Ar-H), 7.34-7.29 (m, 3H, Ar-H), 7.26 (d, *J* = 8.5 Hz, 2H, Ar-H), 7.23-7.18 (m, 2H, Ar-H), 7.14 (d, *J* = 8.5 Hz, 2H, Ar-H), 3.16 (s, 3H, N-CH_3_), 2.55 (s, 3H, N-CH_3_); ^13^C NMR (100 MHz, CDCl_3_) *δ* 165.9, 163.6, 162.7, 136.9, 134.7, 133.1, 132.7, 132.4, 131.9, 131.5, 131.4, 129.3, 128.6, 128.2, 124.7, 123.0, 121.5, 119.4, 115.3, 36.5, 35.6; HRMS (ESI) [M + H]^+^, calcd for C_25_H_20_N_5_Cl_2_: 460.1096, found: 460.1104.

*4-(2,3-bis(4-fluorophenyl)-1H-indol-1-yl)-N,N-dimethyl-1,3,5-triazin-2-amine* **(3p)** White solid, yield 149.6 mg (70%), m.p: 175.8–176.5 °C; ^1^H NMR (400 MHz, CDCl_3_) *δ* 8.60-8.53 (m, 2H, Ar-H, CH), 7.58 (d, *J* = 7.7 Hz, 1H, Ar-H), 7.45-7.35 (m, 1H, Ar-H), 7.32-7.29 (m, 1H, Ar-H), 7.26-7.20 (m, 2H, Ar-H), 7.20-7.15 (m, 2H, Ar-H), 7.07-7.01 (m, 2H, Ar-H), 7.00-6.94 (m, 2H, Ar-H) 3.16 (s, 3H, N-CH_3_), 2.57 (s, 3H, N-CH_3_); ^13^C NMR (100 MHz, CDCl_3_) *δ* 166.0, 163.8, 162.8, 161.9 (d, *J* = 246.9 Hz, C-F); 161.7 (d, *J* = 246.0 Hz, C-F); 136.7, 134.9, 131.8, 131.7, 130.0 (d, *J* = 3.5 Hz, C-F), 129.6, 129.5 (d, *J* = 3.3 Hz, C-F), 124.5, 122.9, 121.4, 119.4, 115.3 (d, *J* = 21.4 Hz, C-F), 115.2, 114.9 (d, *J* = 21.6 Hz, C-F), 36.5, 35.7; HRMS (ESI) [M + H]^+^, calcd for C_25_H_20_N_5_F_2_: 428.1687, found: 428.1694.

*4-(2,3-di(thiophen-2-yl)-1H-indol-1-yl)-N,N-dimethyl-1,3,5-triazin-2-amine* **(3q)** White solid, yield 90.6 mg (45%), m.p: 186.1–186.9 °C; ^1^H NMR (400 MHz, CDCl_3_) *δ* 8.59 (s, 1H, CH), 8.51 (d, *J* = 8.4 Hz, 1H, Ar-H), 7.85 (d, *J* = 7.8 Hz, 1H, Ar-H), 7.44-7.37 (m, 2H, Ar-H), 7.36-7.33 (m, 1H, thiazole-H), 7.31 (dd, *J* = 5.1, 1.0 Hz, 1H, thiazole-H), 7.13 (dd, *J* = 3.6, 1.0 Hz, 1H, thiazole-H), 7.10-7.07 (m, 1H, thiazole-H), 7.06 (dd, *J* = 3.6, 1.3 Hz, 1H, thiazole-H), 7.03 (dd, *J* = 5.1, 3.5 Hz, 1H, thiazole-H), 3.18 (s, 3H, N-CH_3_), 2.72 (s, 3H, N-CH_3_); ^13^C NMR (100 MHz, CDCl_3_) δ 166.2, 164.3, 162.7, 136.7, 134.7, 134.4, 129.5, 129.0, 128.8, 127.1, 126.8, 126.7, 125.4, 124.9, 122.9, 120.0, 117.5, 115.0, 36.5, 35.9. HRMS (ESI) [M + H]^+^, calcd for C_21_H_18_N_5_S_2_: 404.1004, found: 404.1015.

*4-(2-(4-methoxyphenyl)-3-(4-propylphenyl)-1H-indol-1-yl)-N,N-dimethyl-1,3,5-triazin-2-amine* **(3r,** 2:1**)** White solid, yield 127.5 mg (55%), m.p: 177.1–178.6 °C; ^1^H NMR (400 MHz, CDCl_3_) *δ* 8.59 (s, 1H, CH), 8.57 (d, *J* = 8.4 Hz, 1H, Ar-H), 7.62 (d, *J* = 7.8 Hz, 1H, Ar-H), 7.37 (t, *J* = 7.8 Hz, 1H, Ar-H), 7.30-7.25 (m, 1H, Ar-H), 7.23-7.18 (m, 2H, Ar-H), 7.12 (d, *J* = 7.5 Hz, 2H, Ar-H), 7.06 (d, *J* = 7.5 Hz, 2H, Ar-H), 6.87 (d, *J* = 8.4 Hz, 2H, Ar-H), 3.84 (s, 3H, O-CH_3_), 3.14 (s, 3H, N-CH_3_), 2.58 (t, *J* = 7.6 Hz, 2H, Ar-CH_2_-), 2.45 (s, 3H, N-CH_3_), 1.69-1.55 (m, 2H, -CH_2_-), 0.96 (t, *J* = 7.4 Hz, 3H,-CH_3_); ^13^C NMR (100 MHz, CDCl_3_) *δ* 165.4, 162.7, 158.3, 141.3, 136.9, 135.8, 131.4, 131.4, 130.1, 129.9, 127.9, 126.1, 124.2, 122.7, 122.1, 121.9, 119.6, 115.2, 113.6, 55.2, 37.8, 36.6, 35.8, 24.6, 13.8. HRMS (ESI) [M + H]^+^, calcd for C_29_H_30_N_5_O: 464.2450, found: 464.2462.

## 4. Conclusions

In summary, we developed a novel ruthenium-catalyzed oxidative annulation of alkynes with N-phenyl triazines to prepare N-(2-triazine) indoles in moderate to good yields. These studies indicated that the triazine ring could act as an efficient directing group for the synthesis of bioactive indole compounds. Detailed mechanistic studies and work on substrate expansions are in progress.

## Figures and Tables

**Table 1:**
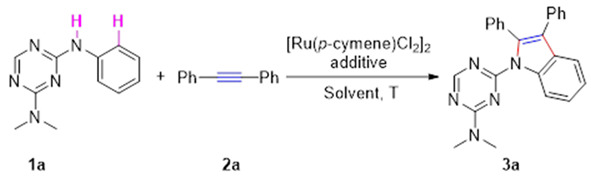
Annulation reaction condition optimization *^a^*.

Entry	Additive (%)	Solvent	Temp. (°C)	Yield (%) *^b^*
1	Cu(OAc)_2_ (10)	DMF	120	55
2	Cu (NO_3_)_2_(10)	DMF	120	-
3	CuCl_2_ (10)	DMF	120	10
4	CuBr_2_ (10)	DMF	120	22
5	Cu(CF_3_SO_3_)_2_ (10)	DMF	120	44
6	NaOAc (10)	DMF	120	0
7	CsOAc (10)	DMF	120	0
8	Cu(OAc)_2_ (10)	DMF	120	50 *^c^*
9	-	DMF	120	0
10	Cu(OAc)_2_ (20)	DMF	120	46
11	Cu(OAc)_2_ (5)	DMF	120	19
12	Cu(OAc)_2_ (10)	DMF	100	63
13	Cu(OAc)_2_ (10)	DMF	80	52
14	Cu(OAc)_2_ (10)	DMF	140	32
**15** *^d^*	**Cu(OAc)_2_ (10)**	**DMF**	**100**	**71**
16 *^e^*	Cu(OAc)_2_ (10)	DMF	100	58
17 *^f^*	Cu(OAc)_2_ (10)	DMF	100	42
18 *^g^*	Cu(OAc)_2_ (10)	DMF	100	32
19 *^h^*	Cu(OAc)_2_ (10)	DMF	100	56
20	Cu(OAc)_2_ (10)	MeCN	100	13
21	Cu(OAc)_2_ (10)	H_2_O	100	0
22	Cu(OAc)_2_ (10)	MeOH	100	32
23	Cu(OAc)_2_ (10)	PEG-400	100	46
24 *^i^*	Cu(OAc)_2_ (10)	DMF	100	0 (Pd(OAc)_2_)
25 *^j^*	Cu(OAc)_2_ (10)	DMF	100	0 (PdCl_2_)
26 *^k^*	Cu(OAc)_2_ (10)	DMF	100	64 (Ru 10%)
27 *^l^*	Cu(OAc)_2_ (10)	DMF	100	22 (Ru 2%)

Reaction conditions: *^a^* **1a** (1 mmol), **2a** (1.0 mmol), [Ru(p-cymene) Cl_2_]_2_ (5 mol%), additive (10 mol%), and solvent (3 mL), 12 h. *^b^* Isolated yield. *^c^* Additional 10% of KBF_4_. *^d^* 1.5 mmol of **2a**. *^e^* 2.0 mmol of **2a**. *^f^* 0.8 mmol of **1a**. *^f^* 6 h. *^g^* 24 h. *^h^* 24 h. *^i^* Pd(OAc)_2_ instead of [Ru(p-cymene) Cl_2_]_2_. *^j^* PdCl_2_ instead of [Ru(p-cymene) Cl_2_]_2_. *^k^* 10 mol% of [Ru(p-cymene) Cl_2_]_2_. *^l^* 2 mol% of [Ru(p-cymene) Cl_2_]_2_.

## Data Availability

Data are contained within the article and Supplementary Materials.

## Supplementary Materials

The following supporting information can be downloaded at: https://www.mdpi.com/article/10.3390/molecules28093676/s1, Figure S1, ^1^H NMR spectrum of *4-(2,3-diphenyl-1H-indol-1-yl)-N,N-dimethyl-1,3,5-triazin-2-amine* (**3a**); Figure S2, ^13^C NMR spectrum of *4-(2,3-diphenyl-1H-indol-1-yl)-N,N-dimethyl-1,3,5-triazin-2-amine* (**3a**); Figure S3, ^1^H NMR spectrum of *4--4-(7-methyl-2,3-diphenyl-1H-indol-1-yl)-N,N-dimethyl-1,3,5-triazin-2-amine* **(3b)**; Figure S4, ^13^C NMR spectrum of *4--4-(7-methyl-2,3-diphenyl-1H-indol-1-yl)-N,N-dimethyl-1,3,5-triazin-2-amine* **(3b)**; Figure S5, ^1^H NMR spectrum of *N,N-dimethyl-4-(6-methyl-2,3-diphenyl-1H-indol-1-yl)-1,3,5-triazin-2-amine* **(3c)**; Figure S6, *N,N-dimethyl-4-(6-methyl-2,3-diphenyl-1H-indol-1-yl)-1,3,5-triazin-2-amine* **(3c)**; Figure S7, ^1^H NMR spectrum of *N,N-dimethyl-4-(5-methyl-2,3-diphenyl-1H-indol-1-yl)-1,3,5-triazin-2-amine* **(3d)**; Figure S8, ^13^C NMR spectrum of *N,N-dimethyl-4-(5-methyl-2,3-diphenyl-1H-indol-1-yl)-1,3,5-triazin-2-amine* **(3d)**; Figure S9, ^1^H NMR spectrum of *4-(7-methoxy-2,3-diphenyl-1H-indol-1-yl)-N,N-dimethyl-1,3,5-triazin-2-amine* **(3e)**; Figure S10, ^13^C NMR spectrum of *4-(7-methoxy-2,3-diphenyl-1H-indol-1-yl)-N,N-dimethyl-1,3,5-triazin-2-amine* **(3e)**; Figure S11, ^1^H NMR spectrum of *4-(5-methoxy-2,3-diphenyl-1H-indol-1-yl)-N,N-dimethyl-1,3,5-triazin-2-amine* **(3f)**; Figure S12, ^13^C NMR spectrum of *4-(5-methoxy-2,3-diphenyl-1H-indol-1-yl)-N,N-dimethyl-1,3,5-triazin-2-amine* **(3f)**; Figure S13, ^1^H NMR spectrum of *4-(2,3-diphenyl-5-(trifluoromethoxy)-1H-indol-1-yl)-N,N-dimethyl-1,3,5-triazin-2-amine* **(3g)**; Figure S14, ^13^C NMR spectrum of *4-(2,3-diphenyl-5-(trifluoromethoxy)-1H-indol-1-yl)-N,N-dimethyl-1,3,5-triazin-2-amine* **(3g)**; Figure S15, ^1^H NMR spectrum of *4-(5-fluoro-2,3-diphenyl-1H-indol-1-yl)-N,N-dimethyl-1,3,5-triazin-2-amine* **(3h)**; Figure S16, ^13^C NMR spectrum of *4-(5-fluoro-2,3-diphenyl-1H-indol-1-yl)-N,N-dimethyl-1,3,5-triazin-2-amine* **(3h)**; Figure S17, ^1^H NMR spectrum of *4-(5-chloro-2,3-diphenyl-1H-indol-1-yl)-N,N-dimethyl-1,3,5-triazin-2-amine* **(3i)**; Figure S18, ^13^C NMR spectrum of *4-(5-chloro-2,3-diphenyl-1H-indol-1-yl)-N,N-dimethyl-1,3,5-triazin-2-amine* **(3i)**; Figure S19, ^1^H NMR spectrum of *4-(4-(2,3-diphenyl-1H-indol-1-yl)-1,3,5-triazin-2-yl)morpholine* **(3j)**; Figure S20, ^13^C NMR spectrum of *4-(4-(2,3-diphenyl-1H-indol-1-yl)-1,3,5-triazin-2-yl)morpholine* **(3j)**; Figure S21, ^1^H NMR spectrum of *1-(4-(4-methylpiperazin-1-yl)-1,3,5-triazin-2-yl)-2,3-diphenyl-1H-indole* **(3k)**; Figure S22, ^13^C NMR spectrum of *1-(4-(4-methylpiperazin-1-yl)-1,3,5-triazin-2-yl)-2,3-diphenyl-1H-indole* **(3k)**; Figure S23, ^1^H NMR spectrum of *4-(2,3-di-p-tolyl-1H-indol-1-yl)-N,N-dimethyl-1,3,5-triazin-2-amine* **(3m)**; Figure S24, ^13^C NMR spectrum of *4-(2,3-di-p-tolyl-1H-indol-1-yl)-N,N-dimethyl-1,3,5-triazin-2-amine* **(3m)**; Figure S25, ^1^H NMR spectrum of *4-(2,3-bis(4-ethylphenyl)-1H-indol-1-yl)-N,N-dimethyl-1,3,5-triazin-2-amine* **(3n)**; Figure S26, ^13^C NMR spectrum of *4-(2,3-bis(4-ethylphenyl)-1H-indol-1-yl)-N,N-dimethyl-1,3,5-triazin-2-amine* **(3n)**; Figure S27, ^1^H NMR spectrum of *4-(2,3-bis(4-chlorophenyl)-1H-indol-1-yl)-N,N-dimethyl-1,3,5-triazin-2-amine* **(3o)**; Figure S28, ^13^C NMR spectrum of *4-(2,3-bis(4-chlorophenyl)-1H-indol-1-yl)-N,N-dimethyl-1,3,5-triazin-2-amine* **(3o)**; Figure S29, ^1^H NMR spectrum of *4-(2,3-bis(4-fluorophenyl)-1H-indol-1-yl)-N,N-dimethyl-1,3,5-triazin-2-amine* **(3p)**; Figure S30, ^13^C NMR spectrum of *4-(2,3-bis(4-fluorophenyl)-1H-indol-1-yl)-N,N-dimethyl-1,3,5-triazin-2-amine* **(3p)**; Figure S31, ^1^H NMR spectrum of *4-(2,3-di(thiophen-2-yl)-1H-indol-1-yl)-N,N-dimethyl-1,3,5-triazin-2-amine* **(3q)**; Figure S32, ^13^C NMR spectrum of *4-(2,3-di(thiophen-2-yl)-1H-indol-1-yl)-N,N-dimethyl-1,3,5-triazin-2-amine* **(3q)**; Figure S33, ^1^H NMR spectrum of *4-(2-(4-methoxyphenyl)-3-(4-propylphenyl)-1H-indol-1-yl)-N,N-dimethyl-1,3,5-triazin-2-amine* **(3r** and **3r’)**; Figure S34, ^13^C NMR spectrum of *4-(2-(4-methoxyphenyl)-3-(4-propylphenyl)-1H-indol-1-yl)-N,N-dimethyl-1,3,5-triazin-2-amine* **(3r** and **3r’)**; Figure S35, ^1^H NMR spectrum of *N^2^,N^2^-dimethyl-N^4^-phenyl-1,3,5-triazine-2,4-diamine* **(1a)**; Figure S36, ^13^C NMR spectrum of *N^2^,N^2^-dimethyl-N^4^-phenyl-1,3,5-triazine-2,4-diamine* **(1a)**; Figure S37, ^1^H NMR spectrum of *4-morpholino-N-phenyl-1,3,5-triazin-2-amine* **(1j)**; Figure S38, ^13^C NMR spectrum of *4-morpholino-N-phenyl-1,3,5-triazin-2-amine* **(1j)**; Figure S39, ^1^H NMR spectrum of *4-(4-methylpiperazin-1-yl)-N-phenyl-1,3,5-triazin-2-amine* **(1k)**; Figure S40, ^13^C NMR spectrum of *4-(4-methylpiperazin-1-yl)-N-phenyl-1,3,5-triazin-2-amine* **(1k)**; Figure S41, ^1^H NMR spectrum of *N^2^,N^2^,6-trimethyl-N^4^-phenyl-1,3,5-triazine-2,4-diamine* **(1l)**; Figure S42, ^13^C NMR spectrum of *N^2^,N^2^,6-trimethyl-N^4^-phenyl-1,3,5-triazine-2,4-diamine* **(1l)**; Figure S43: The analysis of relative yield of the isomers **3r** and **3r’** by ^1^H NMR.

## Sample Availability

Samples of all the compounds are available from the authors.
