# Supplementary material for: Ruthenium-Catalyzed Oxidative Synthesis of N-(2-triazine)indoles by C-H Activation
Source: Molecules. 2023 Apr 24;28(9):3676. doi: 10.3390/molecules28093676 (PMC10179826; doi:10.3390/molecules28093676)
Supplement: Supplementary file 1 [file molecules-28-03676-s001.zip › molecules-2343279-supplementary.pdf]

Ruthenium Catalyzed Oxidative Synthesis of *N*-(2-triazine)indoles by C-H Activation

Ming Zeng *, Jia Qi Chen, Feng Ye Li, Hao Jie Li, Lan Zhao, Deng Zhao Jiang, Jun Dai and Wen Bo Liu

School of Pharmacy and Life Science, Jiujiang University, Jiujiang 332005, China

* Correspondence: zengming@zjut.edu.cn

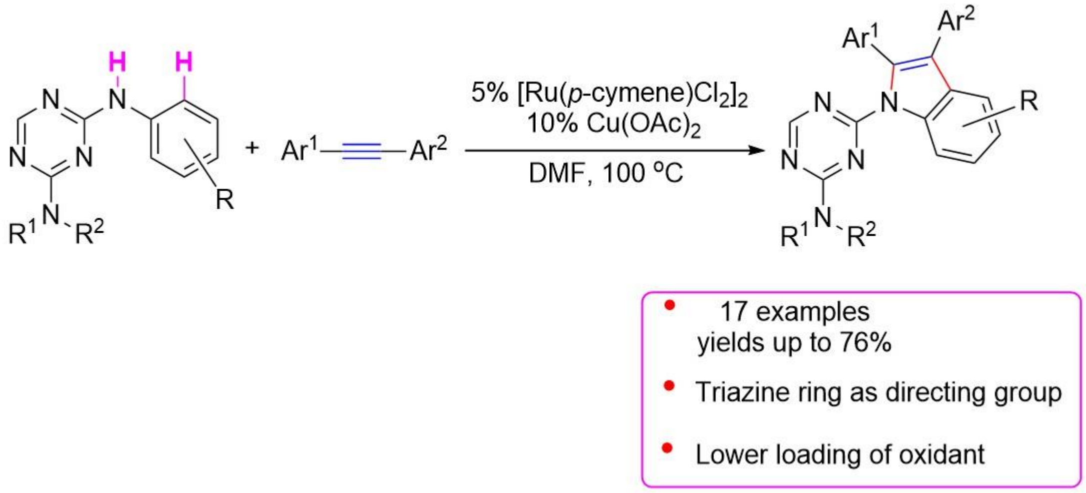

1,3,5 triazines, especially indole functionalized triazine derivatives, exhibit excellent activities, such as anti-tumor, antibacterial, and anti-inflammatory activities. Traditional methods for the synthesis of N-(2-triazine) indoles suffer from unstable materials and tedious operations. Transition-metal-catalyzed C-C/C-N coupling provides a powerful protocol for the synthesis of indoles by the C-H activation strategy. Here, we report the efficient ruthenium-catalyzed oxidative synthesis of *N*-(2-triazine) indoles by C-H activation from alkynes and various substituted triazine derivatives in a moderate to good yield, and all of the N-(2-triazine) indoles were characterized by ^{1}H NMR, ^{13}C NMR, and HRMS. This protocol can apply to the gram-scale synthesis of the N-(2-triazine) indole in a moderate yield. Moreover, the reaction is proposed to be performed via a six-membered ruthenacycle (II) intermediate, which suggests that the triazine ring could offer chelation assistance for the formation of N-(2-triazine) indoles.

The ^{1}H and ^{13}C spectra of compounds

Index	Content	Pages
Figure S1	^{1}H spectra of **3a**	S4
Figure S2	^{13}C spectra of **3a**	S5
Figure S3	^{1}H spectra of **3b**	S6
Figure S4	^{13}C spectra of **3b**	S7
Figure S5	^{1}H spectra of **3c**	S8
Figure S6	^{13}C spectra of **3c**	S9
Figure S7	^{1}H spectra of **3d**	S10
Figure S8	^{13}C spectra of **3d**	S11
Figure S9	^{1}H spectra of **3e**	S12
Figure S10	^{13}C spectra of **3e**	S13
Figure S11	^{1}H spectra of **3f**	S14
Figure S12	^{13}C spectra of **3f**	S15
Figure S13	^{1}H spectra of **3g**	S16
Figure S14	^{13}C spectra of **3g**	S17
Figure S15	^{1}H spectra of **3h**	S18
Figure S16	^{13}C spectra of **3h**	S19
Figure S17	^{13}C spectra of **3i**	S20
Figure S18	^{1}H spectra of **3i**	S21
Figure S19	^{13}C spectra of **3j**	S22
Figure S20	^{13}C spectra of **3j**	S23
Figure S21	^{1}H spectra of **3k**	S24
Figure S22	^{13}C spectra of **3k**	S25
Figure S23	^{1}H spectra of **3m**	S26
Figure S24	^{13}C spectra of **3m**	S27
Figure S25	^{1}H spectra of **3n**	S28
Figure S26	^{13}C spectra of **3n**	S29
Figure S27	^{1}H spectra of **3o**	S30
Figure S28	^{13}C spectra of **3o**	S31
Figure S29	^{13}C spectra of **3p**	S32
Figure S30	^{1}H spectra of **3p**	S33
Figure S31	^{13}C spectra of **3q**	S34
Figure S32	^{13}C spectra of **3q**	S35
Figure S33	^{1}H spectra of **3r and 3r’**	S36
Figure S34	^{13}C spectra of **3r and 3r’**	S37
Figure S35	^{1}H spectra of **1a**	S38
Figure S36	^{13}C spectra of **1a**	S39
Figure S37	^{1}H spectra of **1j**	S40
Figure S38	^{13}C spectra of **1j**	S41
Figure S39	^{1}H spectra of **1k**	S42
Figure S40	^{13}C spectra of **1k**	S43
Figure S41	^{1}H spectra of **1l**	S44

Figure S42	^{13}C spectra of **1l**	S45
Figure S43	The analysis of relative yield of the isomers **3r** and **3r’** by 1H NMR.	S46

PROTON_01
HT201120

8.5428
8.5256
7.6199
7.6013
7.3637
7.3460
7.3259
7.2870
7.2526
7.2408
7.1902
3.0716
2.4062

Figure S1, ^{1}H NMR spectrum of *4-(2,3-diphenyl-1H-indol-1-yl)-N,N-dimethyl-1,3,5-triazin-2-amine* (**3a**) in CDCl3

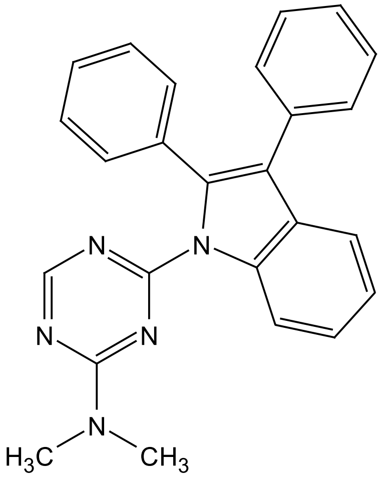

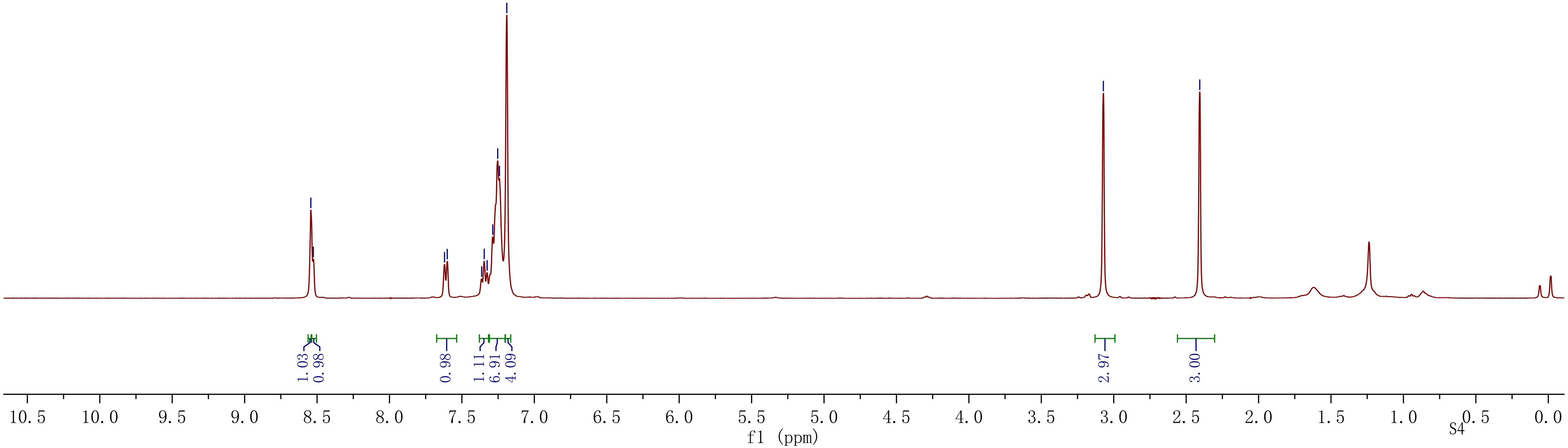

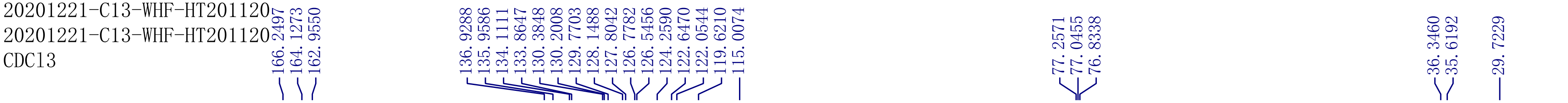

Figure S2, ^{13}C NMR spectrum of *4-(2,3-diphenyl-1H-indol-1-yl)-N,N-dimethyl-1,3,5-triazin-2-amine* (**3a**) in CDCl3

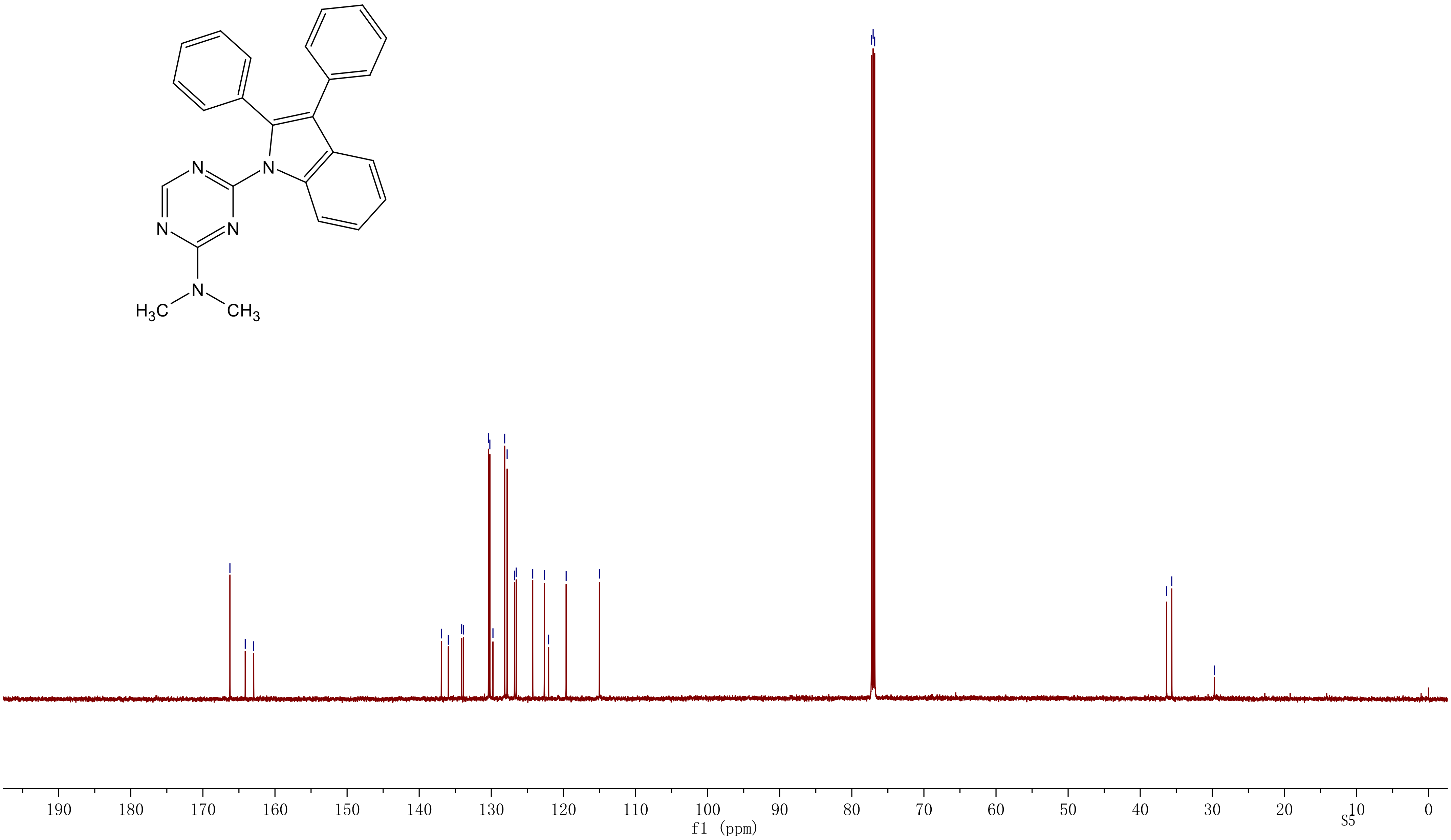

20220112zm
F210904

Figure S3, ¹H NMR spectrum of *4--4-(7-methyl-2,3-diphenyl-1H-indol-1-yl)-N,N-dimethyl-1,3,5-triazin-2-amine* **(3b)** in CDCl3

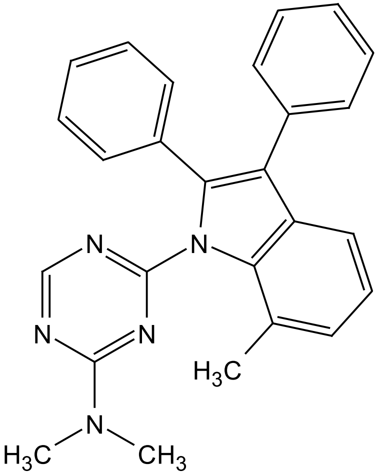

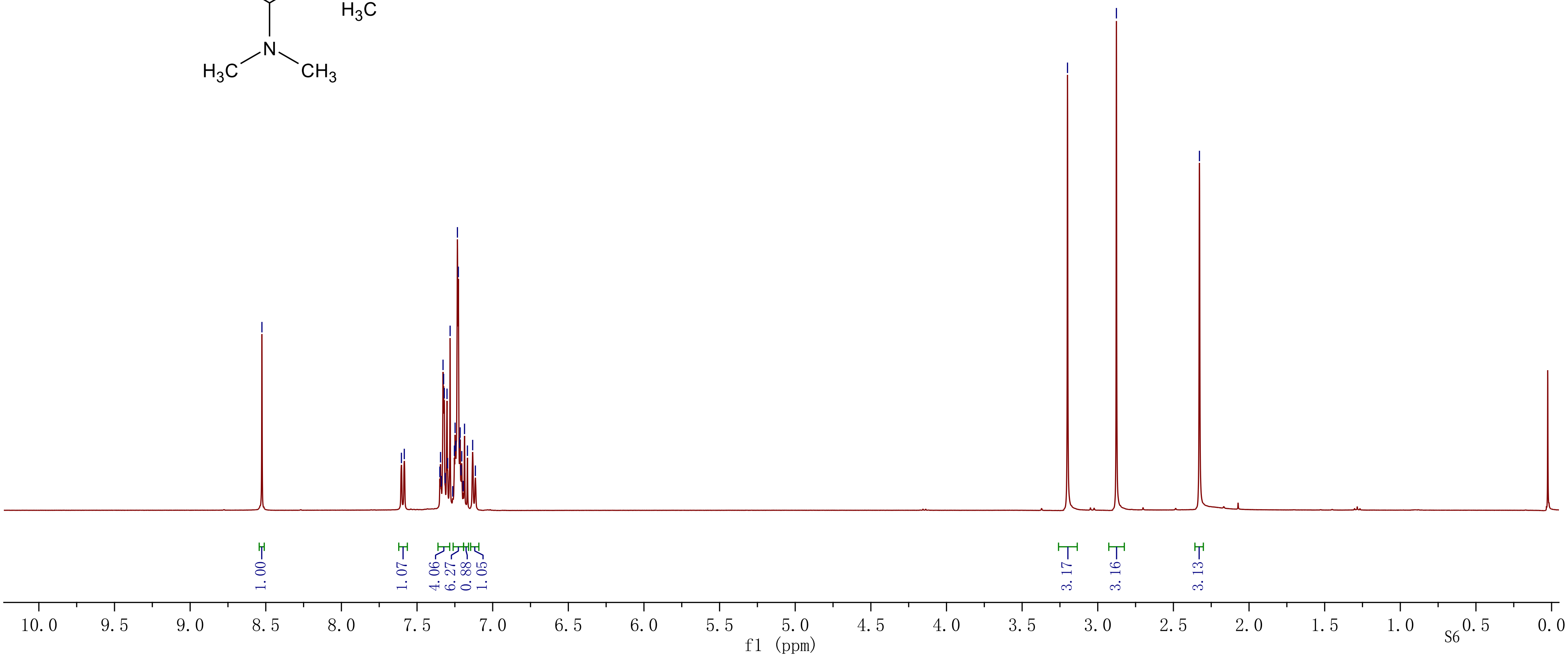

Figure S4, ^{13}C NMR spectrum of *4--4-(7-methyl-2,3-diphenyl-1H-indol-1-yl)-N,N-dimethyl-1,3,5-triazin-2-amine* **(3b)** in CDCl3

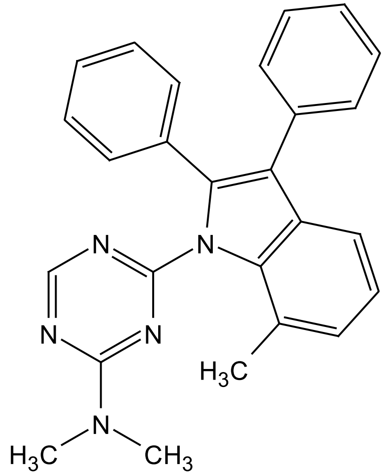

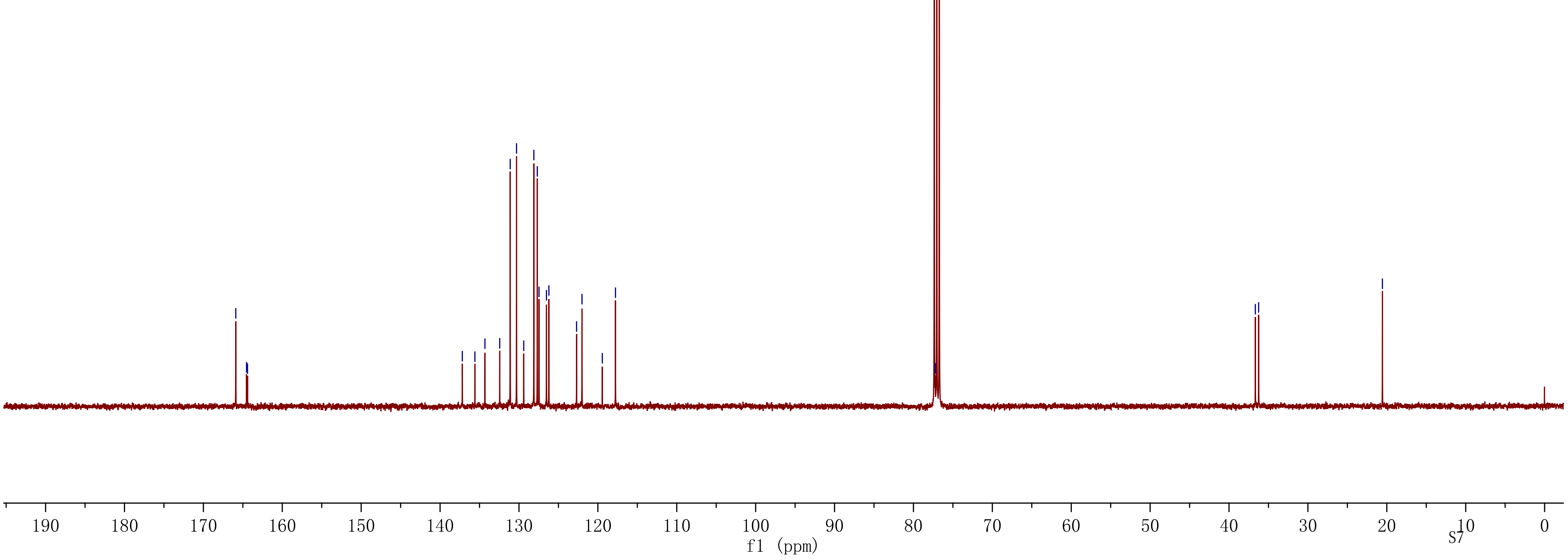

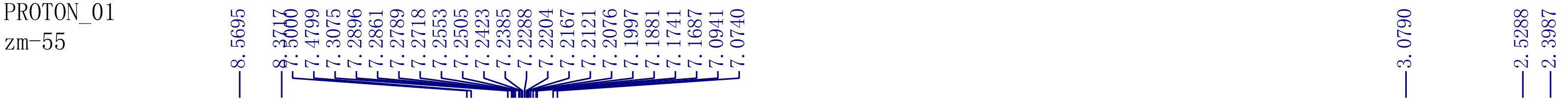

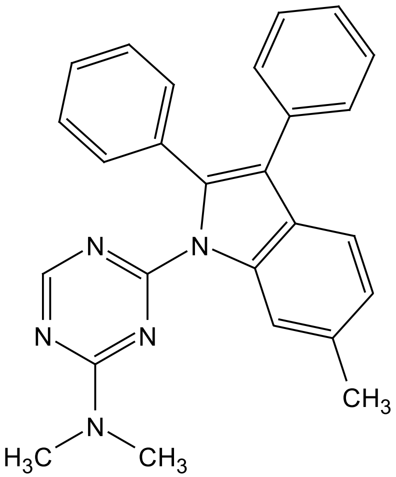

Figure S5, ^{1}H NMR spectrum of *N,N-dimethyl-4-(6-methyl-2,3-diphenyl-1H-indol-1-yl)-1,3,5-triazin-2-amine* **(3c)** in CDCl3

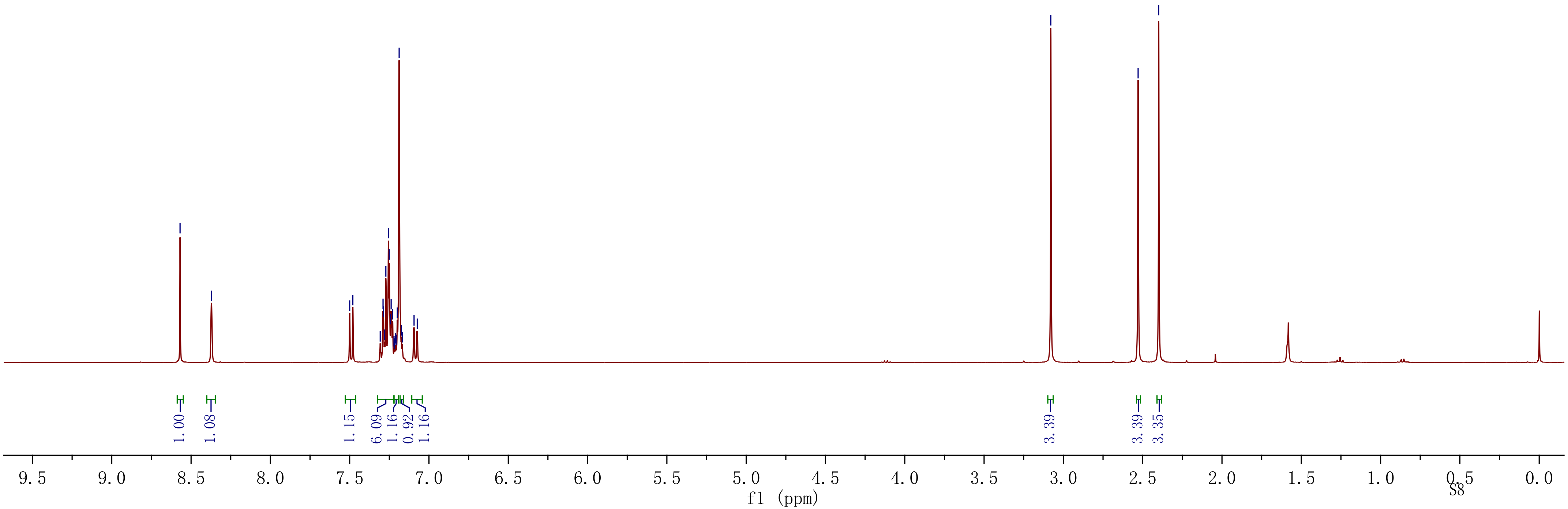

20210924-C13-WHF-55
20210924-C13-WHF-55
CDCl3

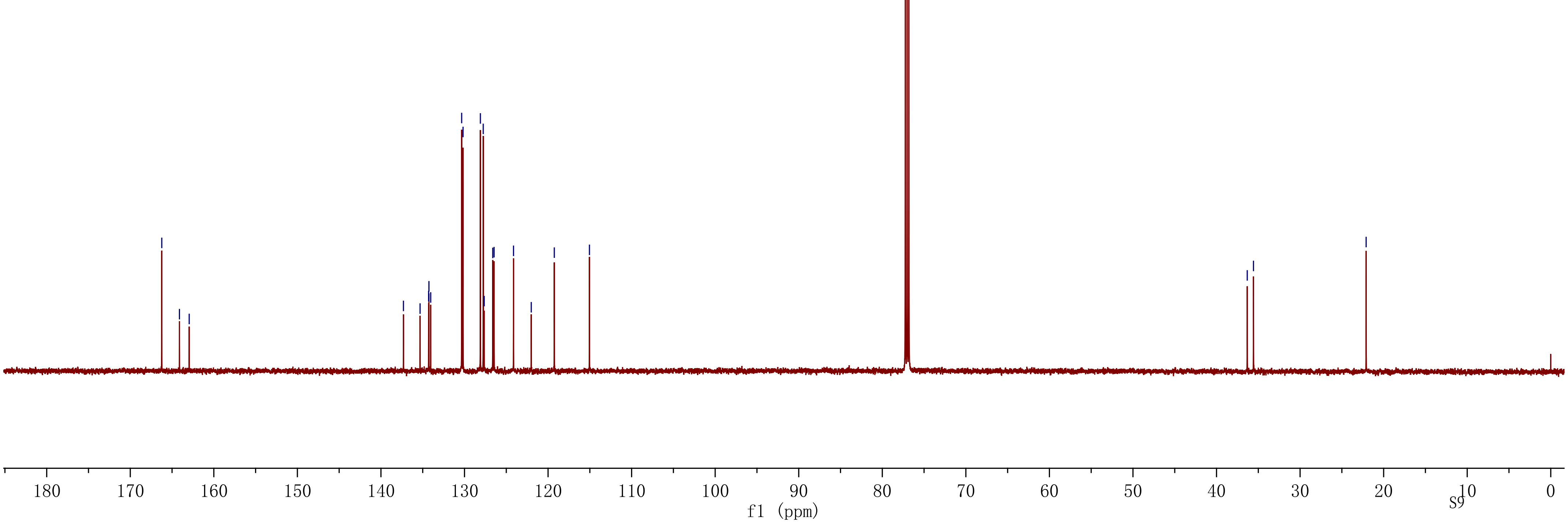

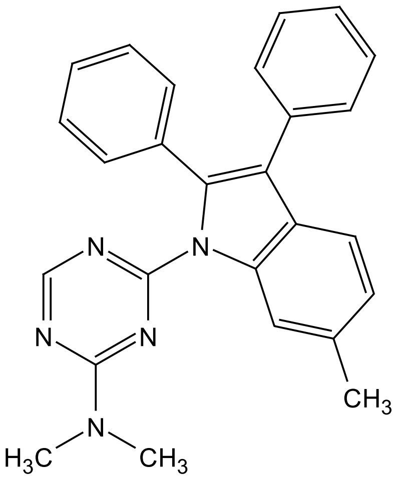

Figure S6, ^{13}C NMR spectrum of *N,N-dimethyl-4-(6-methyl-2,3-diphenyl-1H-indol-1-yl)-1,3,5-triazin-2-amine* **(3c)** in CDCl3

20220112zm
F210607

8.5749, 8.4821, 8.4608, 7.4040, 7.3518, 7.3485, 7.3446, 7.3335, 7.3298, 7.3272, 7.3226, 7.3129, 7.2867, 7.2806, 7.2771, 7.2707, 7.2634, 7.2596, 7.2561, 7.2503, 7.2466, 7.2407, 7.2331, 7.2226, 7.2203, 7.2129, 7.2065, 7.2001, 7.1945, 7.1910, 3.1237, 2.4604, 2.4321

Figure S7, ^{1}H NMR spectrum of *N,N-dimethyl-4-(5-methyl-2,3-diphenyl-1H-indol-1-yl)-1,3,5-triazin-2-amine* **(3d)** in CDCl3

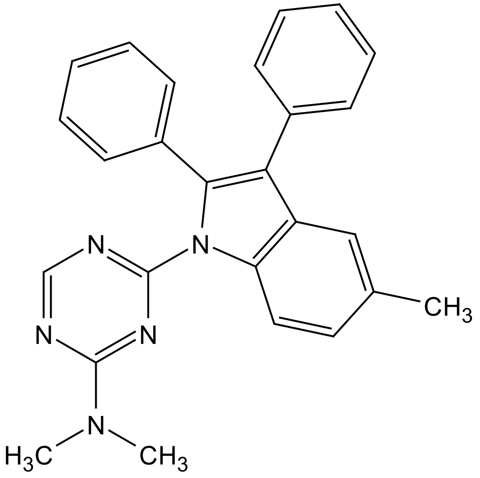

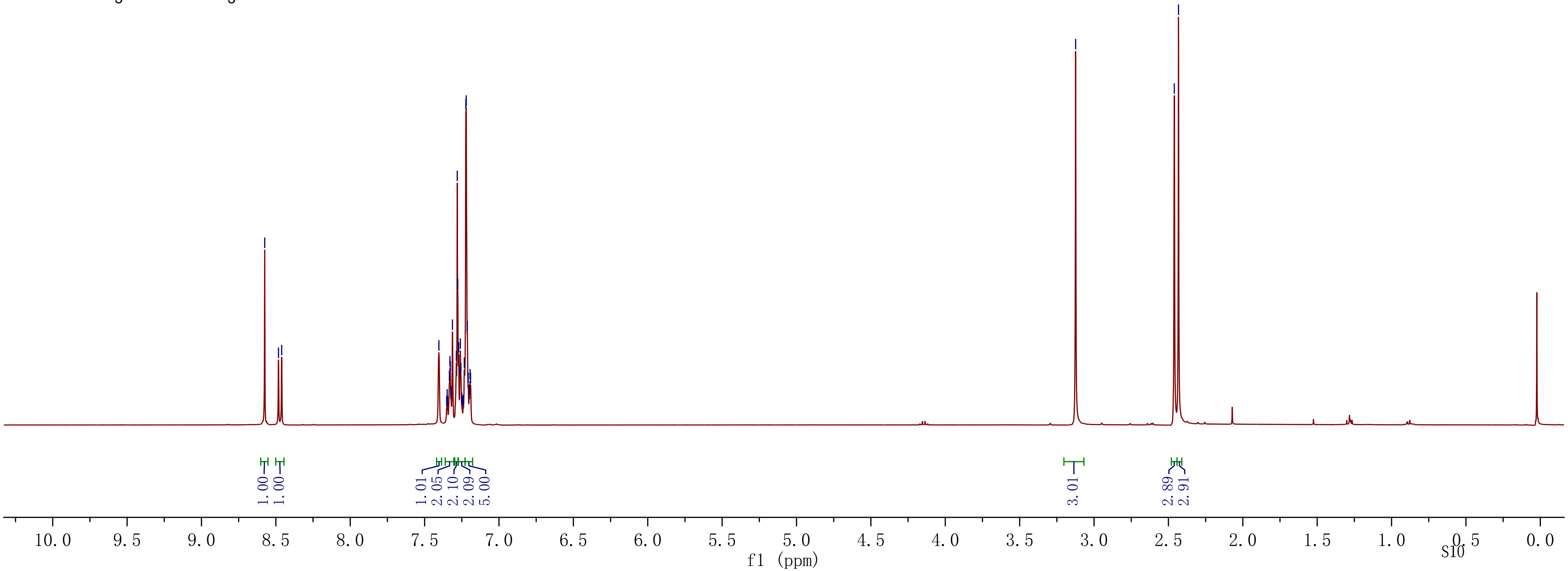

20220117zm
F210607

Figure S8, ^{13}C NMR spectrum of *N,N-dimethyl-4-(5-methyl-2,3-diphenyl-1H-indol-1-yl)-1,3,5-triazin-2-amine* **(3d)** in CDCl3

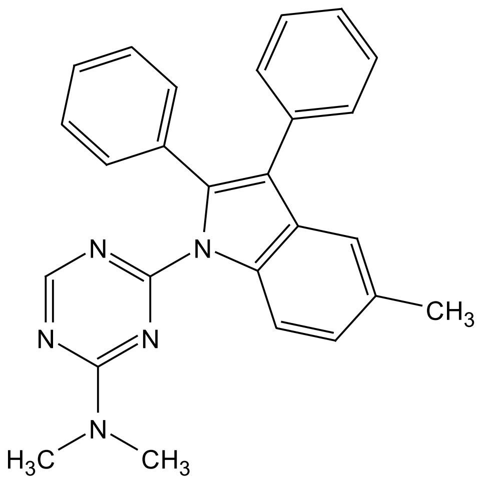

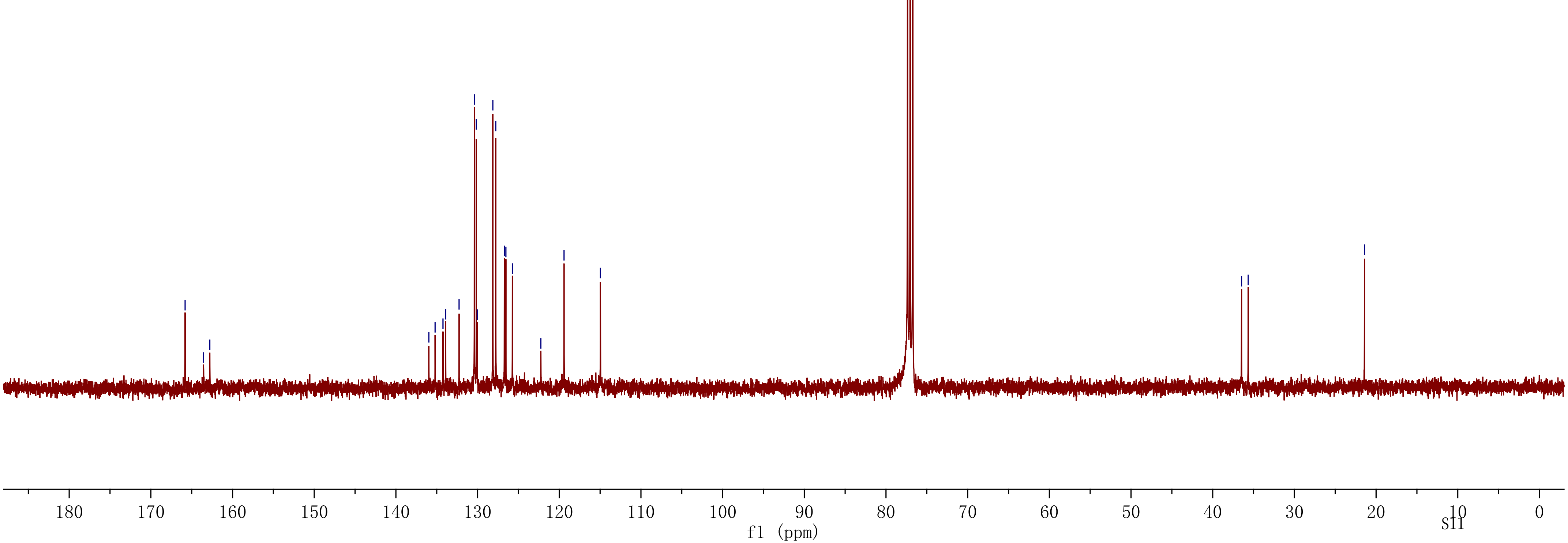

F-20220222
F20220222

Figure S9, ^{1}H NMR spectrum of *4-(7-methoxy-2,3-diphenyl-1H-indol-1-yl)-N,N-dimethyl-1,3,5-triazin-2-amine* **(3e) in CDCl3**

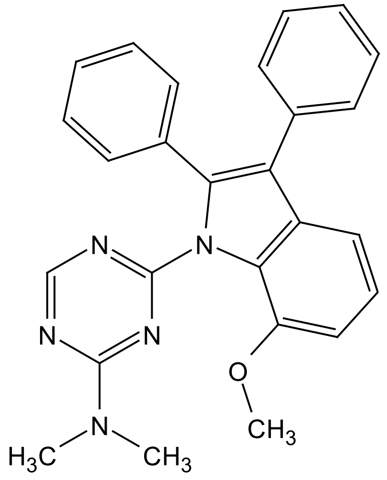

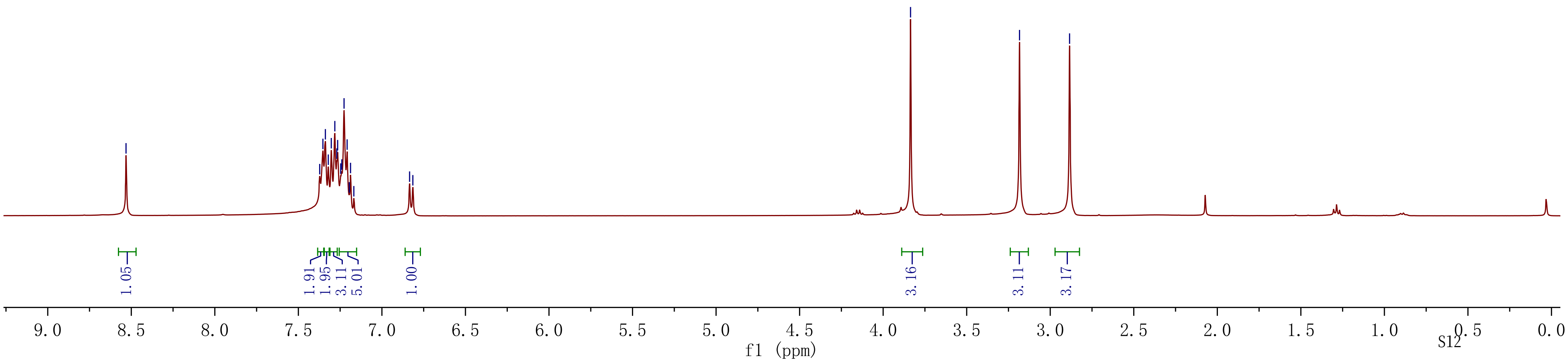

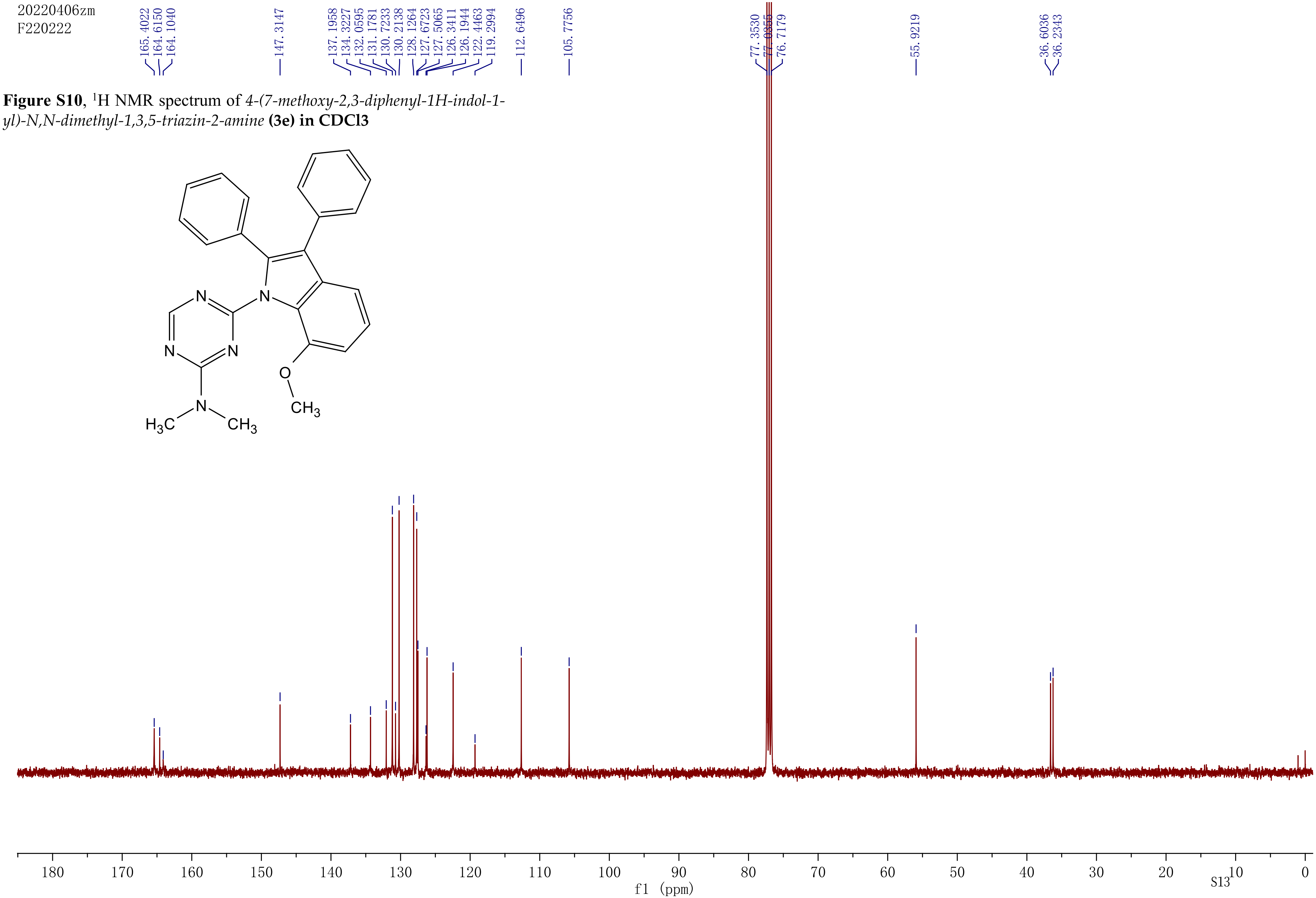

Figure S10, ^{1}H NMR spectrum of *4-(7-methoxy-2,3-diphenyl-1H-indol-1-yl)-N,N-dimethyl-1,3,5-triazin-2-amine* **(3e) in CDCl3**

20190419-H1-WHF-327
20190419-H1-WHF-327
CDCl3

8.5291, 8.4955, 8.4804, 7.3178, 7.3153, 7.3046, 7.2978, 7.2927, 7.2595, 7.2512, 7.2482, 7.2388, 7.2152, 7.2129, 7.2079, 7.2002, 7.1972, 7.1930, 7.1851, 7.1830, 7.1789, 7.0574, 7.0531, 6.9911, 6.9868, 6.9760, 6.9717, 3.8273, 3.0802, 2.3973, 1.6003

Figure S11, ^{1}H NMR spectrum of *4-(5-methoxy-2,3-diphenyl-1H-indol-1-yl)-N,N-dimethyl-1,3,5-triazin-2-amine* **(3f) in CDCl3**

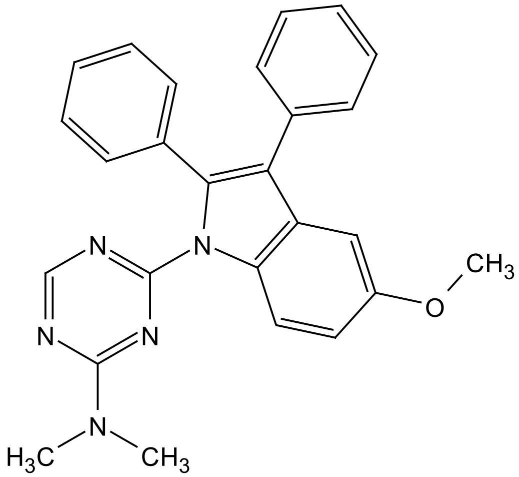

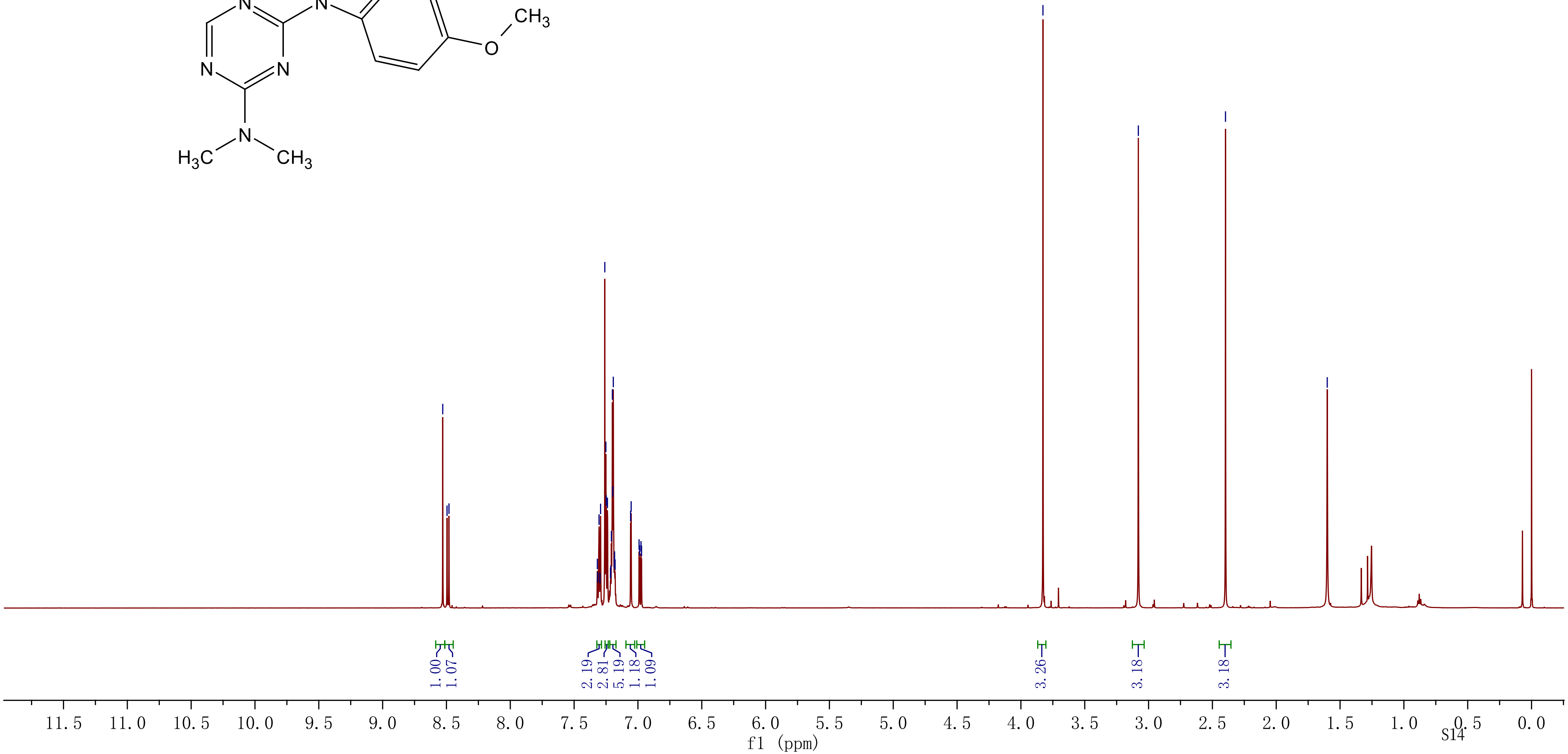

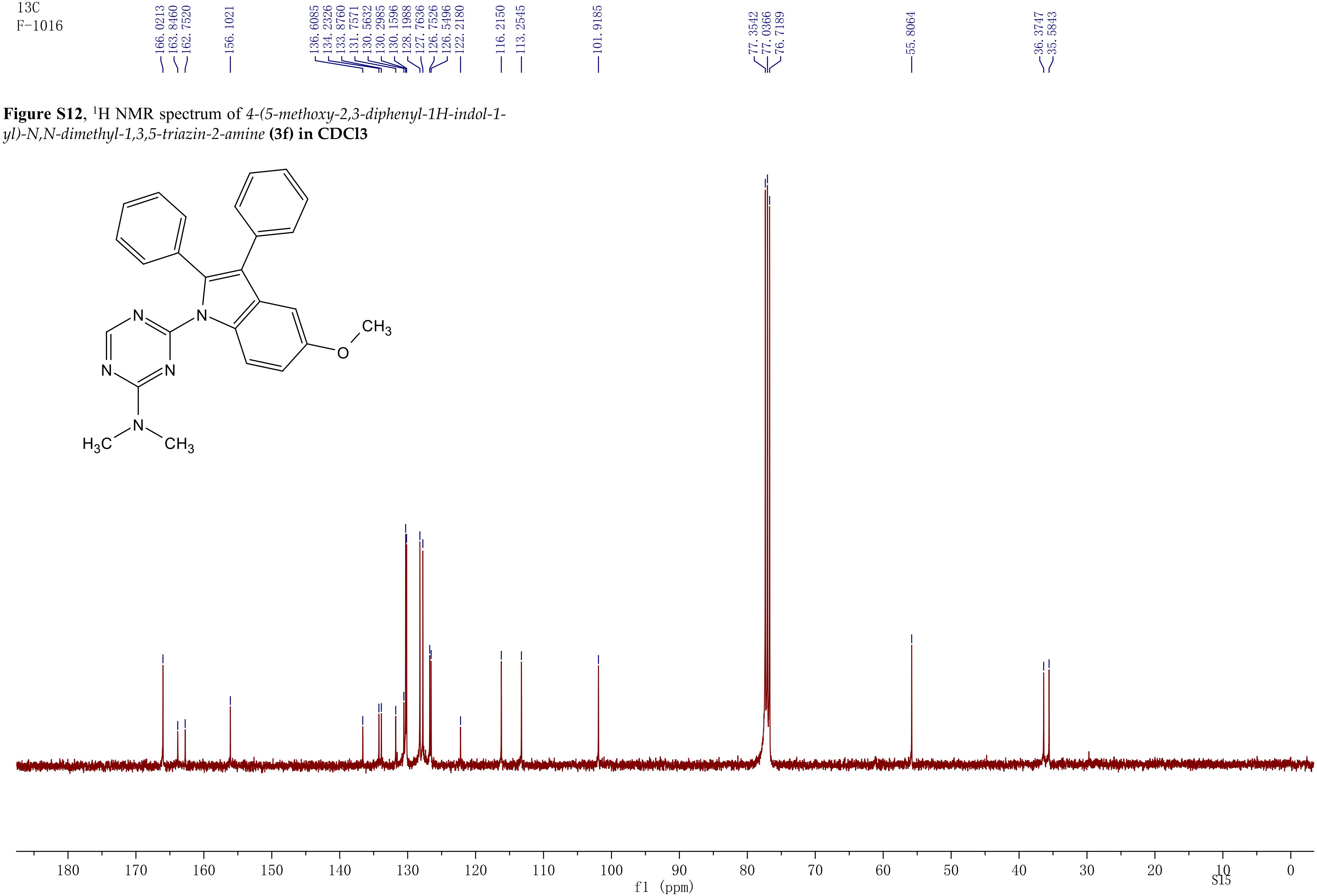

Figure S12, ^{1}H NMR spectrum of *4-(5-methoxy-2,3-diphenyl-1H-indol-1-yl)-N,N-dimethyl-1,3,5-triazin-2-amine* **(3f) in CDCl3**

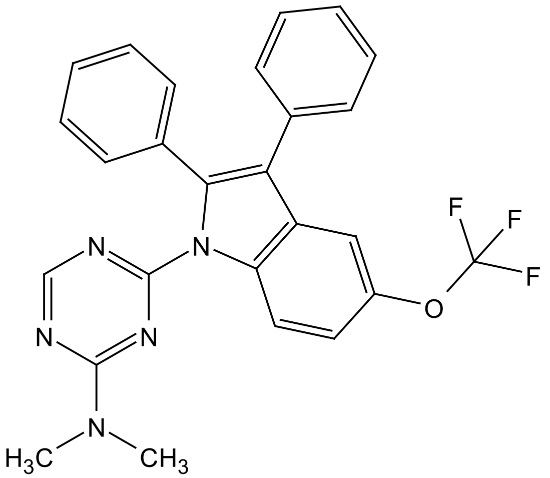

Figure S13, ^{1}H NMR spectrum of *4-(2,3-diphenyl-5-(trifluoromethoxy)-1H-indol-1-yl)-N,N-dimethyl-1,3,5-triazin-2-amine* **(3g) in CDCl3**

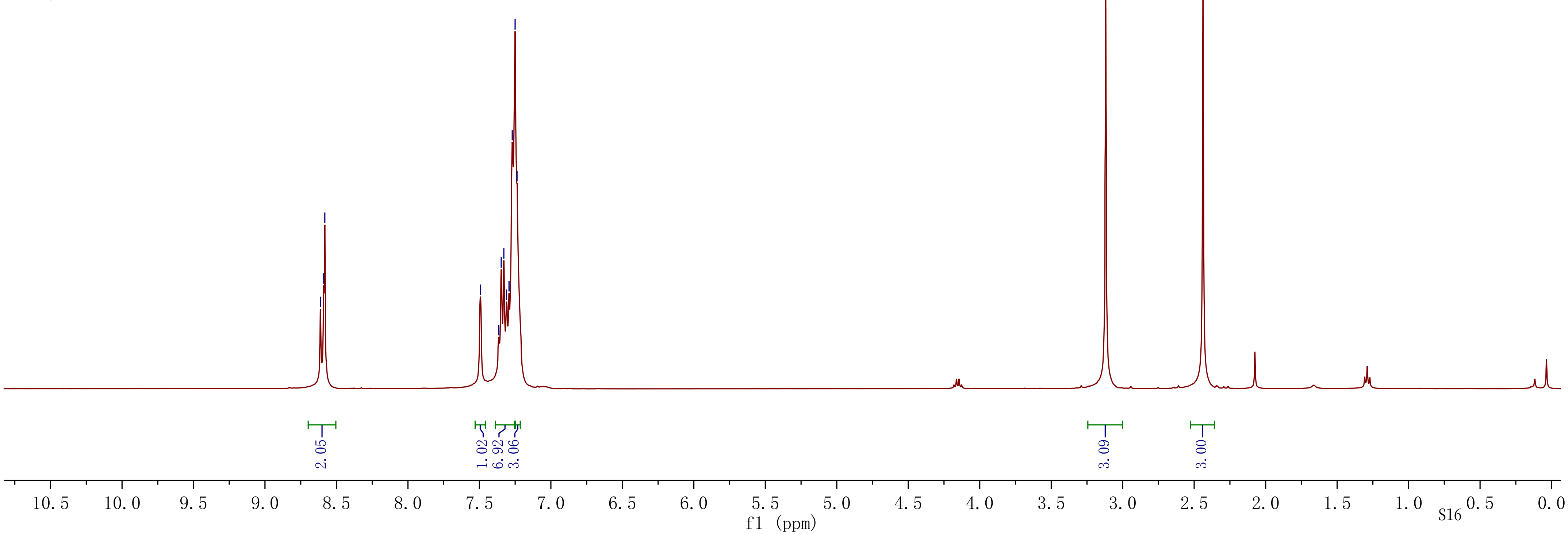

20220526zm
YZX1

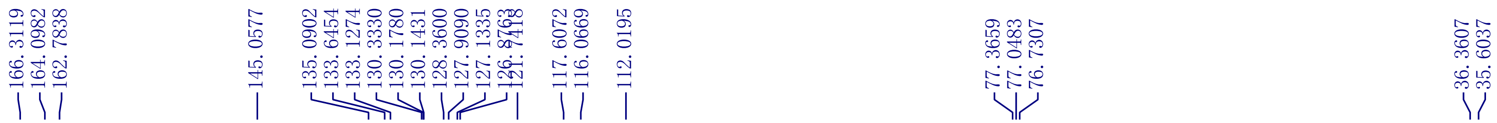

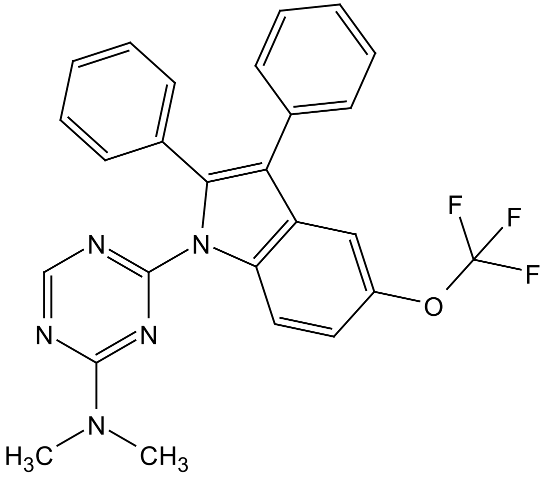

Figure S14, ^{13}C NMR spectrum of *4-(2,3-diphenyl-5-(trifluoromethoxy)-1H-indol-1-yl)-N,N-dimethyl-1,3,5-triazin-2-amine* **(3g) in CDCl3**

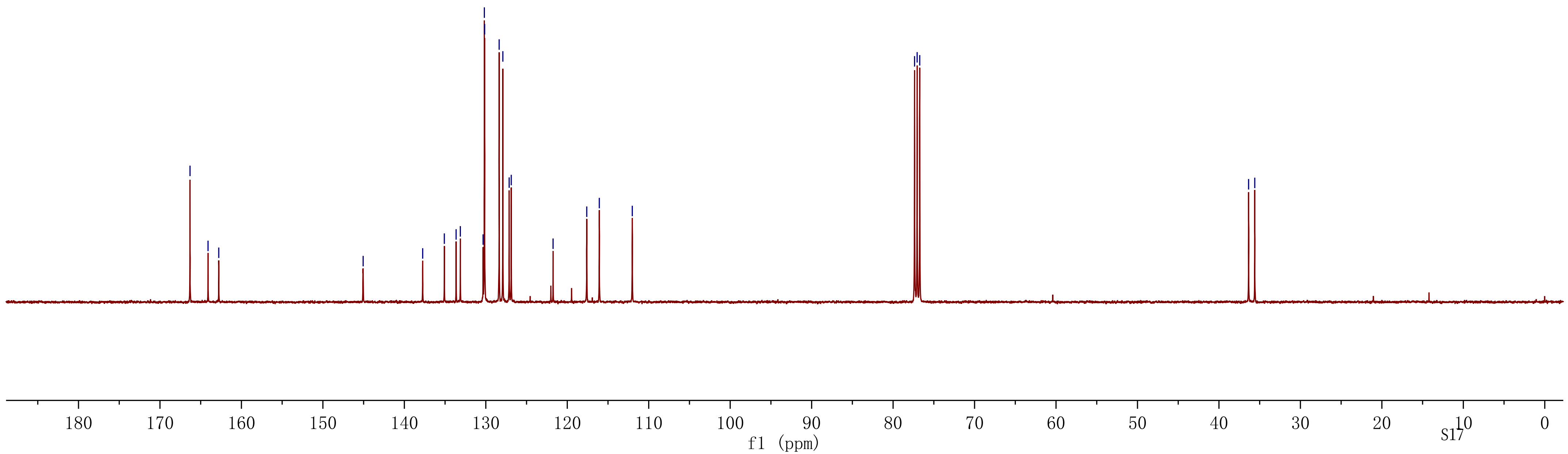

LFY211015
F-1015

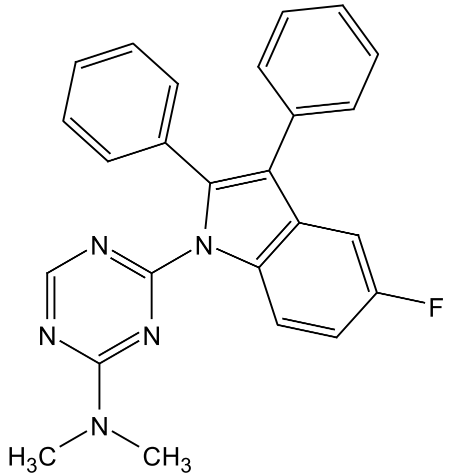

Figure S15, ^{1}H NMR spectrum of *4-(5-fluoro-2,3-diphenyl-1H-indol-1-yl)-N,N-dimethyl-1,3,5-triazin-2-amine* **(3h) in CDCl3**

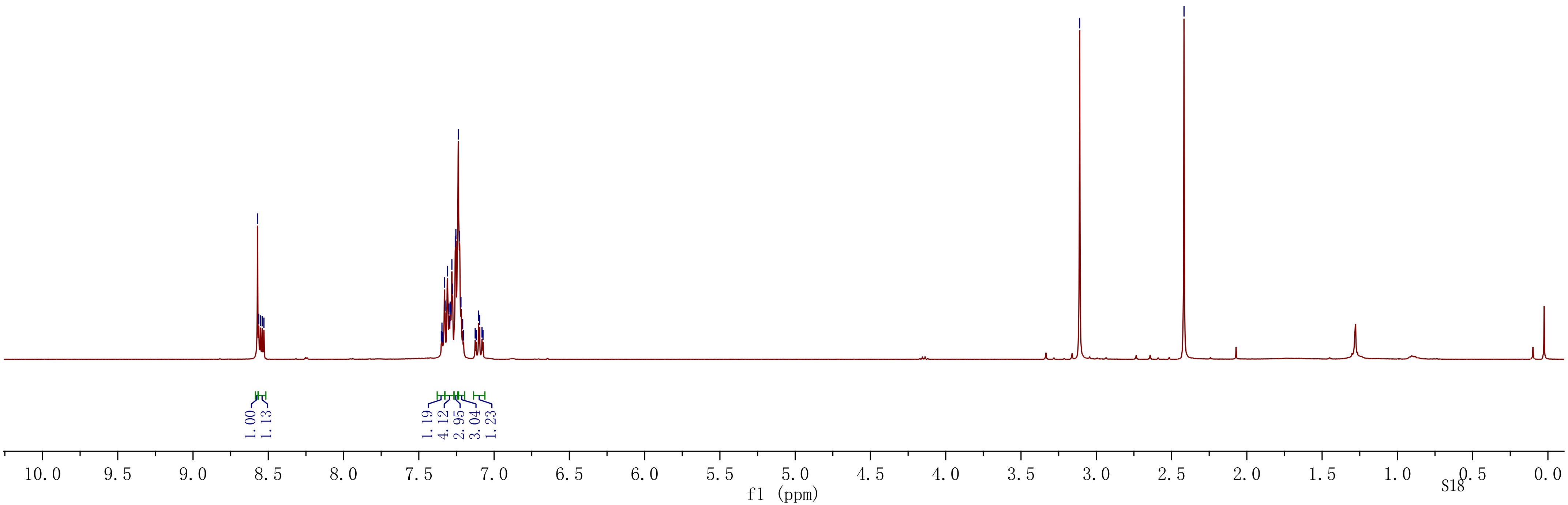

13C
F-1015

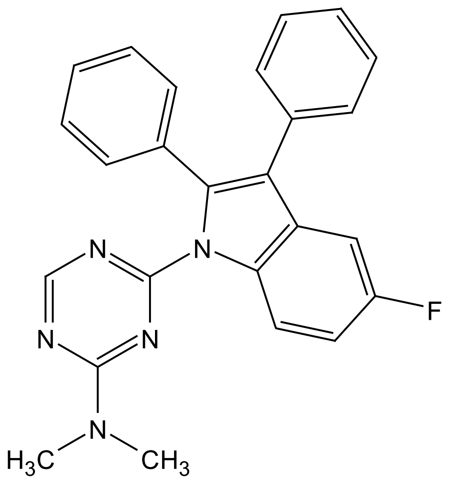

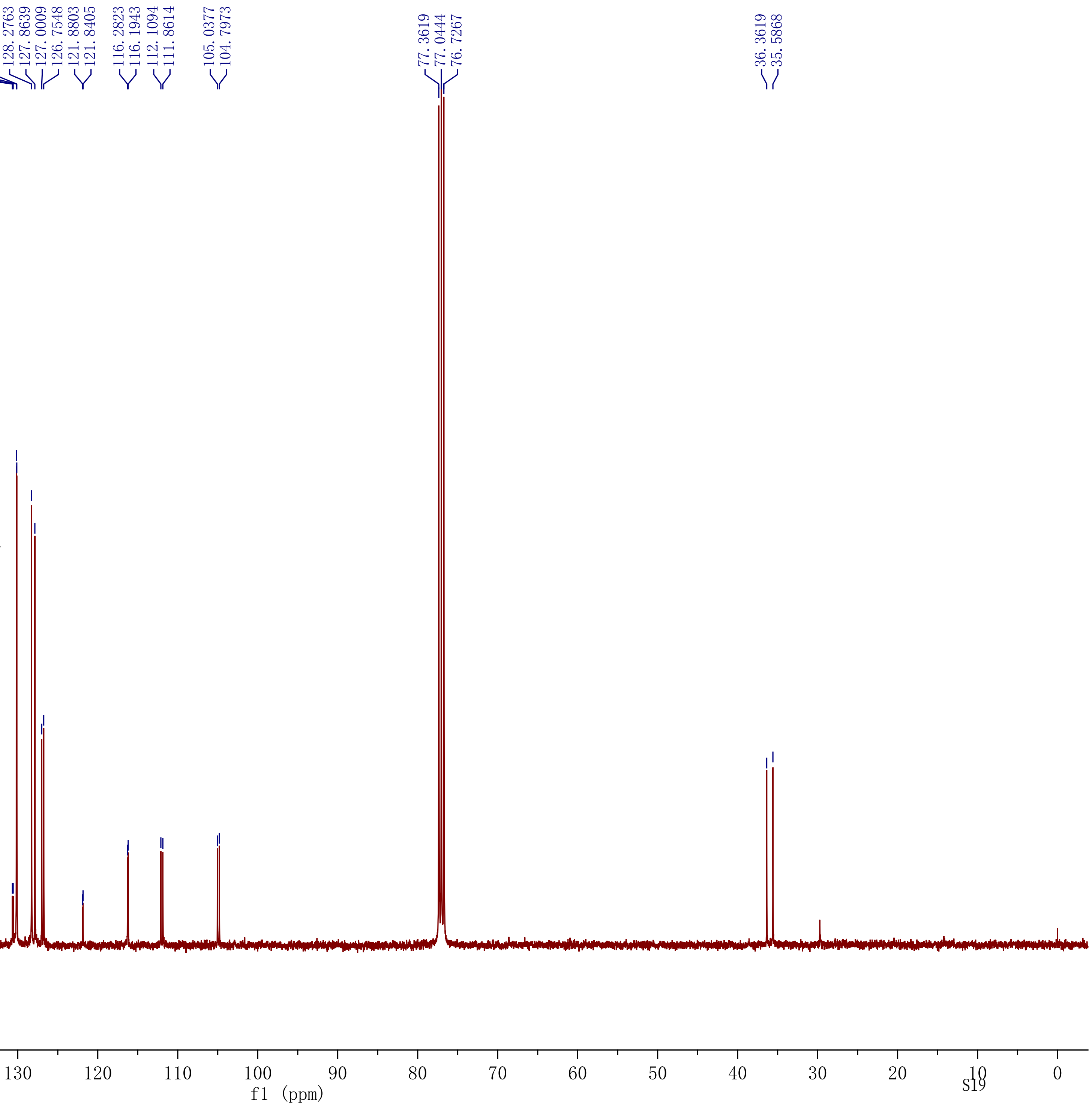

Figure S16, ^{13}C NMR spectrum of *4-(5-fluoro-2,3-diphenyl-1H-indol-1-yl)-N,N-dimethyl-1,3,5-triazin-2-amine* **(3h) in CDCl3**

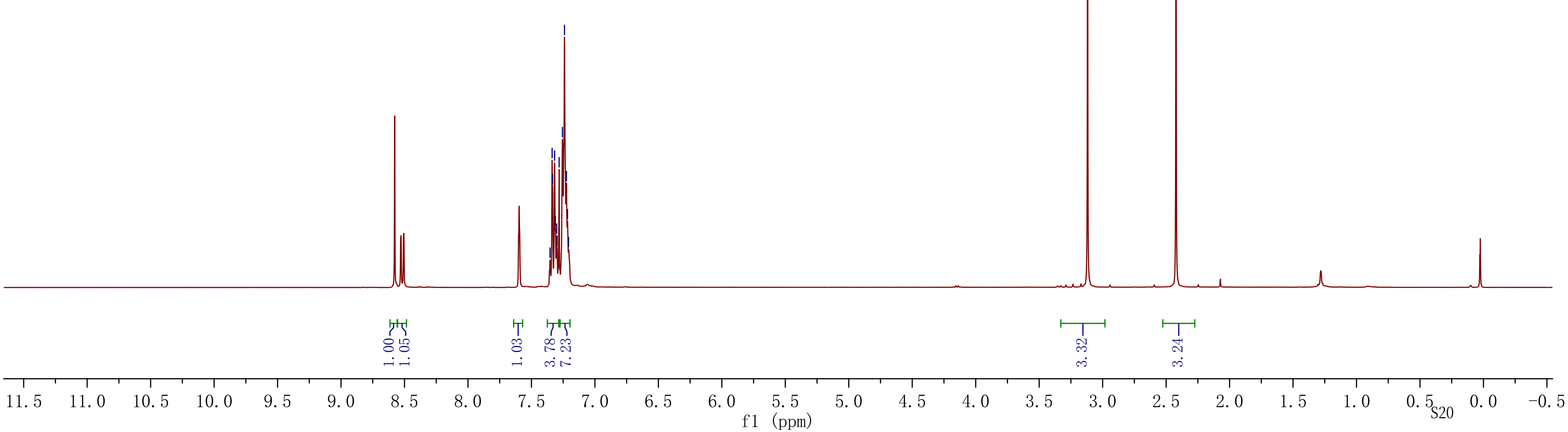

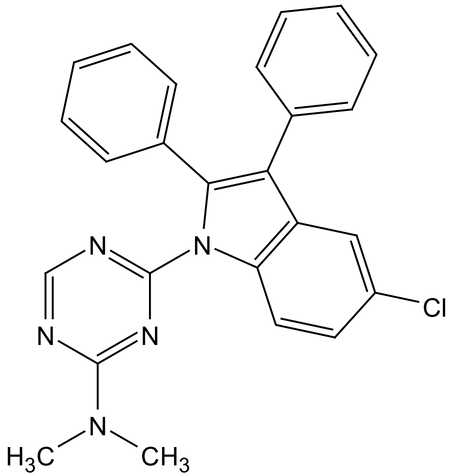

Figure S17, ^{1}H NMR spectrum of *4-(5-chloro-2,3-diphenyl-1H-indol-1-yl)-N,N-dimethyl-1,3,5-triazin-2-amine* **(3i) in CDCl3**

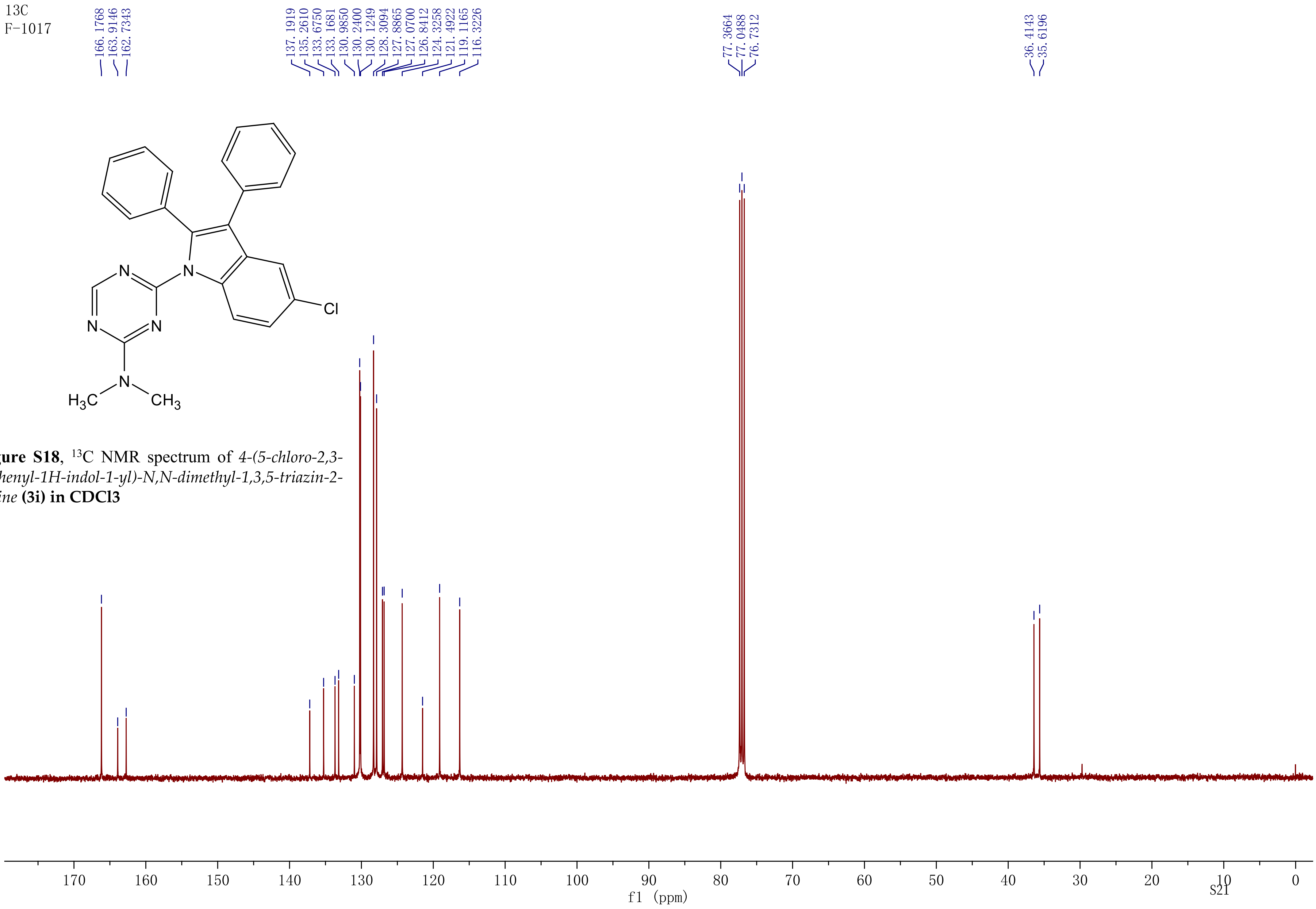

Figure S18, ^{13}C NMR spectrum of *4-(5-chloro-2,3-diphenyl-1H-indol-1-yl)-N,N-dimethyl-1,3,5-triazin-2-amine* **(3i) in CDCl3**

20211203zm
F-211116

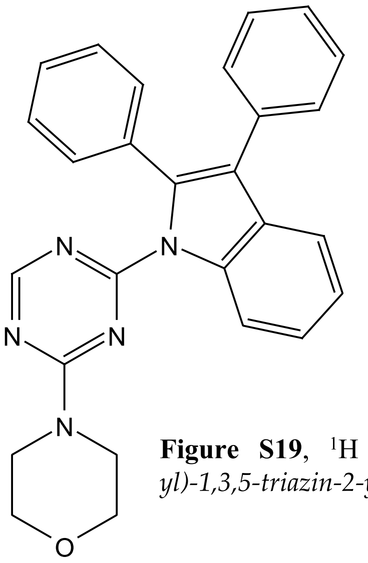

Figure S19, ^{1}H NMR spectrum of *4-(4-(2,3-diphenyl-1H-indol-1-yl)-1,3,5-triazin-2-yl)morpholine* **(3j) in CDCl3**

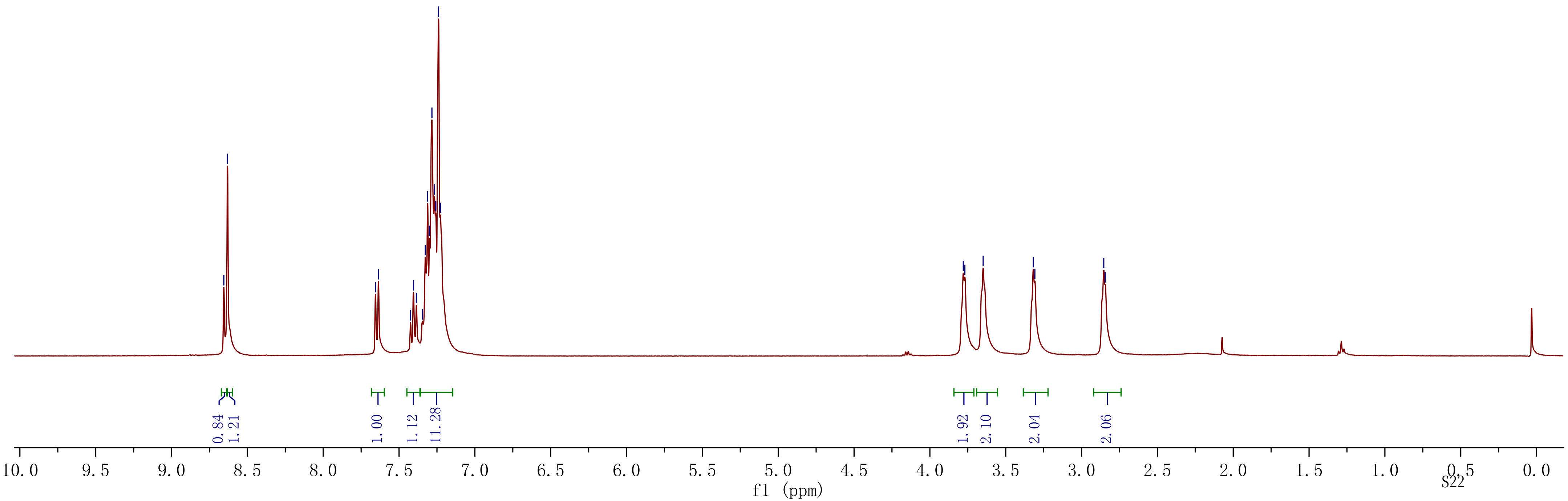

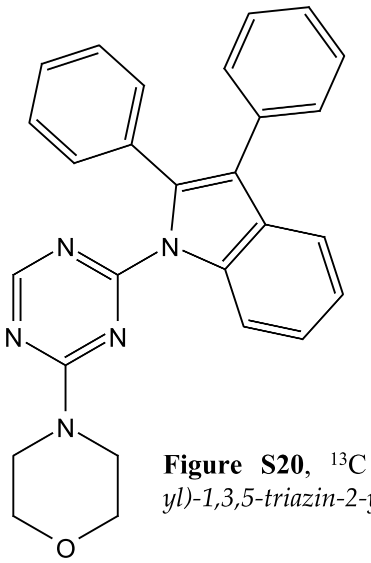

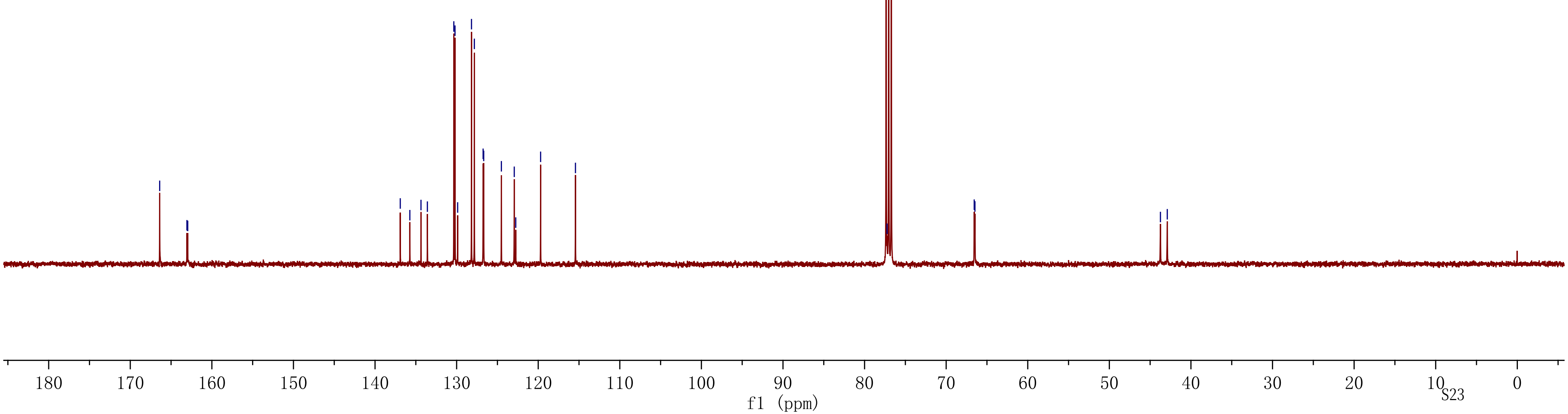

Figure S20, ^{13}C NMR spectrum of *4-(4-(2,3-diphenyl-1H-indol-1-yl)-1,3,5-triazin-2-yl)morpholine* **(3j) in CDCl3**

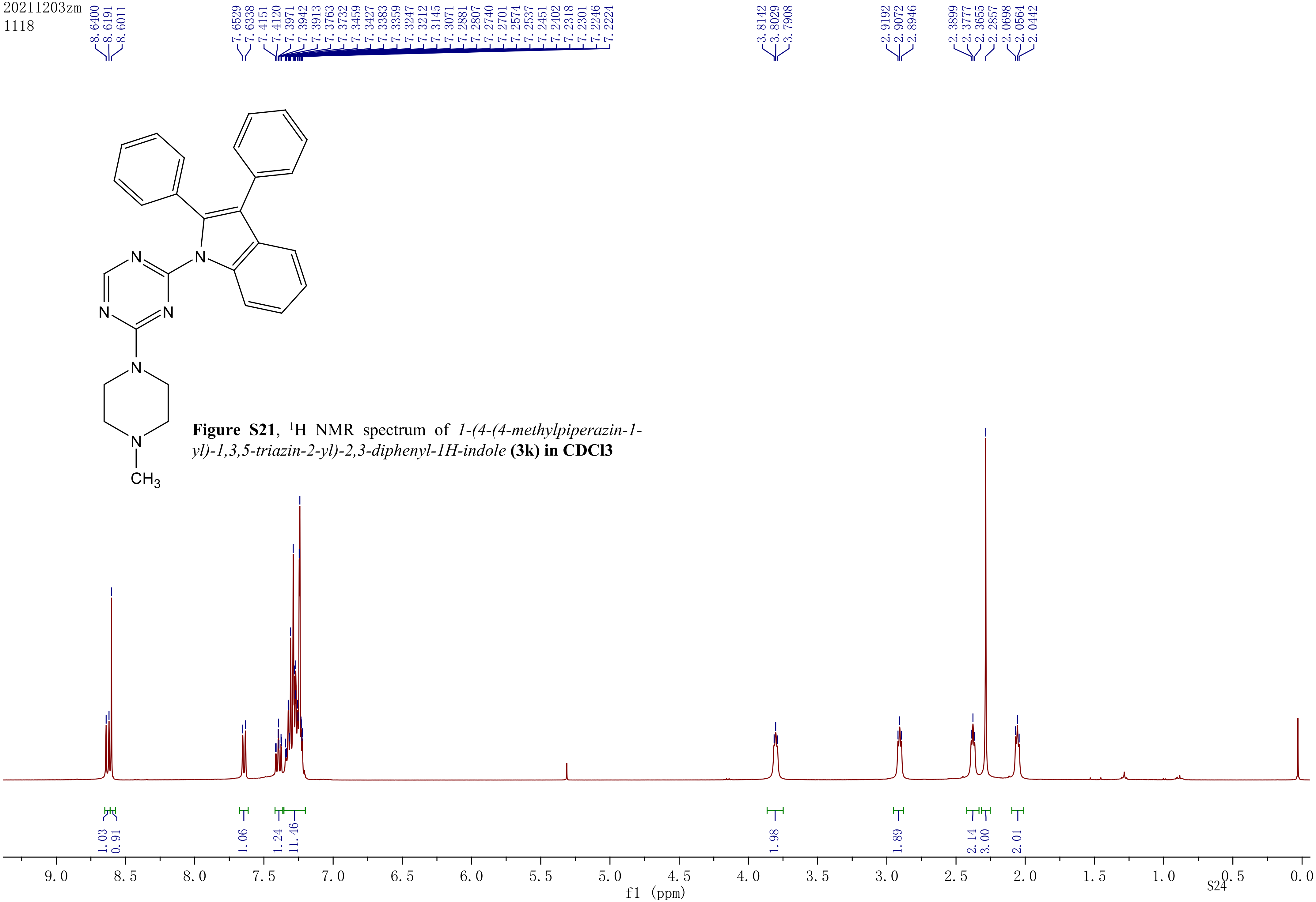

Figure S21, ^{1}H NMR spectrum of *1-(4-(4-methylpiperazin-1-yl)-1,3,5-triazin-2-yl)-2,3-diphenyl-1H-indole* **(3k) in CDCl3**

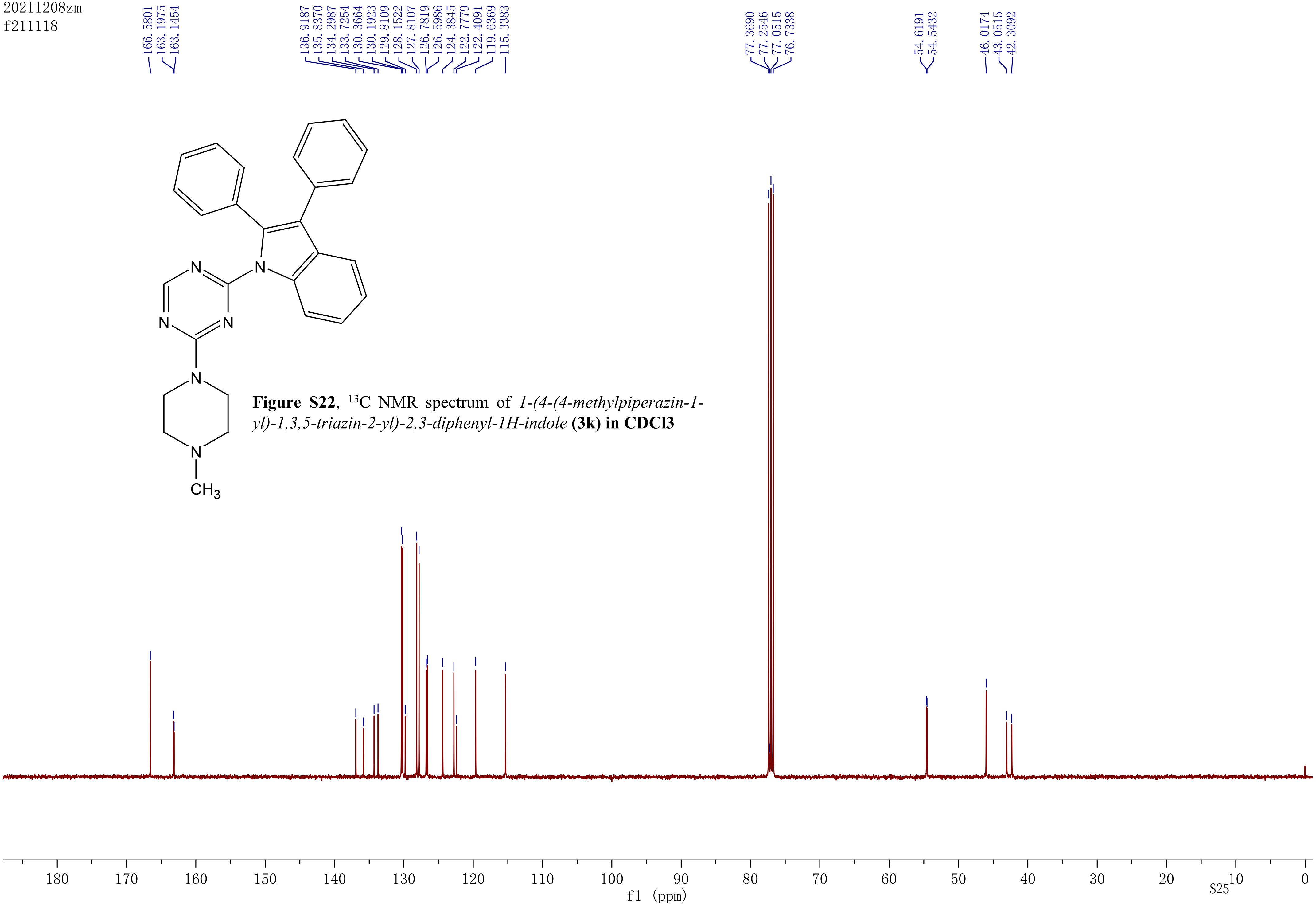

Figure S22, ^{13}C NMR spectrum of *1-(4-(4-methylpiperazin-1-yl)-1,3,5-triazin-2-yl)-2,3-diphenyl-1H-indole* **(3k) in CDCl3**

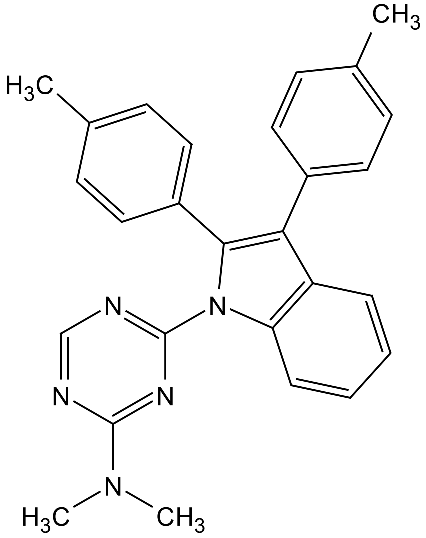

Figure S23, ^{1}H NMR spectrum of *4-(2,3-di-p-tolyl-1H-indol-1-yl)-N,N-dimethyl-1,3,5-triazin-2-amine* **(3m) in CDCl3**

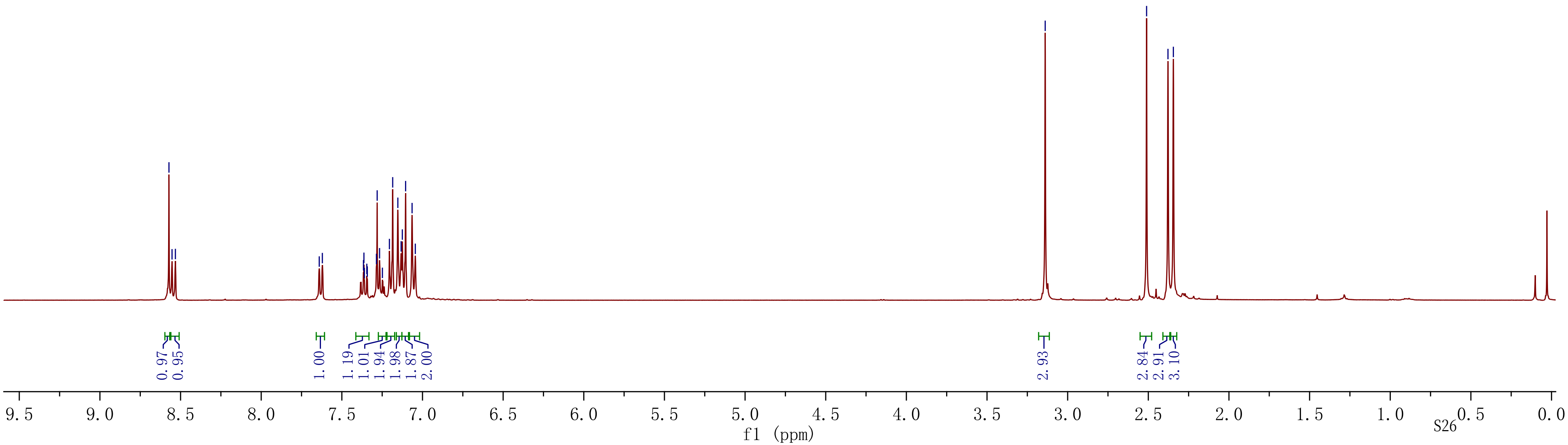

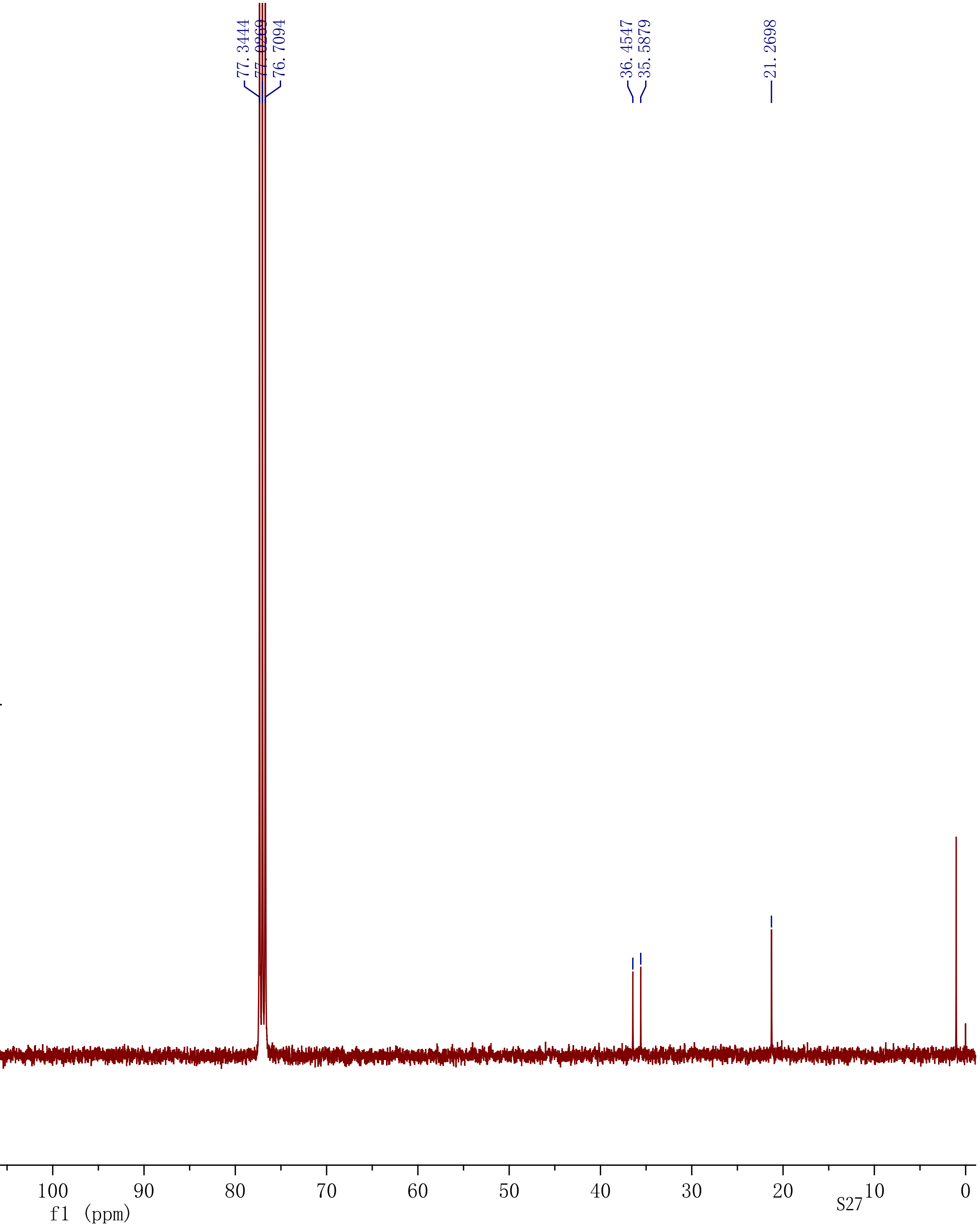

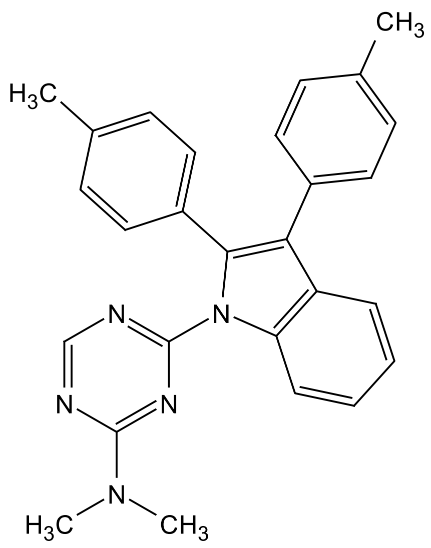

Figure S24, ^{13}C NMR spectrum of *4-(2,3-di-p-tolyl-1H-indol-1-yl)-N,N-dimethyl-1,3,5-triazin-2-amine* **(3m) in CDCl3**

20220112zm
F211209

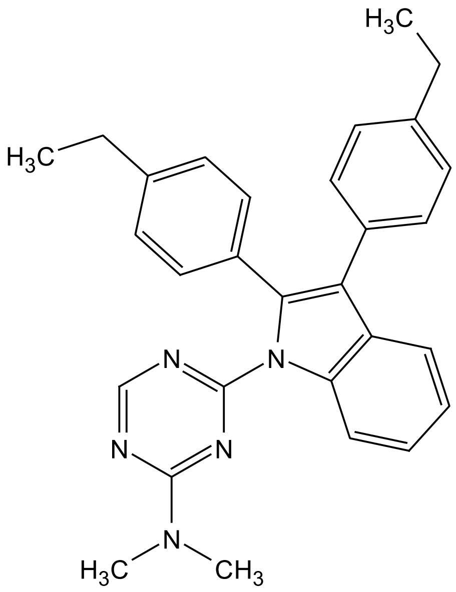

Figure S25, ^{1}H NMR spectrum of *4-(2,3-bis(4-ethylphenyl)-1H-indol-1-yl)-N,N-dimethyl-1,3,5-triazin-2-amine* **(3n) in CDCl3**

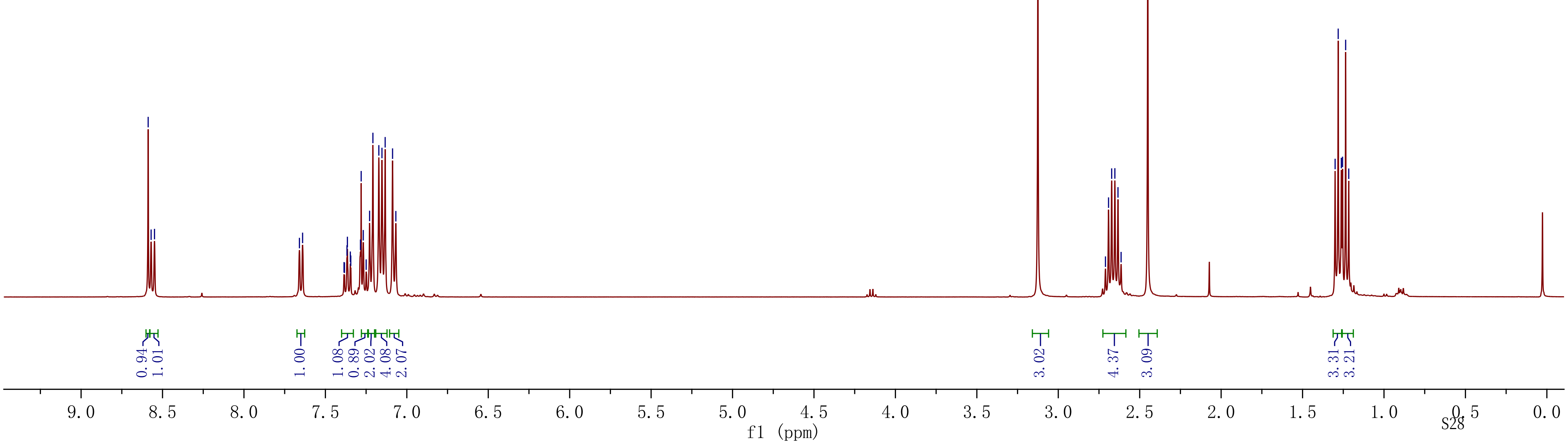

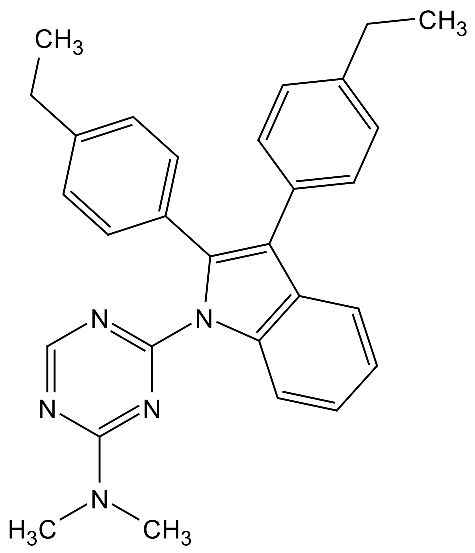

Figure S26, ^{13}C NMR spectrum of *4-(2,3-bis(4-ethylphenyl)-1H-indol-1-yl)-N,N-dimethyl-1,3,5-triazin-2-amine* **(3n) in CDCl3**

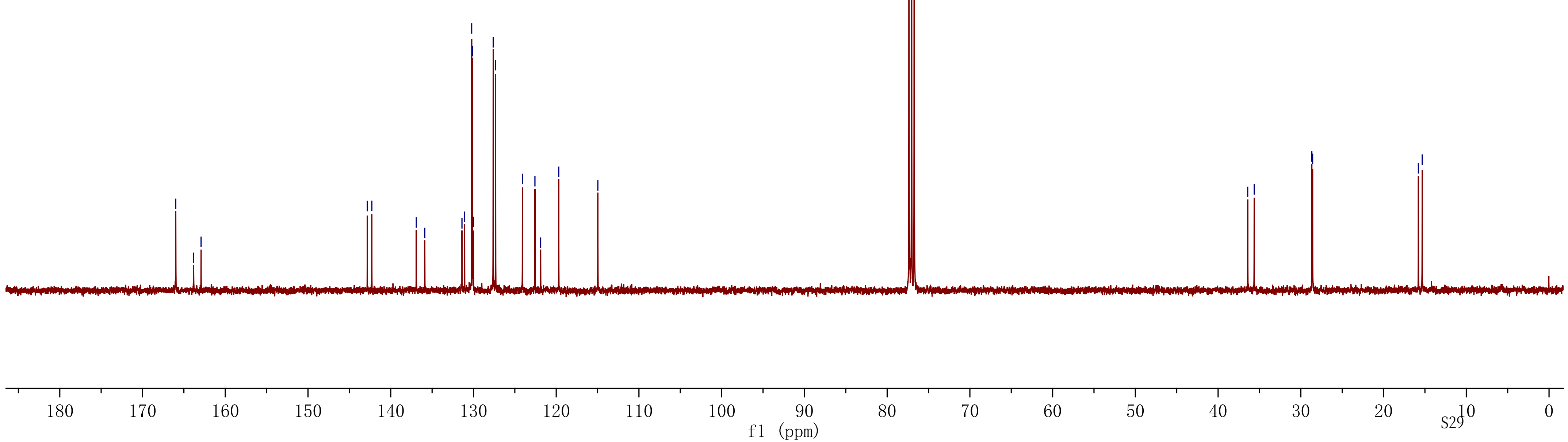

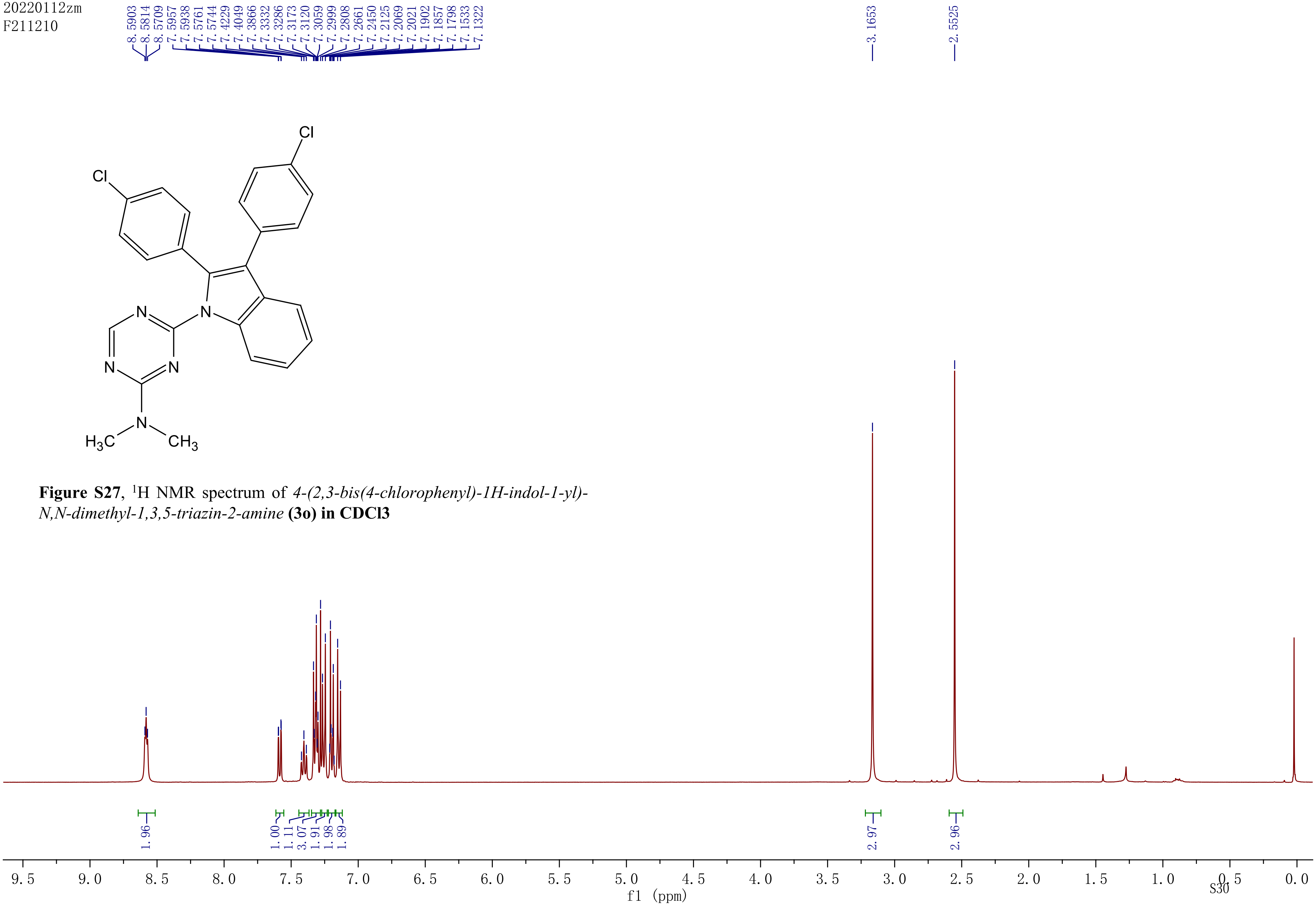

Figure S27, ^{1}H NMR spectrum of *4-(2,3-bis(4-chlorophenyl)-1H-indol-1-yl)-N,N-dimethyl-1,3,5-triazin-2-amine* **(3o) in CDCl3**

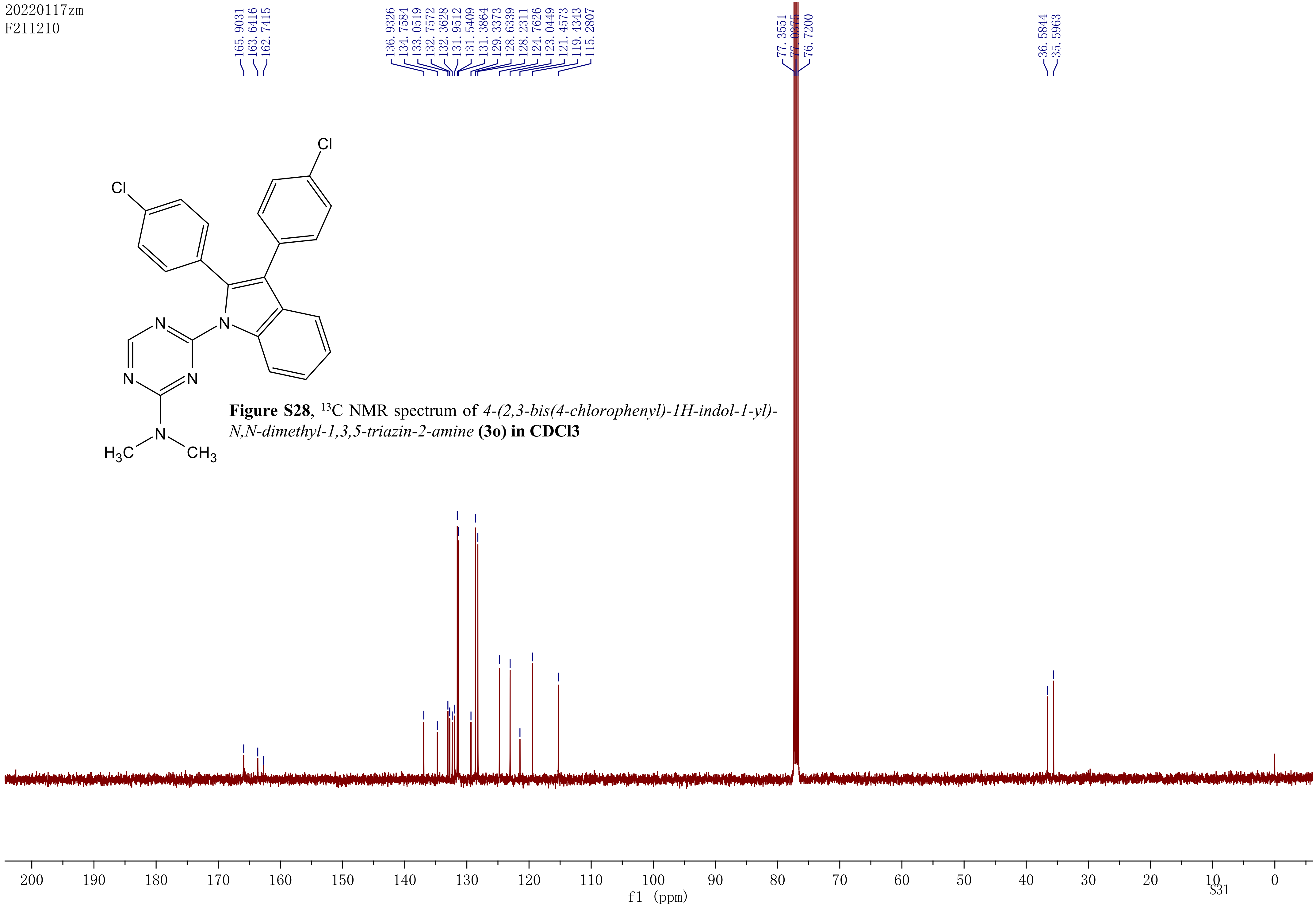

Figure S28, ^{13}C NMR spectrum of *4-(2,3-bis(4-chlorophenyl)-1H-indol-1-yl)-N,N-dimethyl-1,3,5-triazin-2-amine* **(3o) in CDCl3**

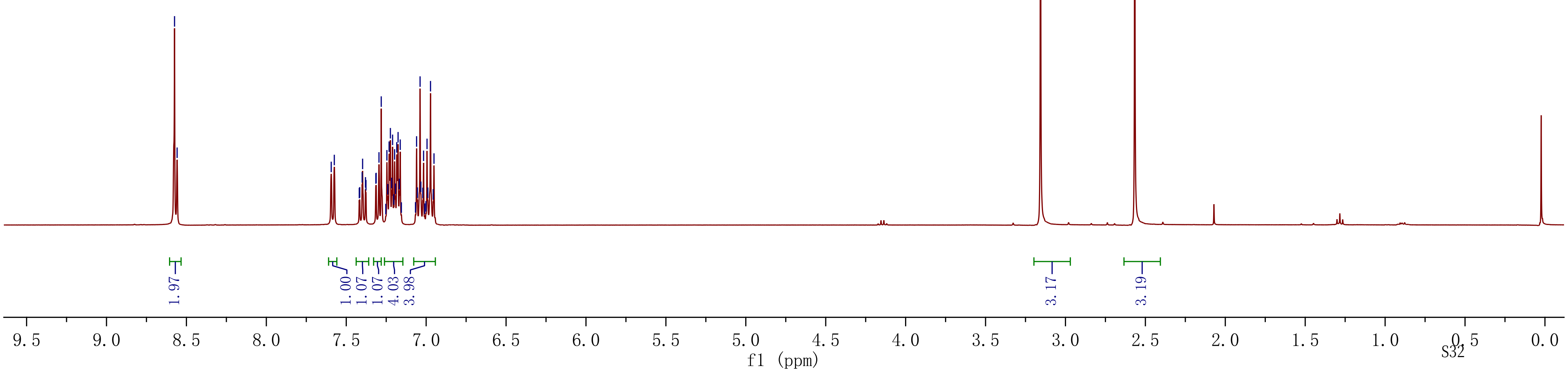

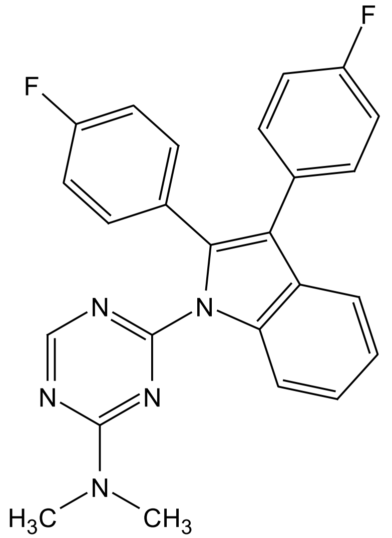

Figure S29, ^{1}H NMR spectrum of *4-(2,3-bis(4-fluorophenyl)-1H-indol-1-yl)-N,N-dimethyl-1,3,5-triazin-2-amine* **(3p) in CDCl3**

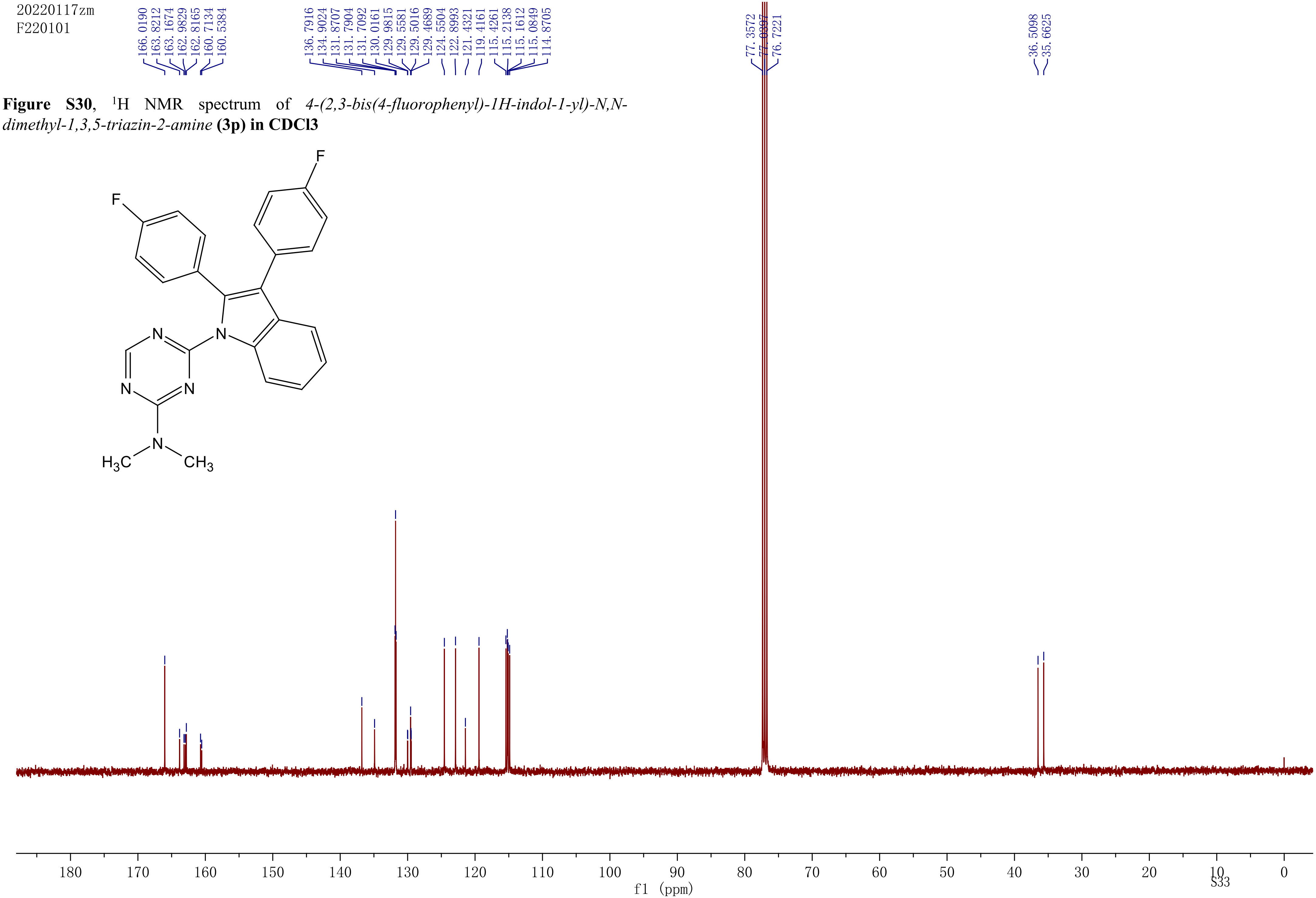

Figure S30, ^{1}H NMR spectrum of *4-(2,3-bis(4-fluorophenyl)-1H-indol-1-yl)-N,N-dimethyl-1,3,5-triazin-2-amine* **(3p) in CDCl3**

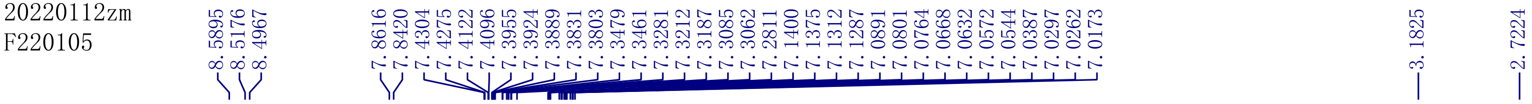

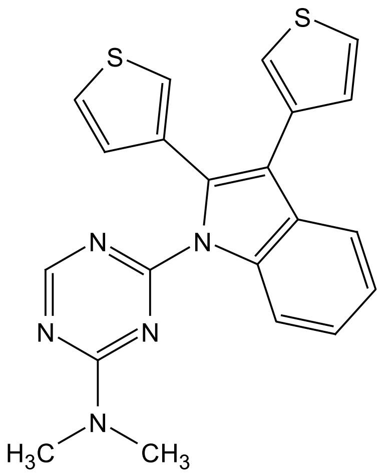

Figure S31, ^{1}H NMR spectrum of *4-(2,3-di(thiophen-2-yl)-1H-indol-1-yl)-N,N-dimethyl-1,3,5-triazin-2-amine* **(3q) in CDCl3**

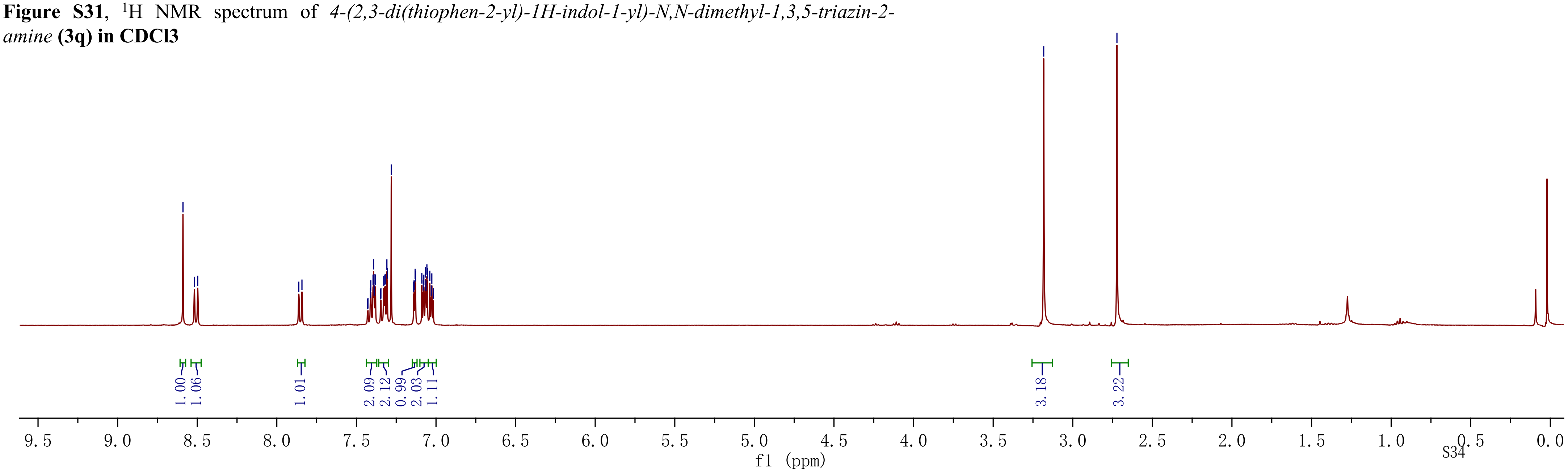

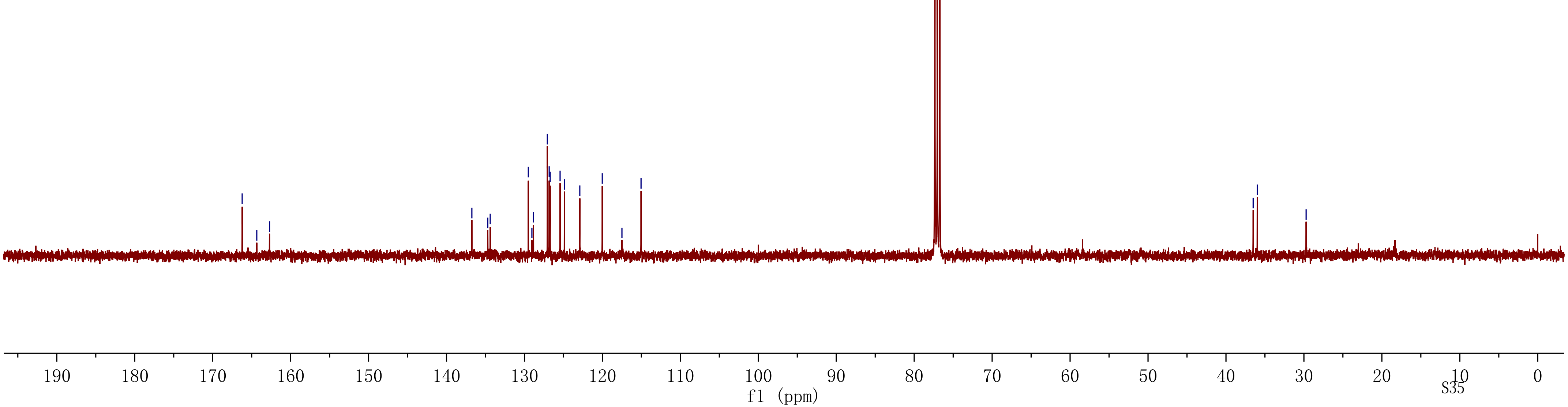

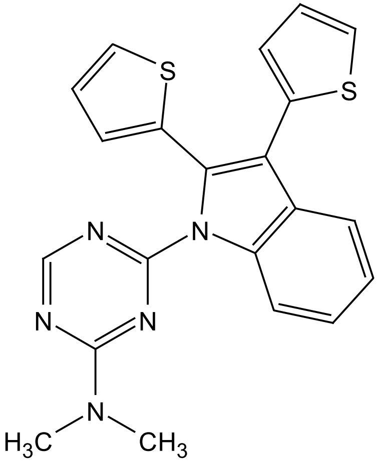

Figure S32, ^{13}C NMR spectrum of *4-(2,3-di(thiophen-2-yl)-1H-indol-1-yl)-N,N-dimethyl-1,3,5-triazin-2-amine* **(3q) in CDCl3**

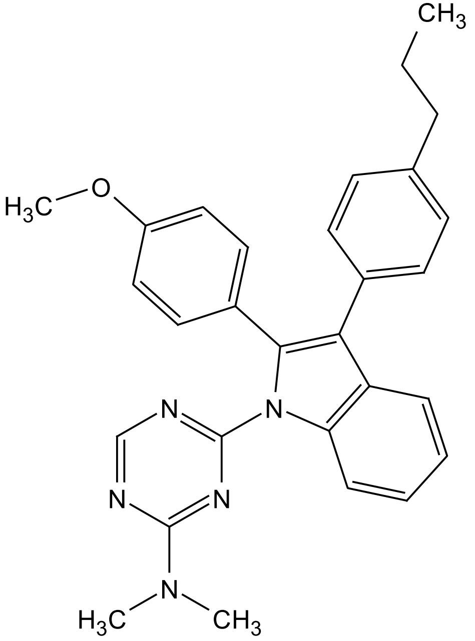

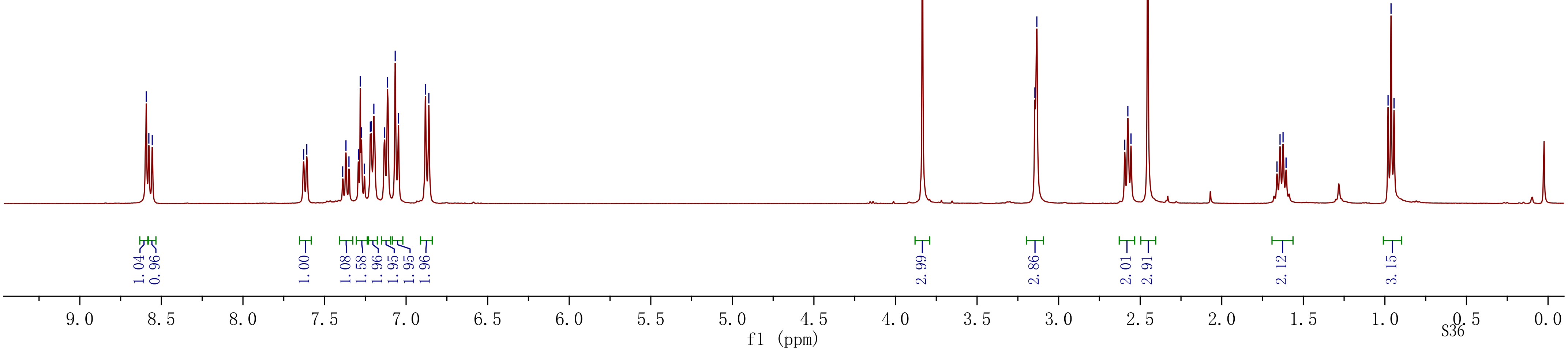

Figure S33, ^{1}H NMR spectrum of *4-(2-(4-methoxyphenyl)-3-(4-propylphenyl)-1H-indol-1-yl)-N,N-dimethyl-1,3,5-triazin-2-amine* **(3r** and **3r') in CDCl3**

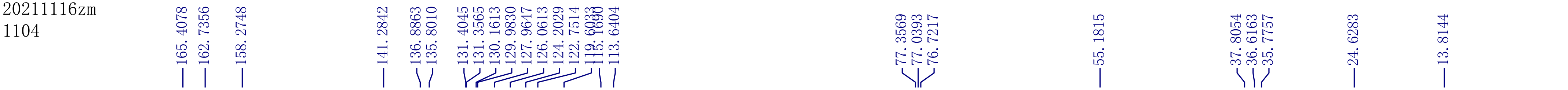

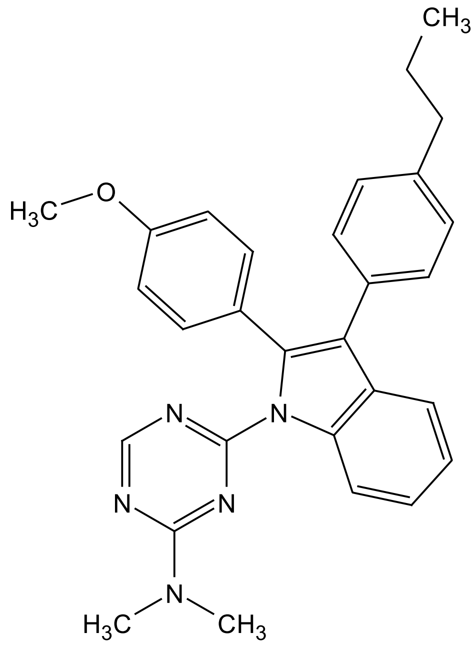

Figure S34, ^{13}C NMR spectrum of *4-(2-(4-methoxyphenyl)-3-(4-propylphenyl)-1H-indol-1-yl)-N,N-dimethyl-1,3,5-triazin-2-amine* **(3r** and **3r') in CDCl3**

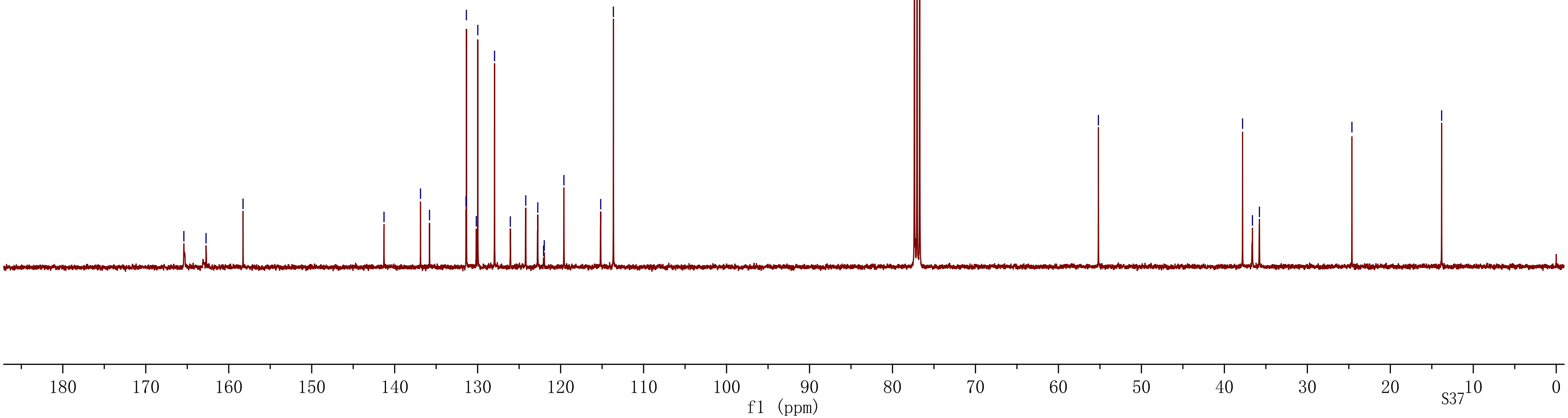

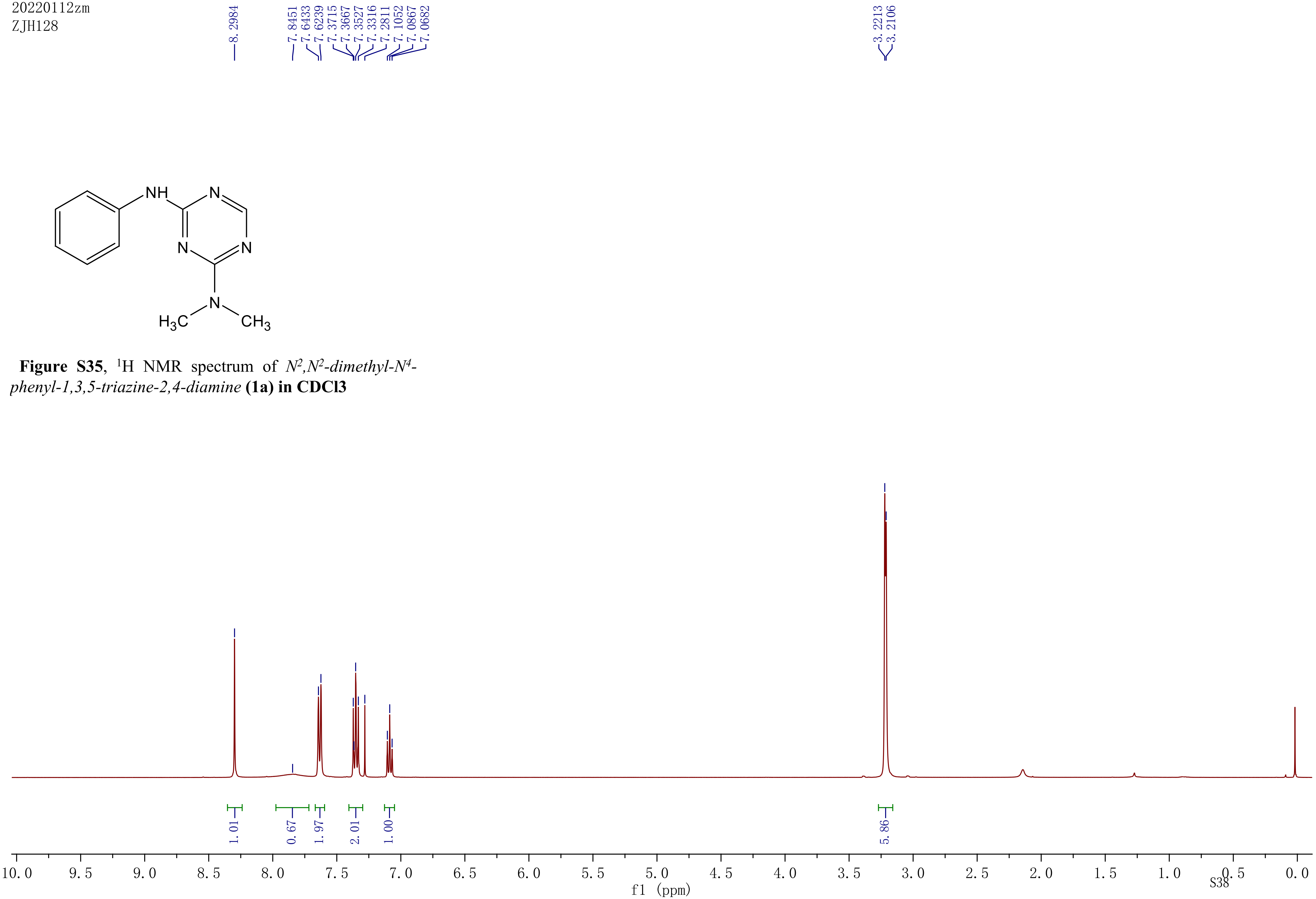

Figure S35, ^{1}H NMR spectrum of *N*[2]*,N*[2]*-dimethyl-N*[4]*-phenyl-1,3,5-triazine-2,4-diamine* **(1a) in CDCl3**

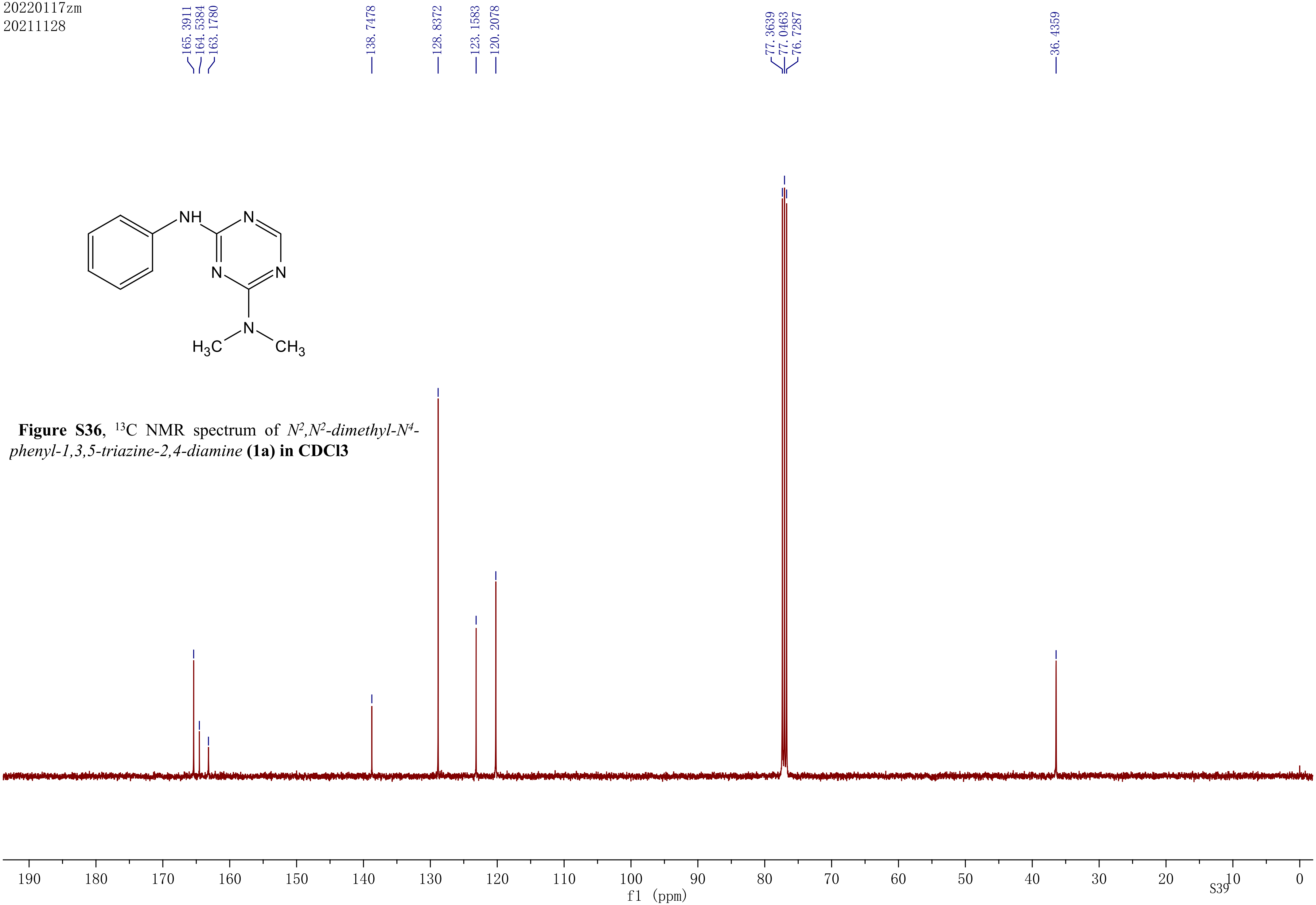

Figure S36, ^{13}C NMR spectrum of *N*[2],*N*[2]*-dimethyl-N*[4]*-phenyl-1,3,5-triazine-2,4-diamine* **(1a) in CDCl3**

20230412zm
1j CDCl3

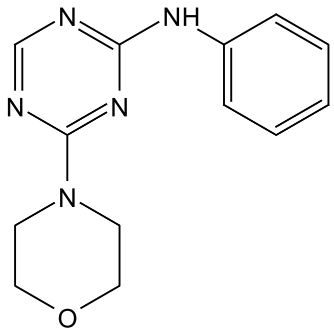

Figure S37, ^{1}H NMR spectrum of *4-morpholino-N-phenyl-1,3,5-triazin-2-amine* **(1j) in CDCl3**

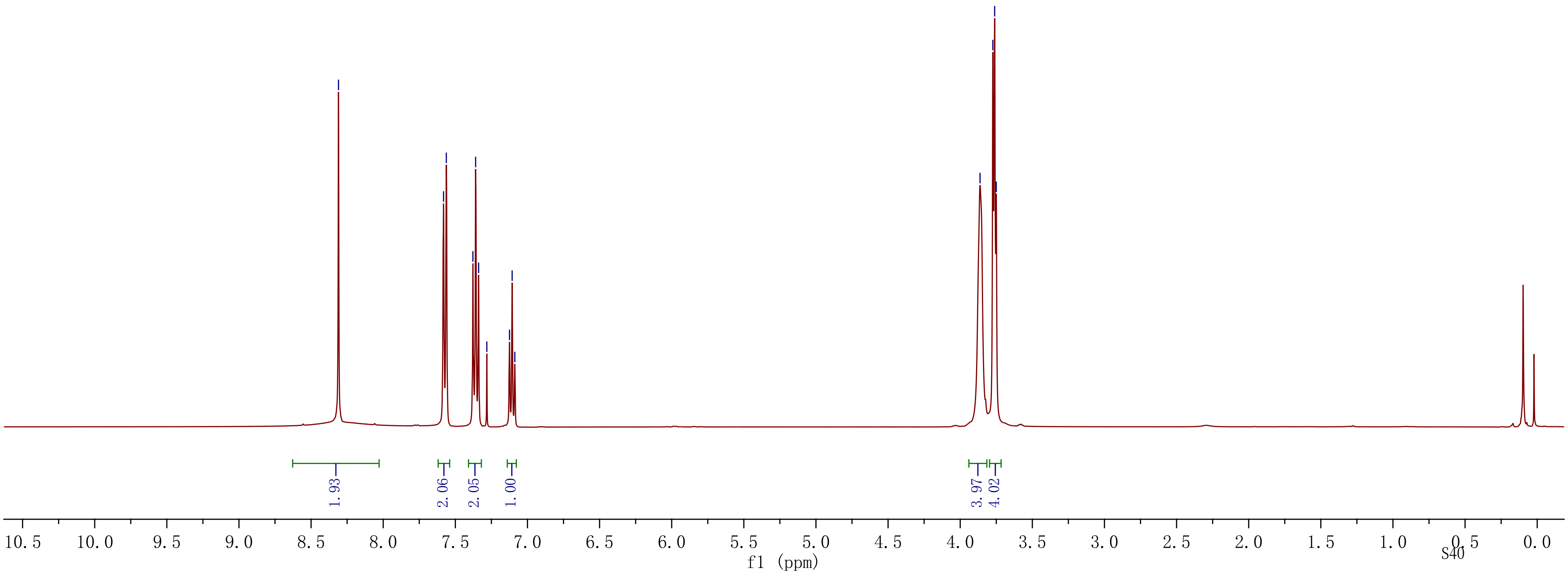

20230412zm
1j CDCl3

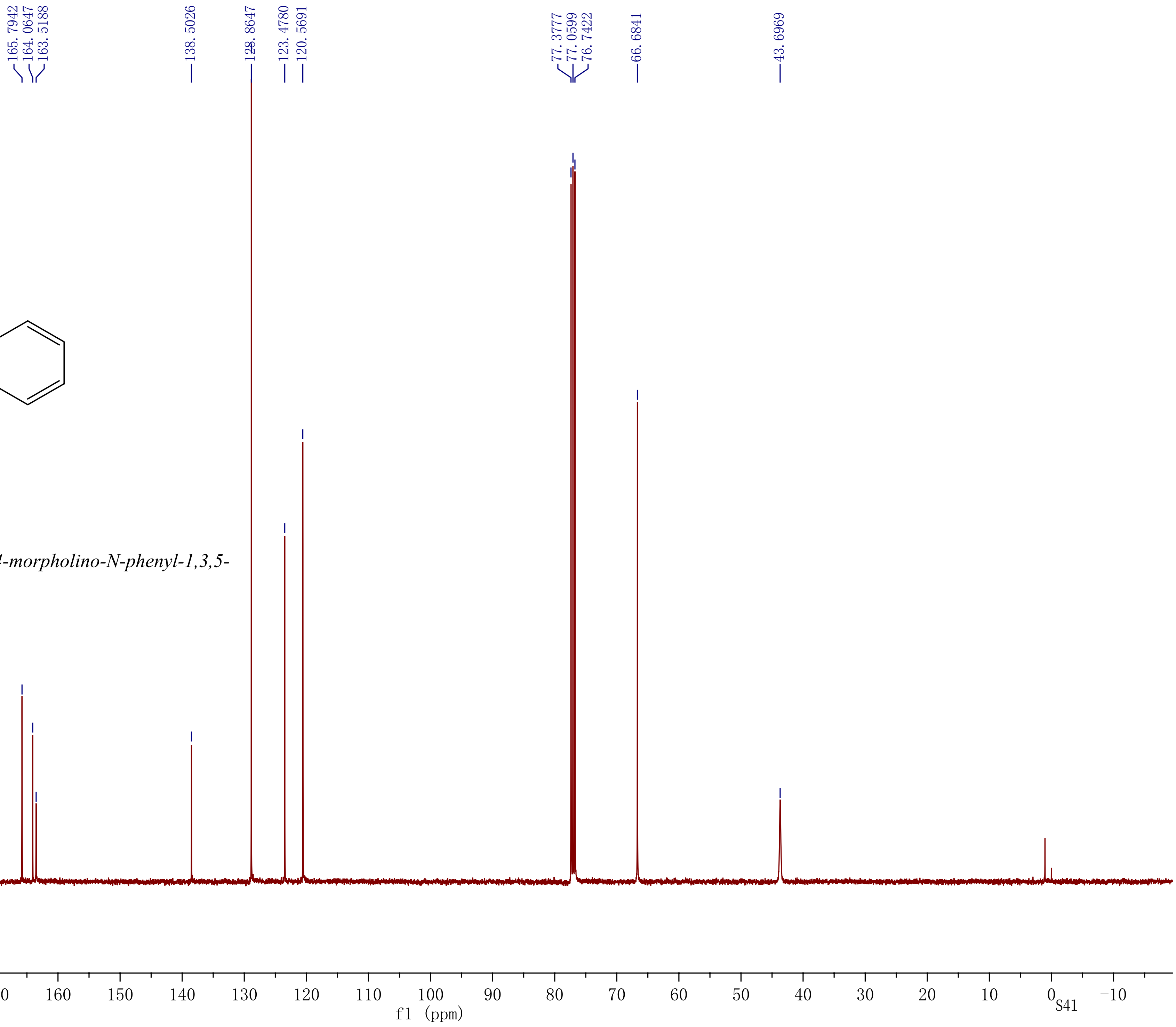

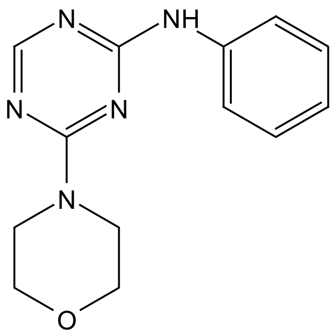

Figure S38, ^{13}C NMR spectrum of *4-morpholino-N-phenyl-1,3,5-triazin-2-amine* **(1j) in CDCl3**

20230412zm
1k CDCl3

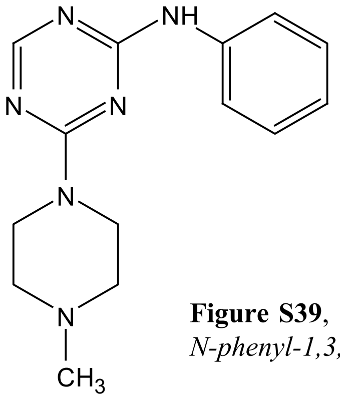

Figure S39, ^{1}H NMR spectrum of *4-(4-methylpiperazin-1-yl)-N-phenyl-1,3,5-triazin-2-amine* **(1k) in CDCl3**

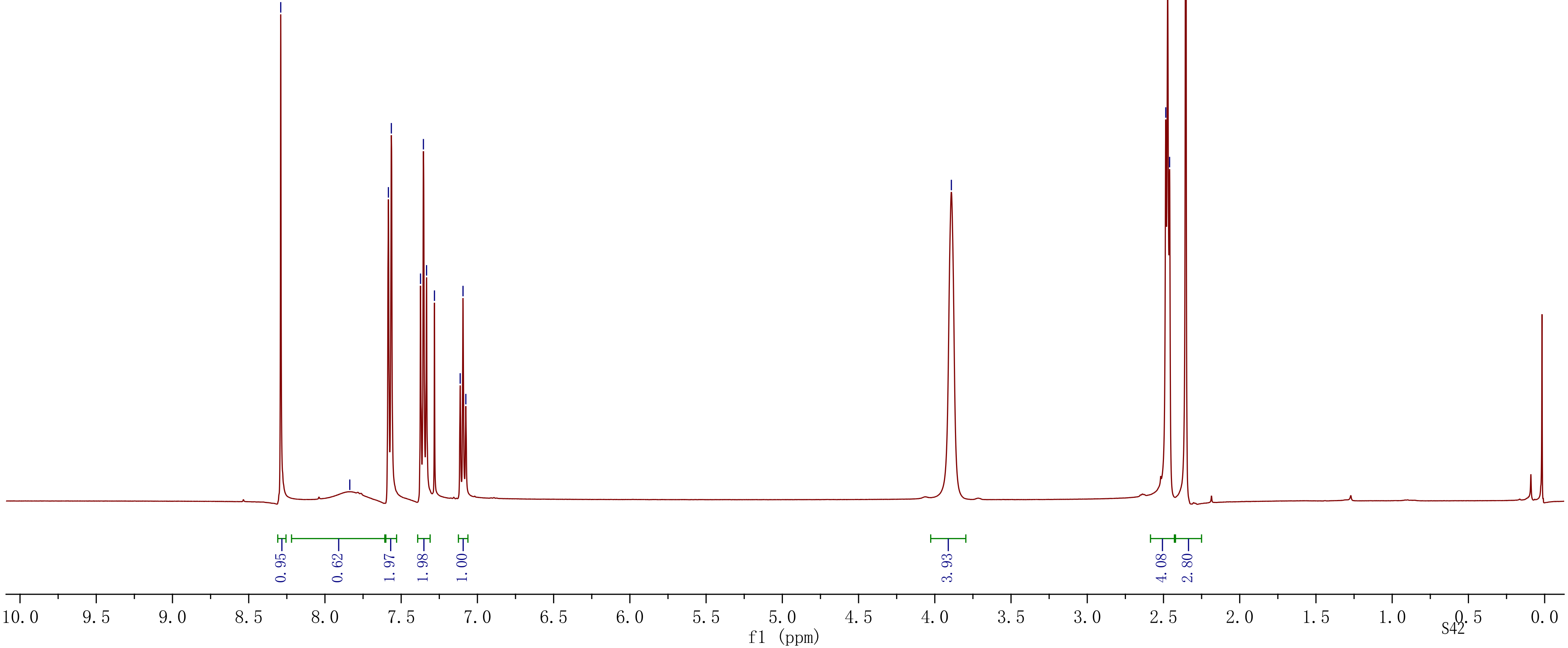

Figure S40, ^{13}C NMR spectrum of *4-(4-methylpiperazin-1-yl)-N-phenyl-1,3,5-triazin-2-amine* **(1k) in CDCl3**

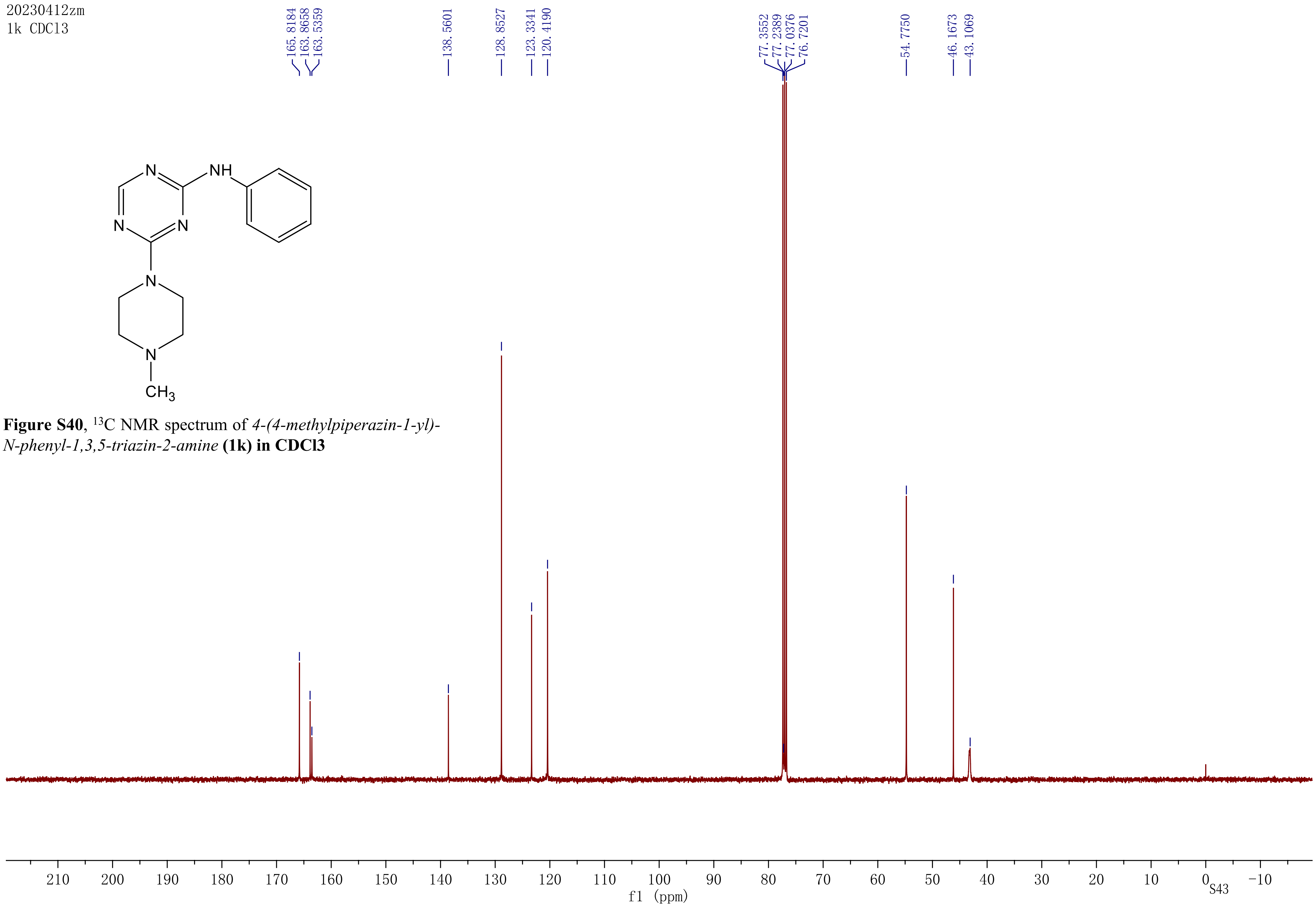

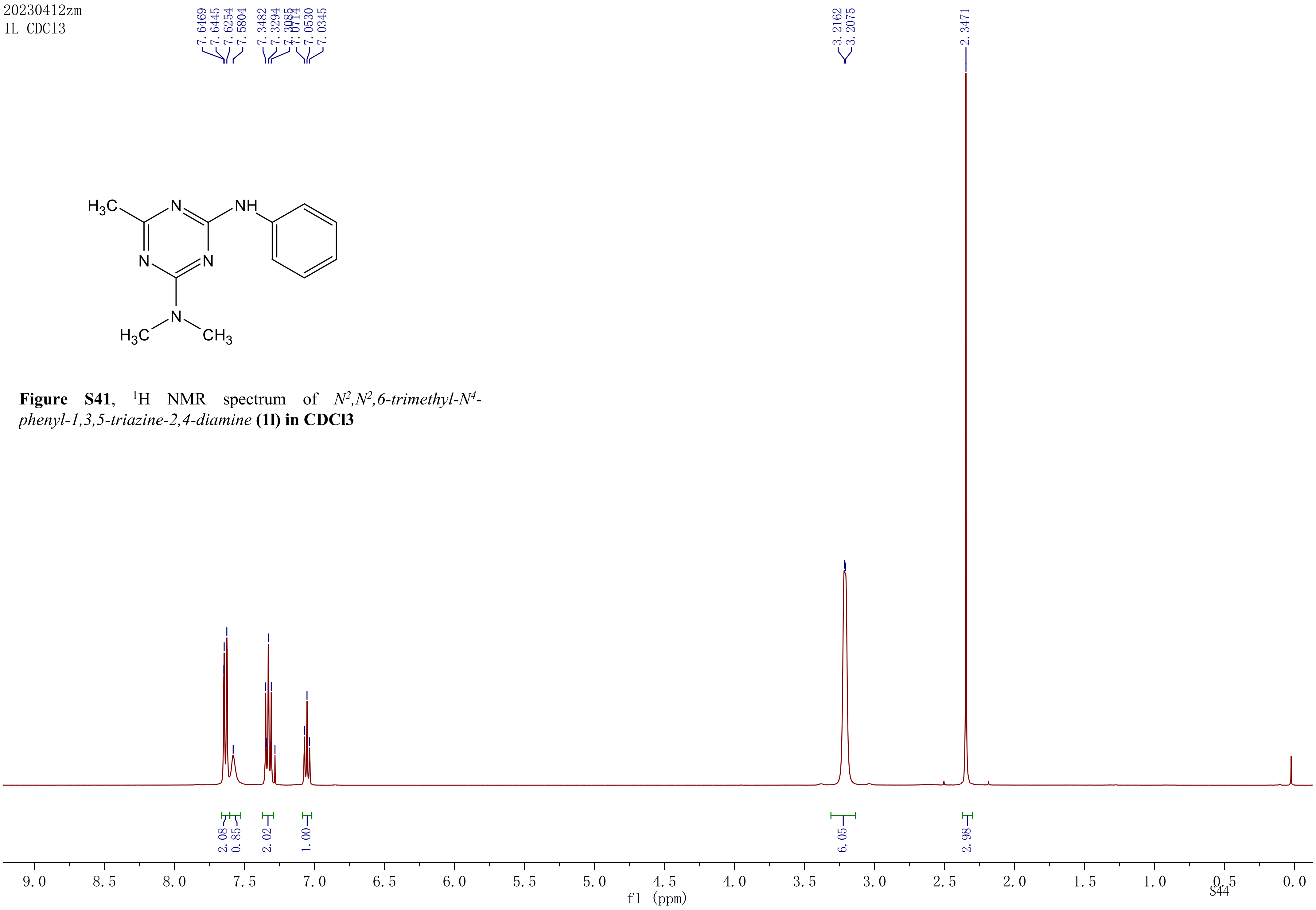

Figure S41, ^{1}H NMR spectrum of *N^2,N^2,6-trimethyl-N^4-phenyl-1,3,5-triazine-2,4-diamine* **(1l) in CDCl3**

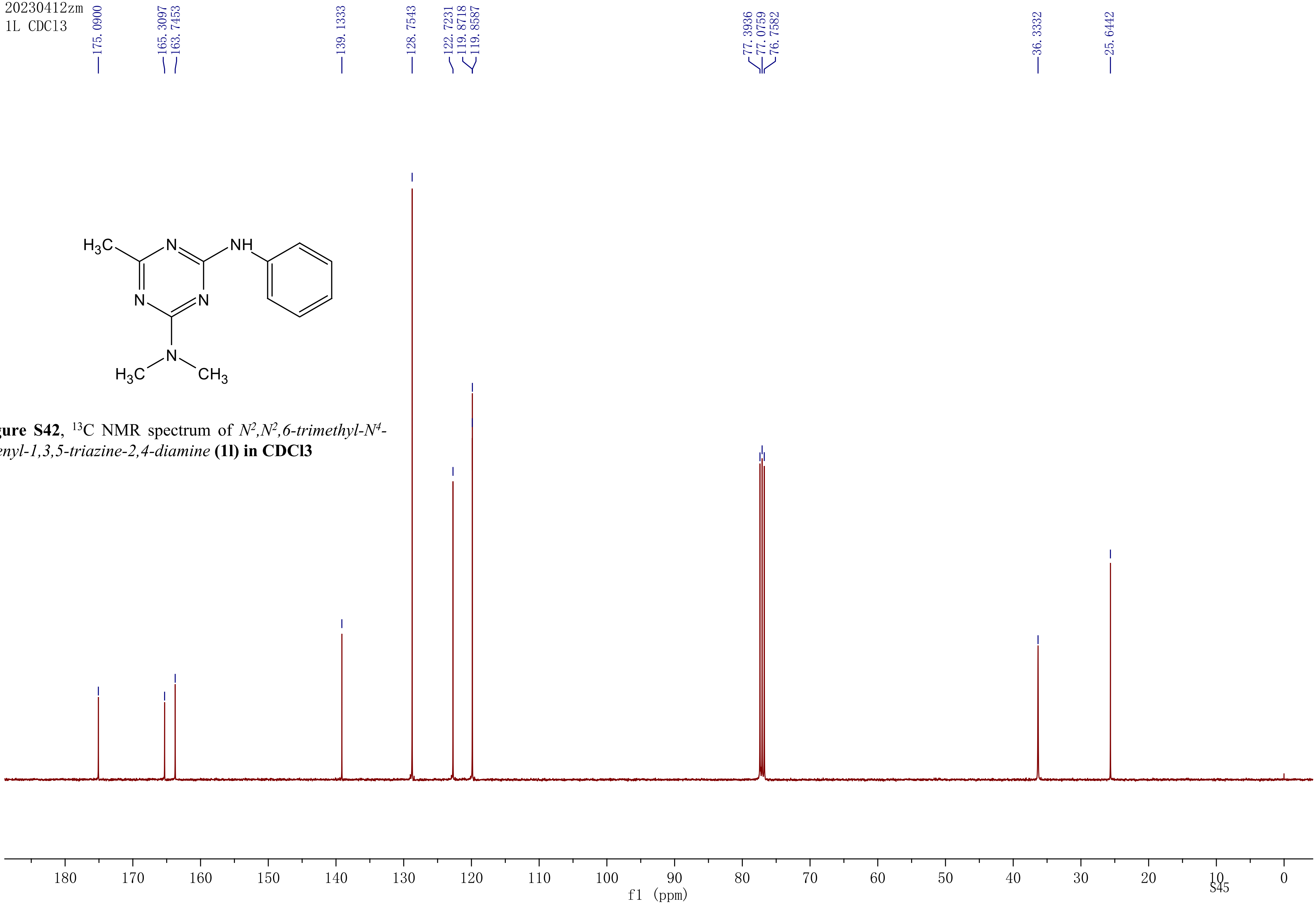

Figure S42, ^{13}C NMR spectrum of *N*^{2}*,N*^{2}*,6-trimethyl-N*^{4}*-phenyl-1,3,5-triazine-2,4-diamine* **(1l) in CDCl3**

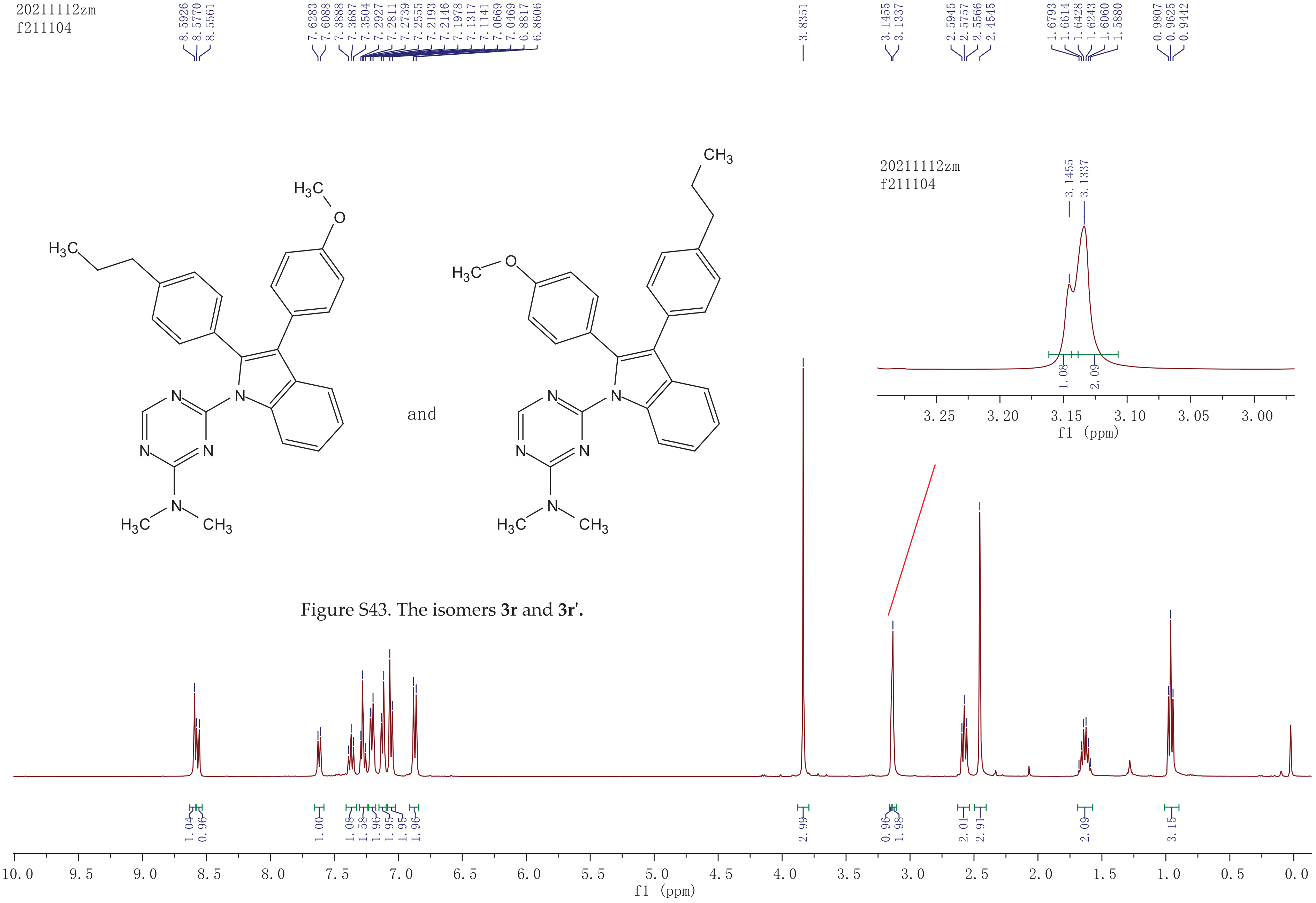

Figure S43. The isomers **3r** and **3r'**.
